# Supplementary material for: The diverse club
Source: Nat Commun. 2017 Nov 2;8:1277. doi: 10.1038/s41467-017-01189-w (PMC5668346; doi:10.1038/s41467-017-01189-w)
Supplement: Supplementary file 1 — Supplementary Information [file 41467_2017_1189_MOESM1_ESM.pdf]

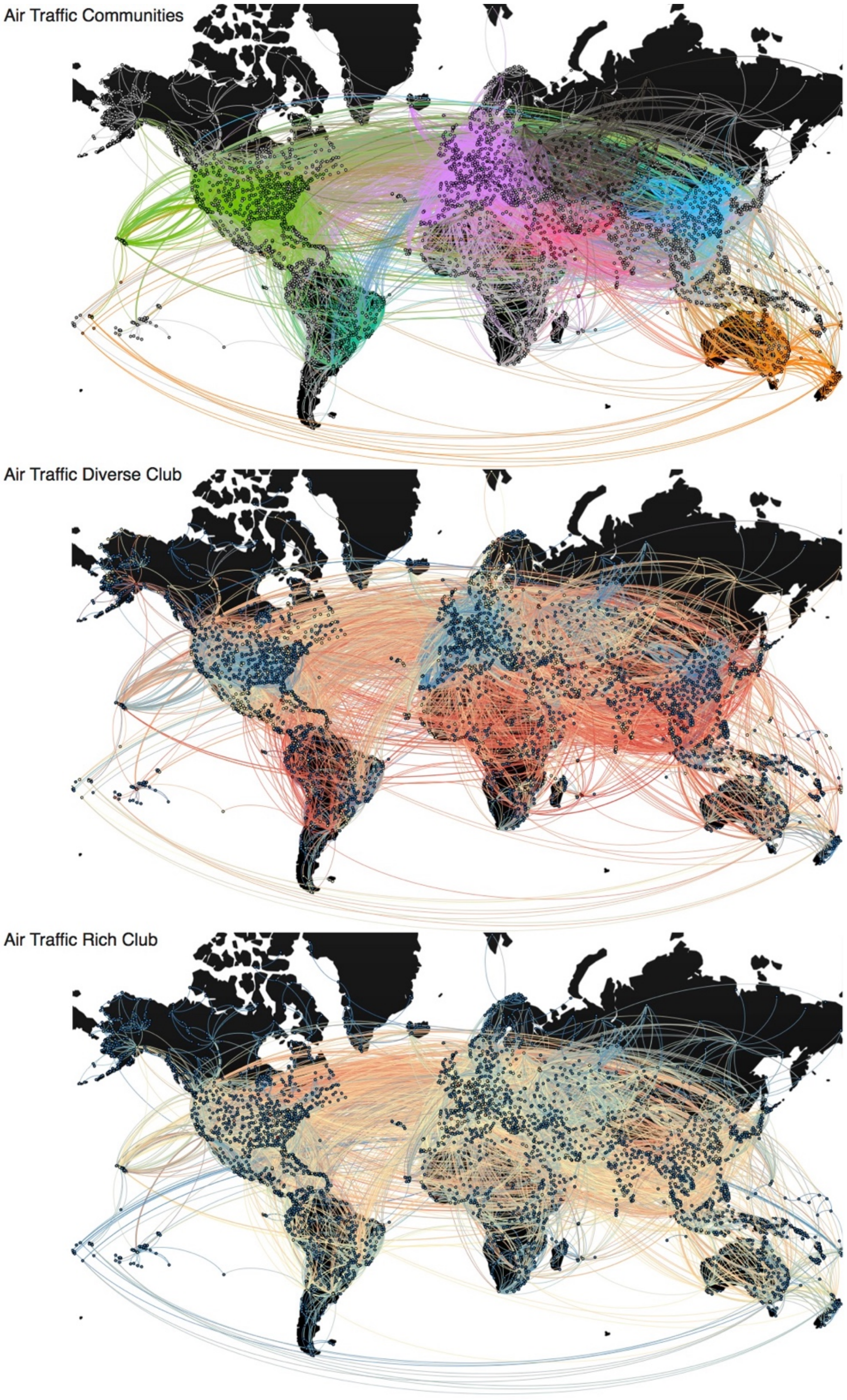

Supplementary Figure 1 I Air traffic communities, diverse club, and rich club. Top, community detection results from the air traffic network. Here, each node is colored according to the community it is in. Middle, diverse club; bottom, rich club. Nodes in red represent the maximum value for the given metric (participation coefficient or strength), yellow is median, and blue is the minimum. Edges are colored by the mix between the two nodes each edge connects. Edges represent a flight route, with red edges being intra club, yellow between a club node and a non-club node, and blue as between two non-club nodes. Note that, only in the diverse club, non-club flights are predominately domestic, with diverse club flights predominately between international airports.

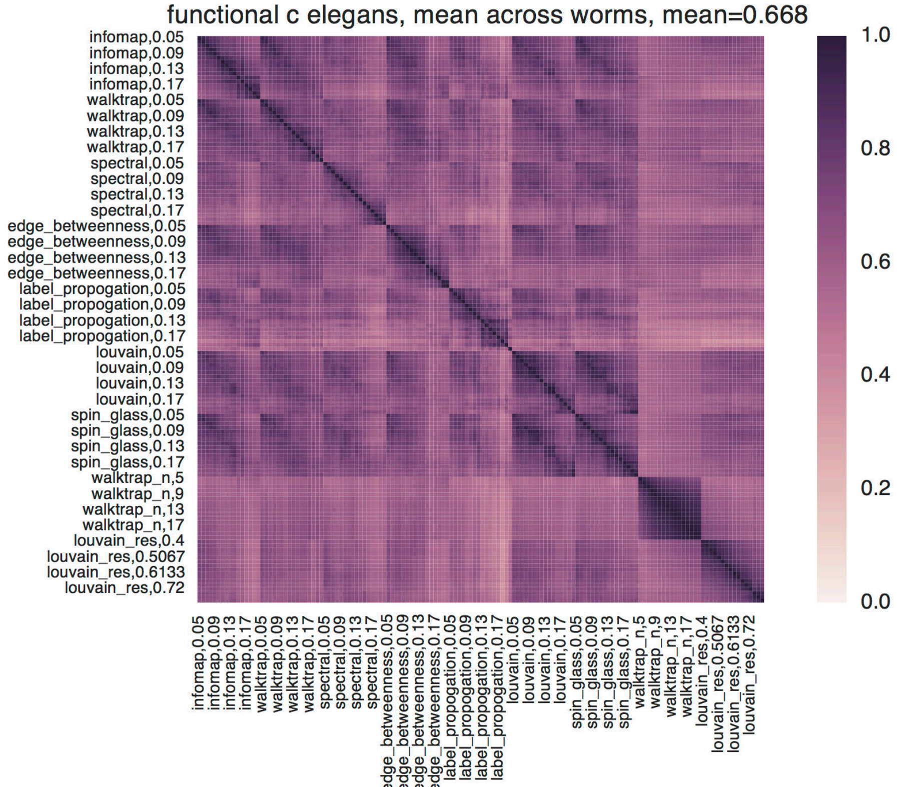

Supplementary Figure 2 I Normalized mutual information across community detection methods in the functional c elegans. Across each community detection method, as well as across graph densities, the number of communities requested (Walktrap N), or the resolution (Louvain Resolution), the normalized mutual information between the community assignments is shown. The mean across the four worms is shown.

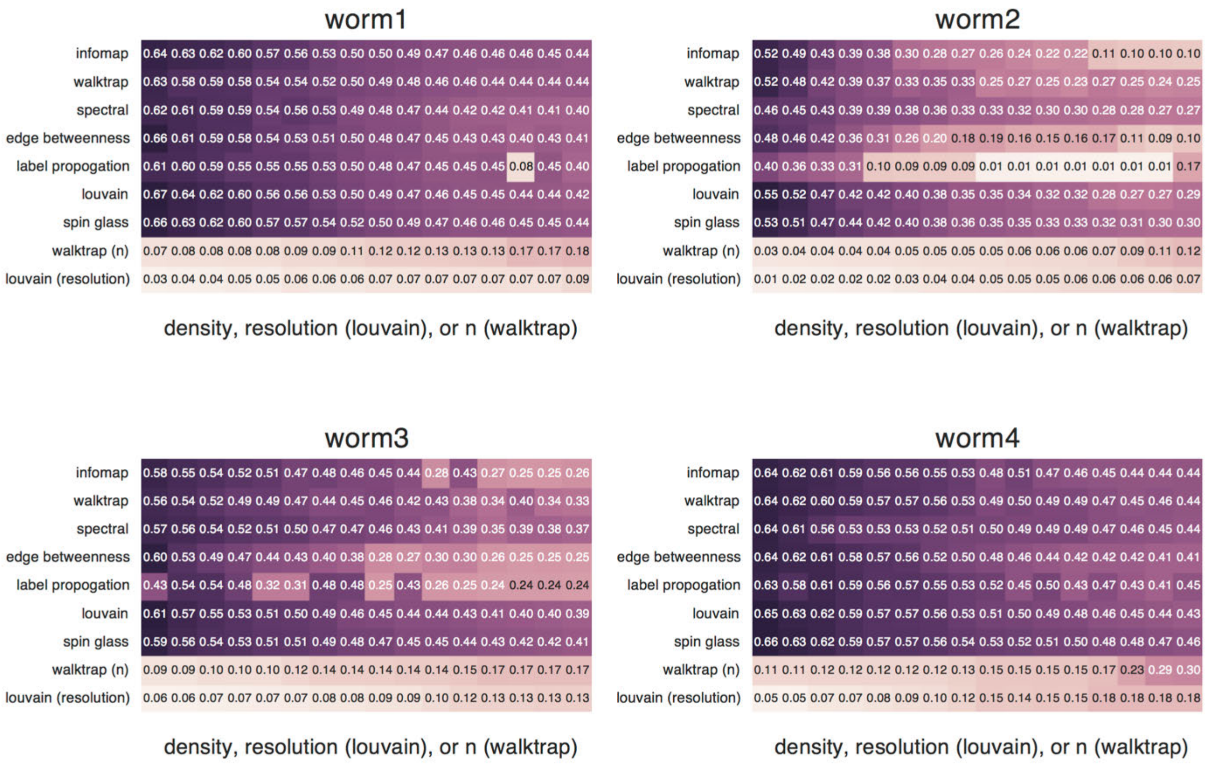

Supplementary Figure 3 I Functional c elegans Q values for each community detection algorithm. For each community detection method, the Q value is shown for each functional c. elegans worm. For each algorithm, the x-axis is ordered from left to right by increasing densities, increasing resolutions (which lead to fewer communities for Louvain Resolution), and decreasing number of communities (Walktrap N).

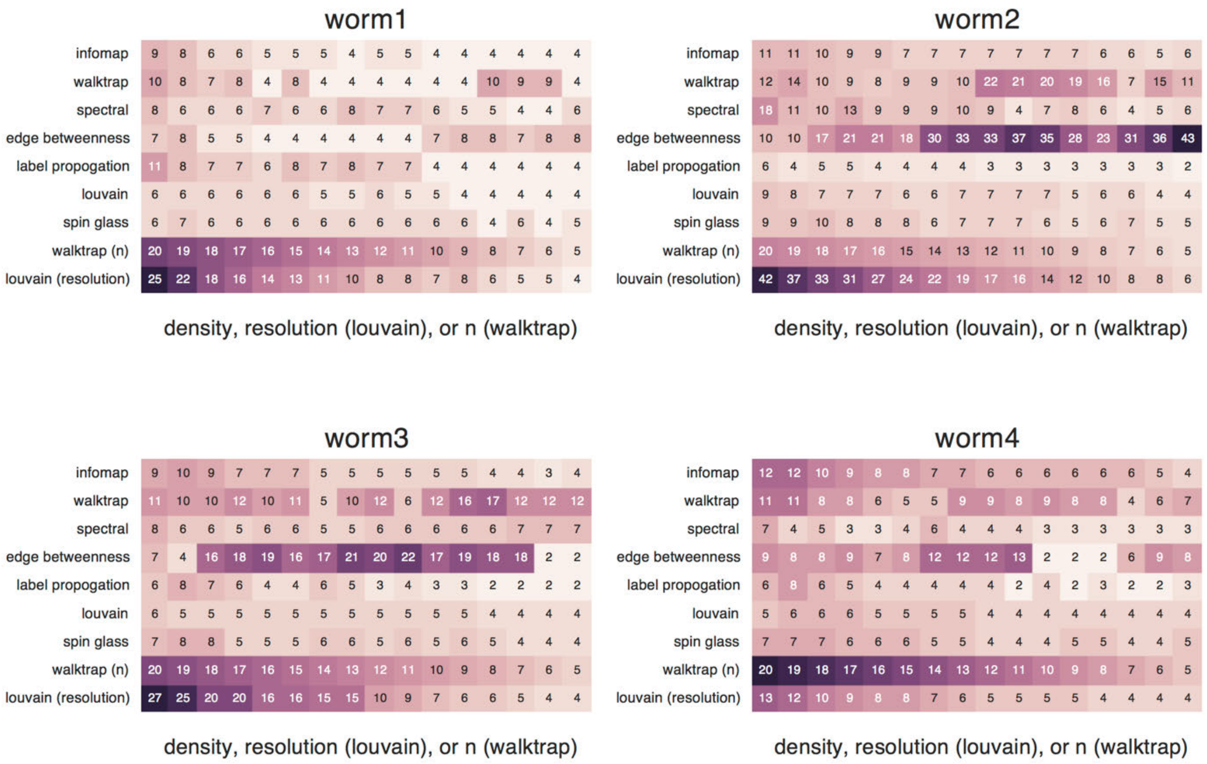

Supplementary Figure 4 I The number of communities for each community detection algorithm in functional c elegans. Across each community detection method, the number of communities is shown for each functional c. elegans worm. For each algorithm, the x-axis is ordered from left to right by increasing densities, increasing resolutions (which lead to fewer communities for Louvain Resolution), and decreasing number of communities (Walktrap N).

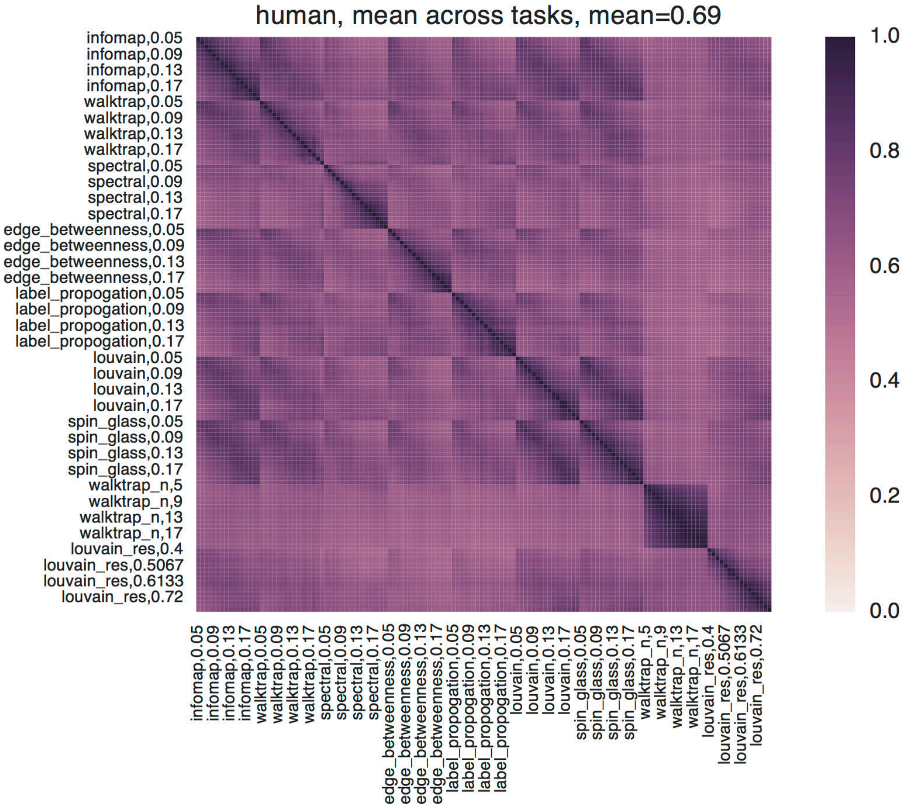

Supplementary Figure 5 I Normalized mutual information across community detection methods in the human. Across each community detection method, as well as across graph densities, the number of communities requested (Walktrap N), or the resolution (Louvain Resolution), the normalized mutual information between the community assignments is shown. The mean across tasks is shown.

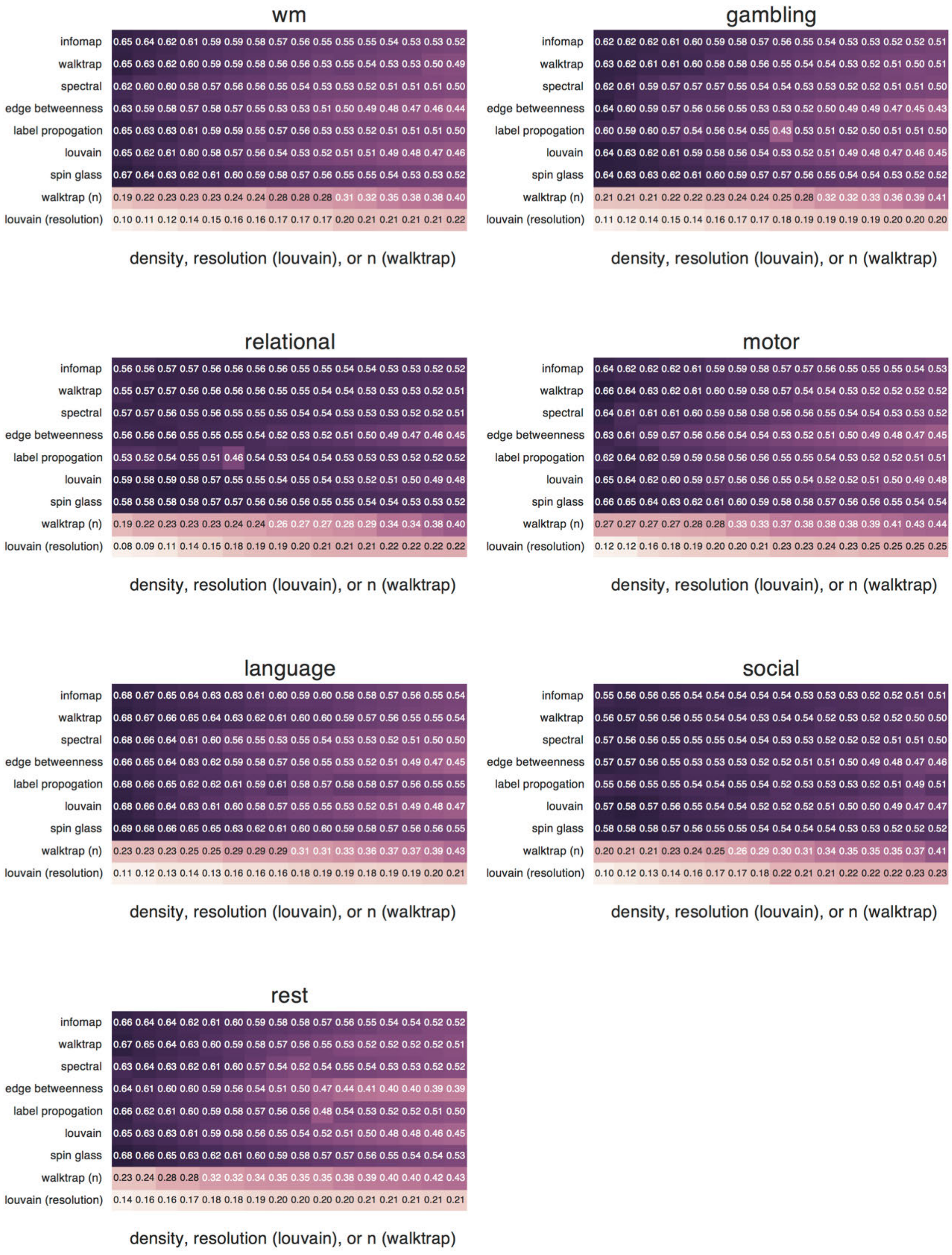

Supplementary Figure 6 I Human *Q* values for each community detection algorithm. For each community detection method, the *Q* value is shown for each task. For each algorithm, the x-axis is ordered from left to right by increasing densities, increasing resolutions (which lead to fewer communities for Louvain Resolution), and decreasing number of communities (Walktrap N).

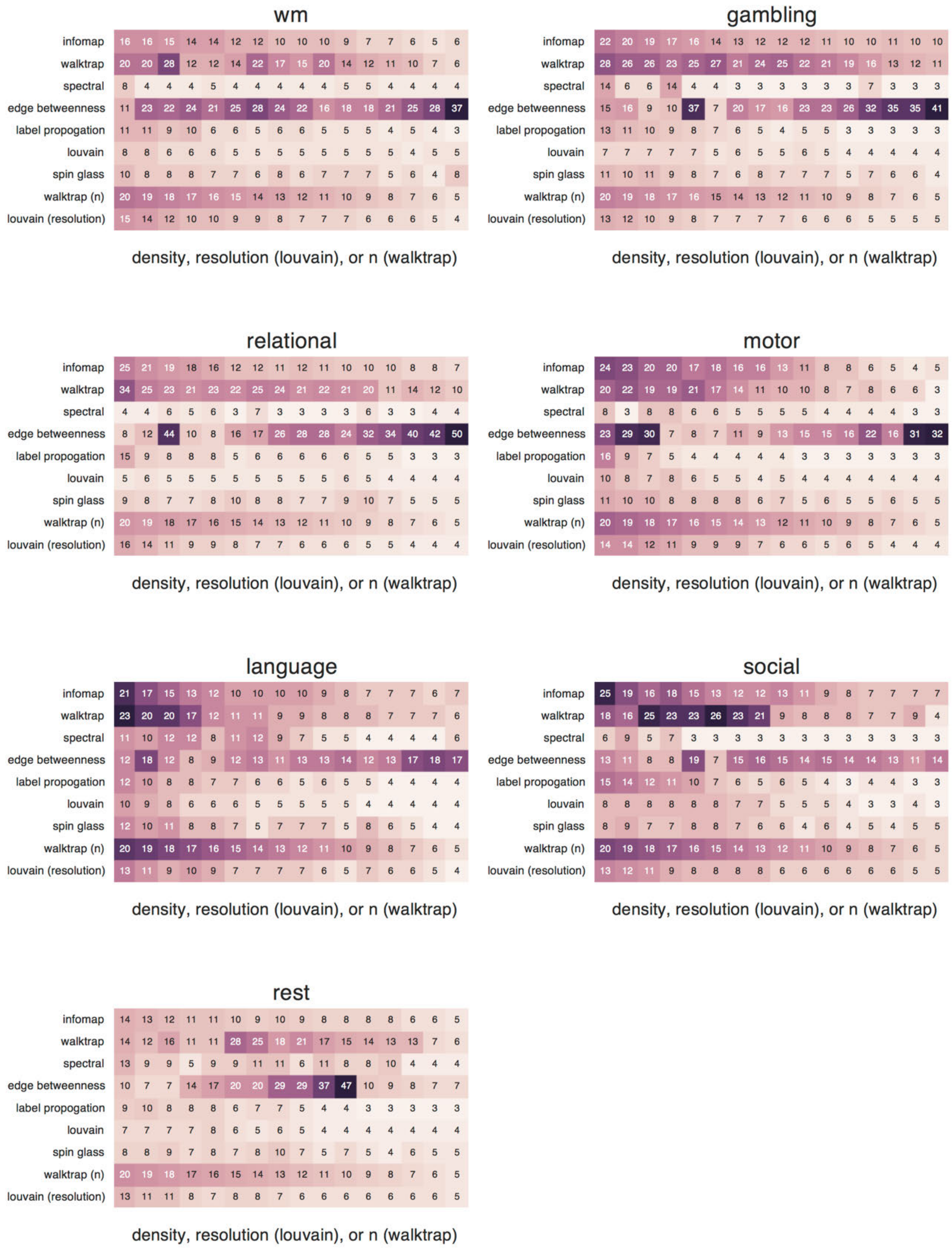

Supplementary Figure 7 I The number of communities for each community detection algorithm in humans. Across each community detection method, the number of communities is shown for each task. For each algorithm, the x-axis is ordered from left to right by increasing densities, increasing resolutions (which lead to fewer communities for Louvain Resolution), and decreasing number of communities (Walktrap N).

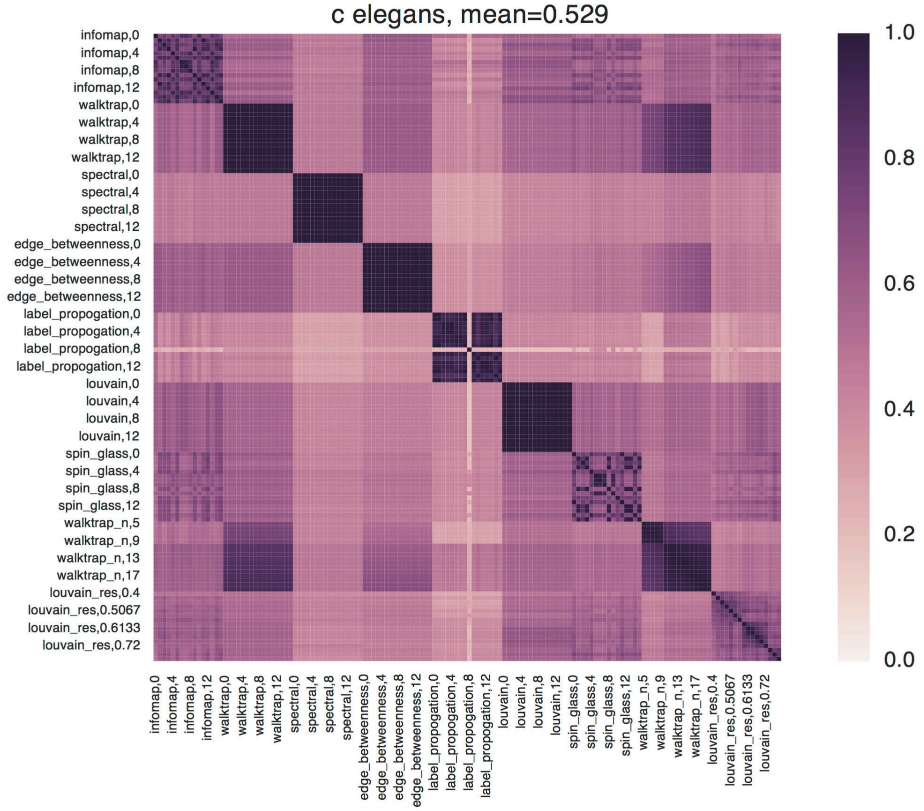

Supplementary Figure 8 I Normalized mutual information across community detection methods in the structural c elegans. Each community detection method was applied 16 times (to match the unique number of densities for the thresholded networks). Here, across community detection methods and runs, the normalized mutual information between the community assignments is shown.

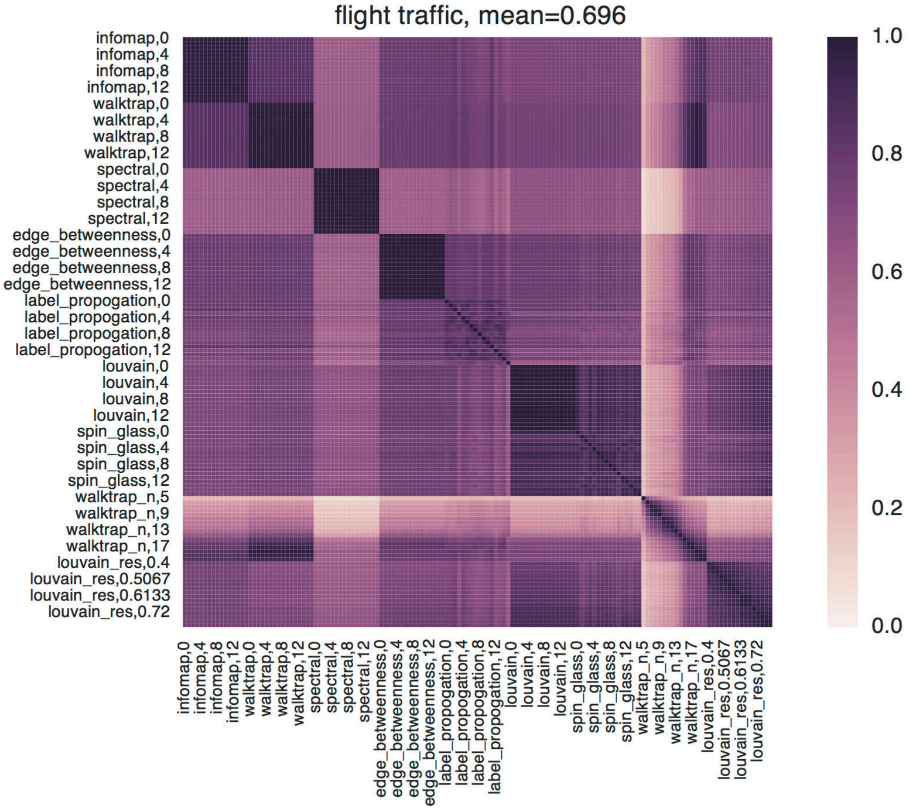

Supplementary Figure 9 I Normalized mutual information across community detection methods in the flight traffic network. Each community detection method was applied 16 times (to match the unique number of densities for the thresholded networks). Here, across community detection methods and runs, the normalized mutual information between the community assignments is shown.

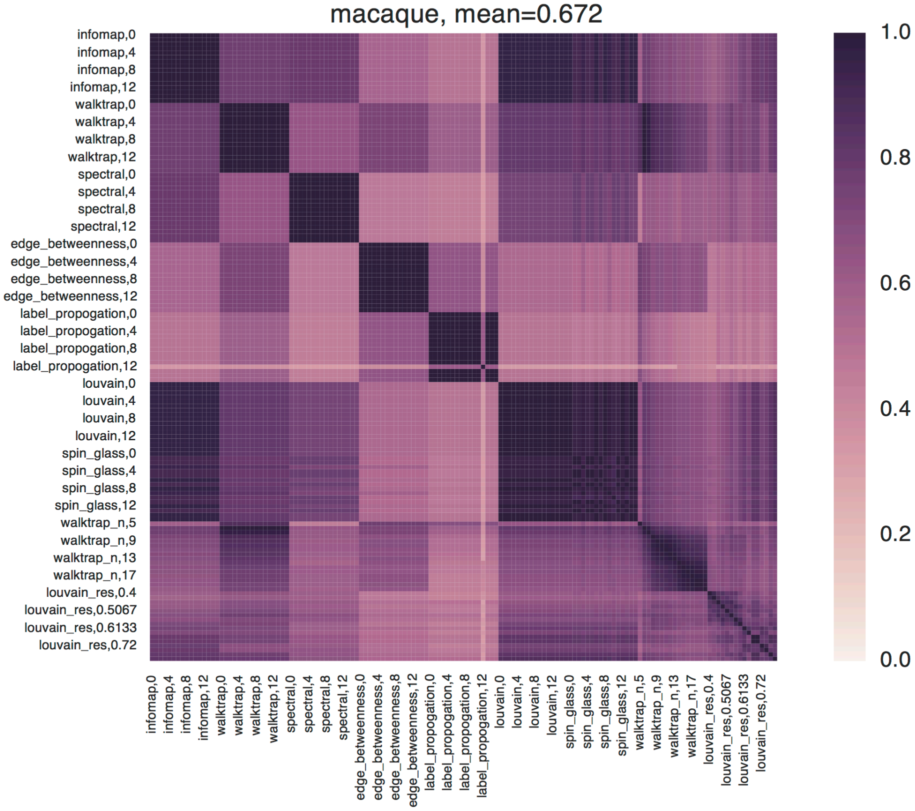

Supplementary Figure 10 I Normalized mutual information across community detection methods in the macaque. Each community detection method was applied 16 times (to match the unique number of densities for the thresholded networks). Here, across community detection methods and runs, the normalized mutual information between the community assignments is shown.

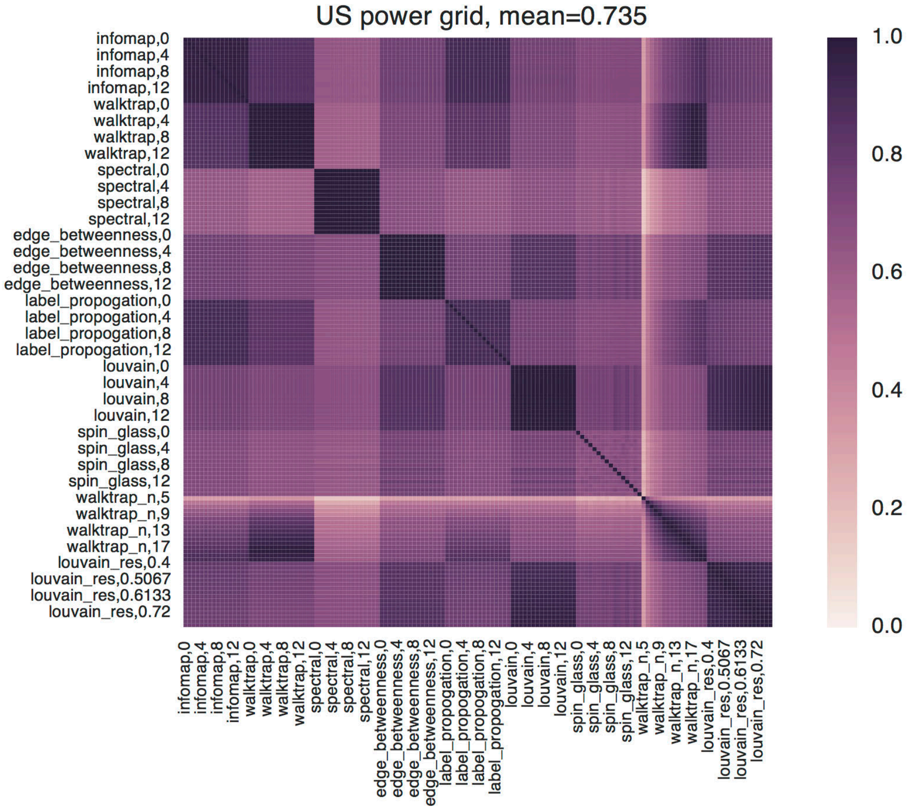

Supplementary Figure 11 I Normalized mutual information across community detection methods in the US power grid network. Each community detection method was applied 16 times (to match the unique number of densities for the thresholded networks). Here, across community detection methods and runs, the normalized mutual information between the community assignments is shown.

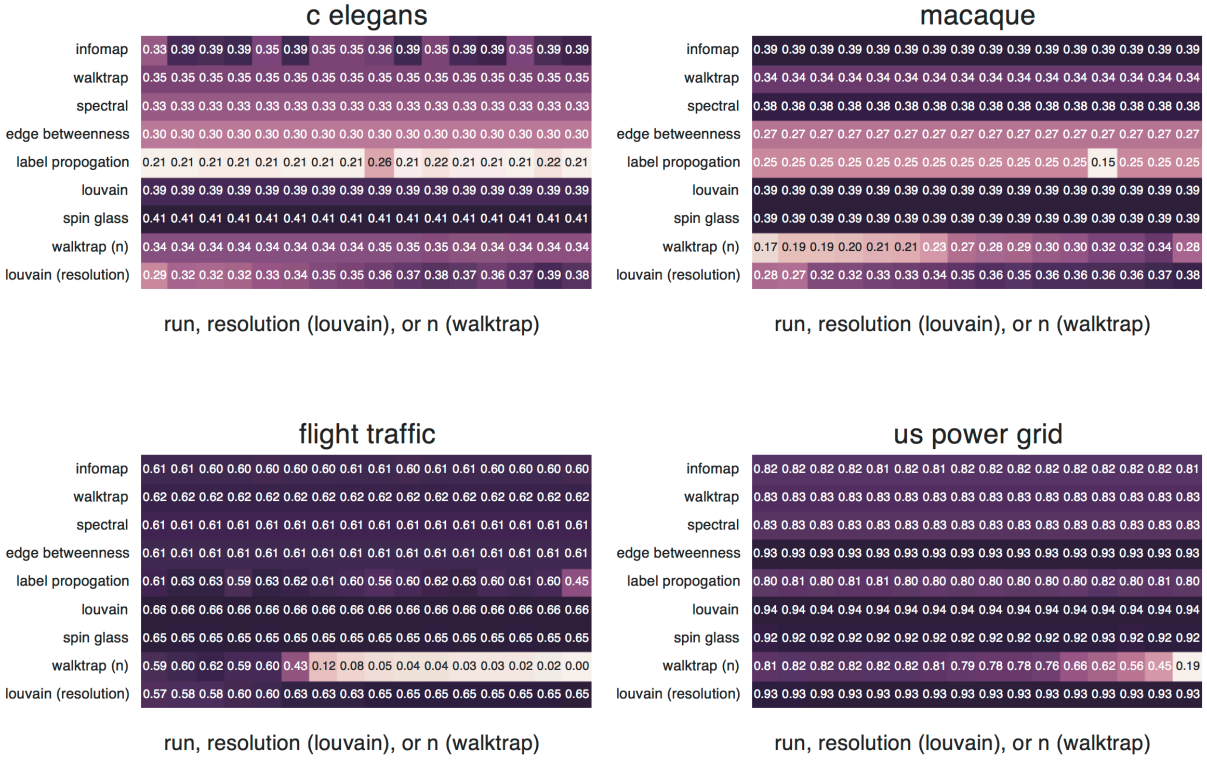

Supplementary Figure 12 l Structural network *Q* values for each community detection algorithm. For each community detection method, the *Q* value is shown for each network and run. For the Louvain Resolution and Walktrap N method, the x-axis is ordered from left to right by increasing resolutions (which lead to fewer communities for Louvain Resolution) and decreasing number of communities (Walktrap N).

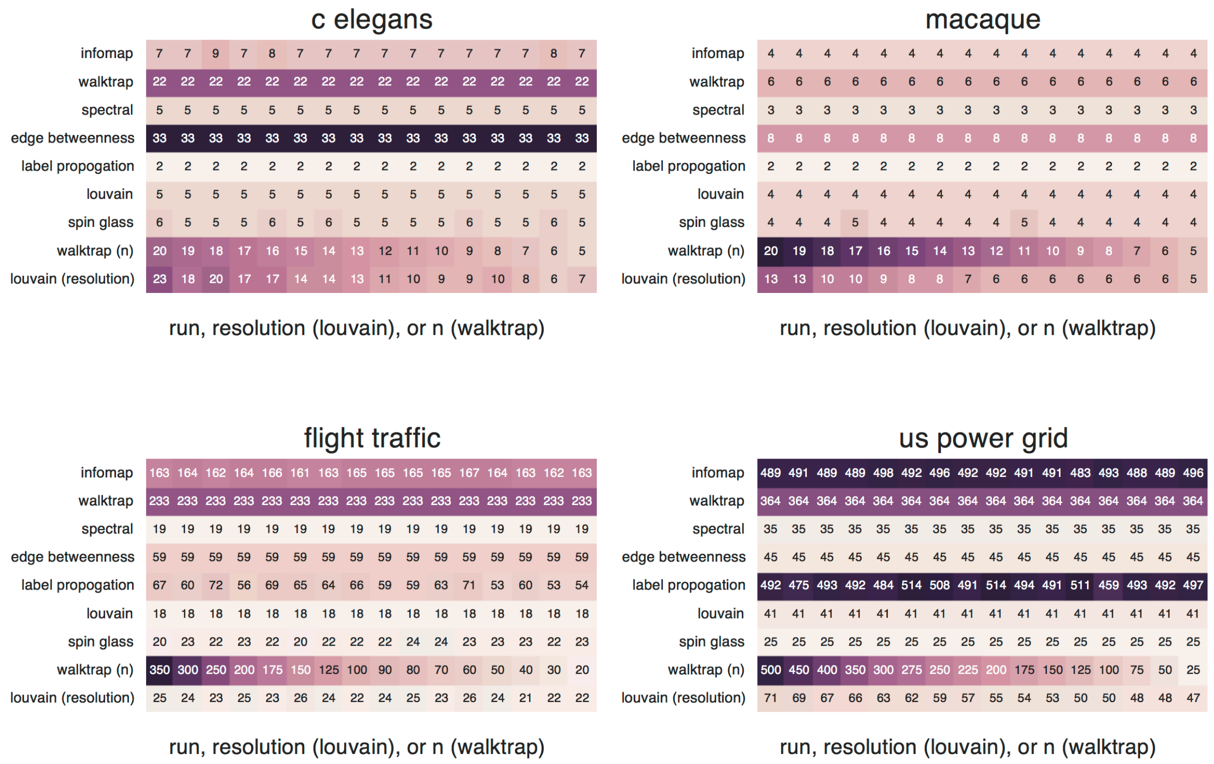

Supplementary Figure 13 | The number of communities for each community detection algorithm in structural networks. For each community detection method, the number of communities is shown for each network and run. For the Louvain Resolution and Walktrap N method, the x-axis is ordered from left to right by increasing resolutions (which lead to fewer communities for Louvain Resolution) and decreasing number of communities (Walktrap N).

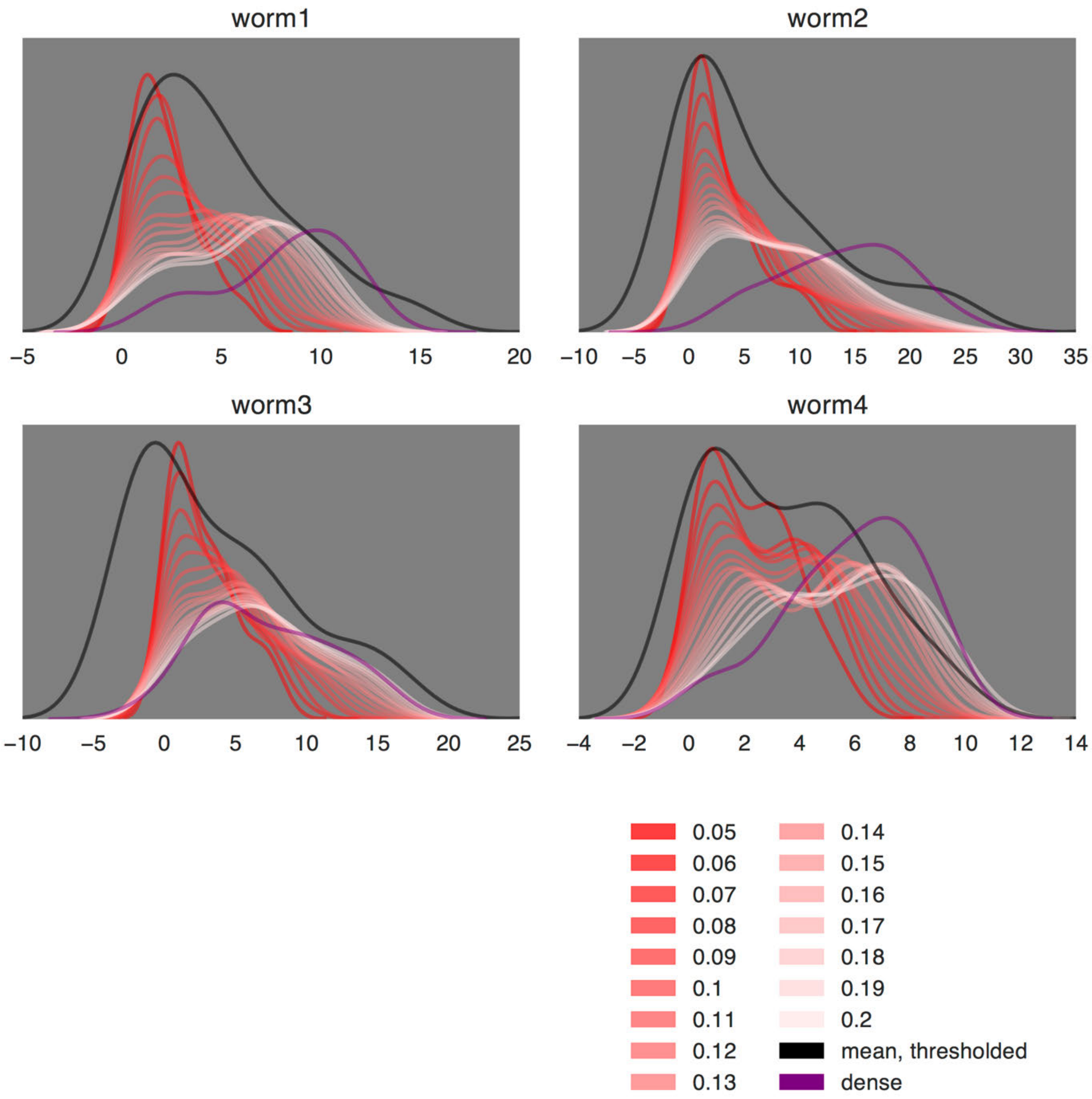

Supplementary Figure 14 I Strength distribution of functional c elegans network. For each worm's network, for each density (including the unthresholded network, dense), a kernel density estimate was fit to the network's strength distribution and is plotted. The "mean" is the mean kernel density estimate for the thresholded networks.

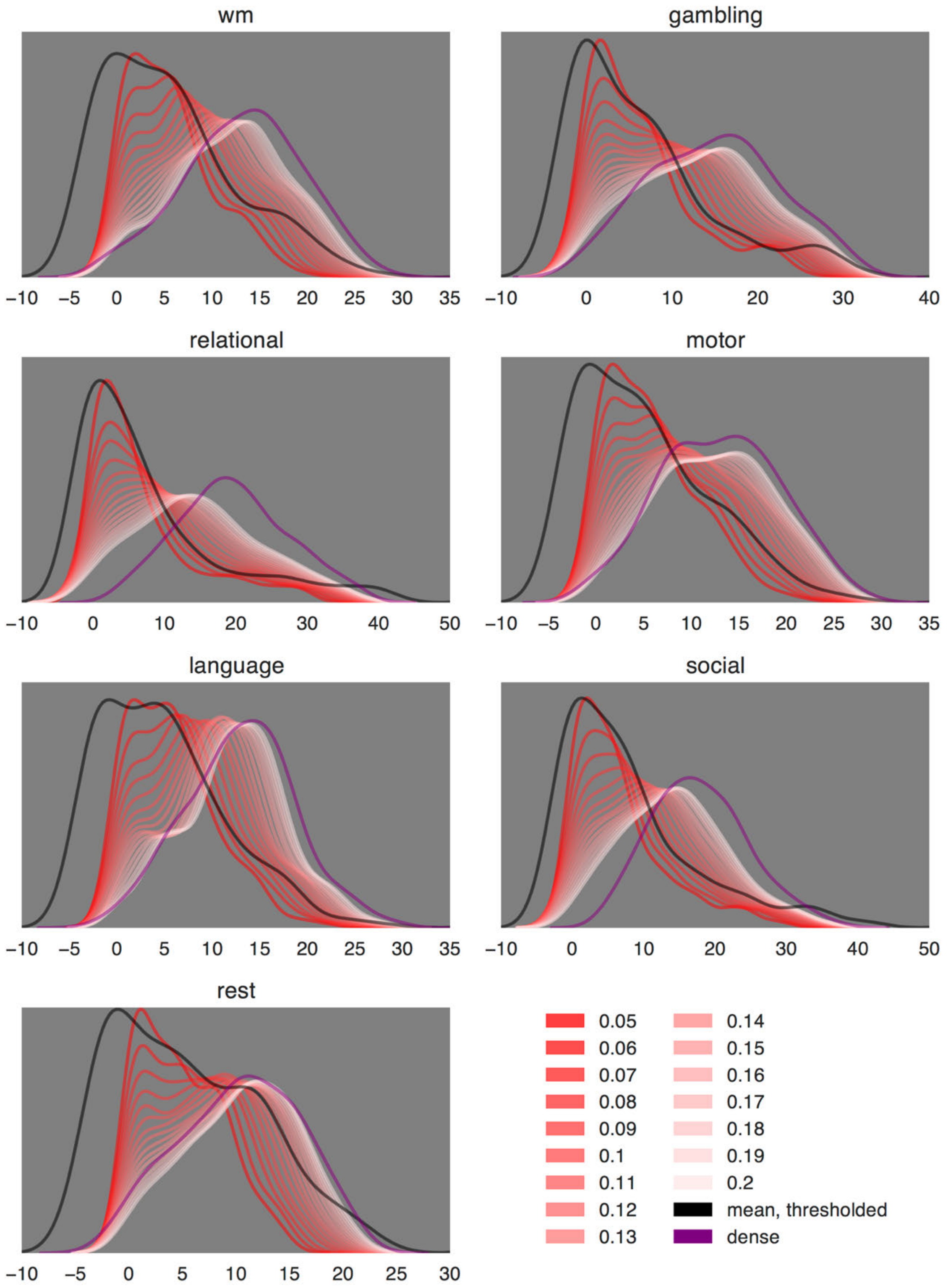

Supplementary Figure 15 | Strength distribution of human network. For each task's network, for each density (including the unthresholded network, dense), a kernel density estimate was fit to the network's strength distribution and is plotted. The "mean" is the mean kernel density estimate for the thresholded networks.

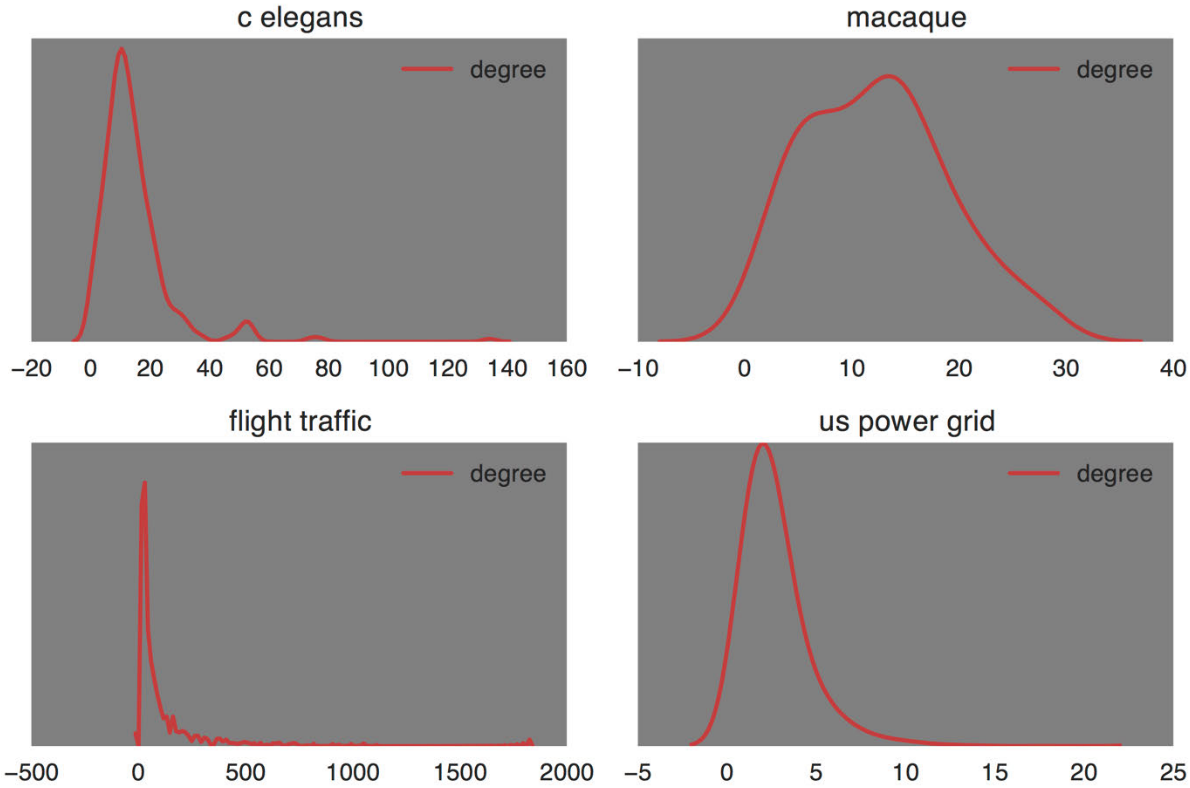

Supplementary Figure 16 | Strength distribution of structural networks. For each structural network a kernel density estimate was fit to the network's strength distribution and is plotted.

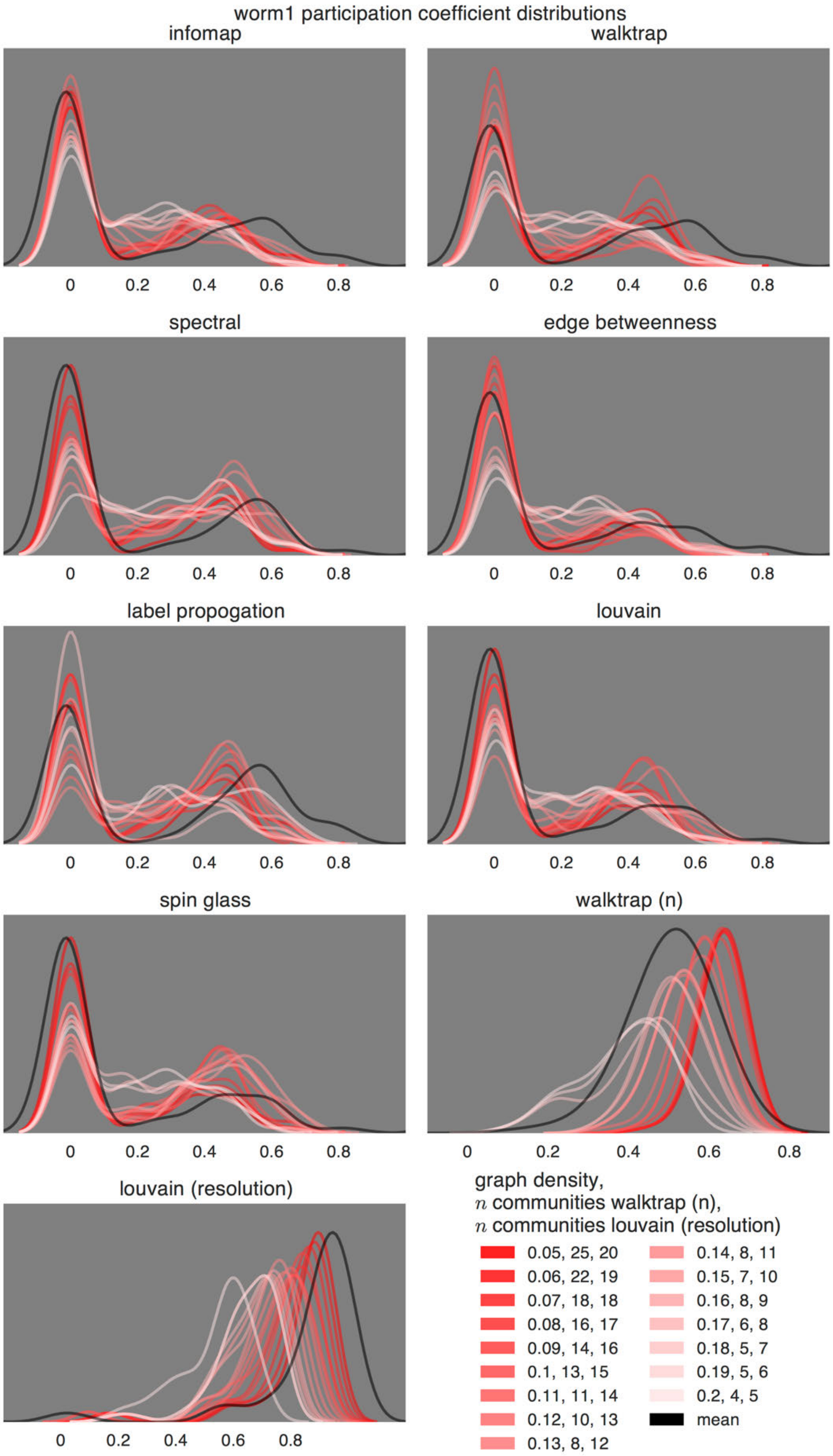

Supplementary Figure 17 I Participation coefficient distribution of the functional c elegans network. For this worm's network and each community detection method, for each density, number of communities, or resolution, a kernel density estimate was fit to the network's participation coefficient distribution and is plotted. The "mean" is the mean kernel density estimate.

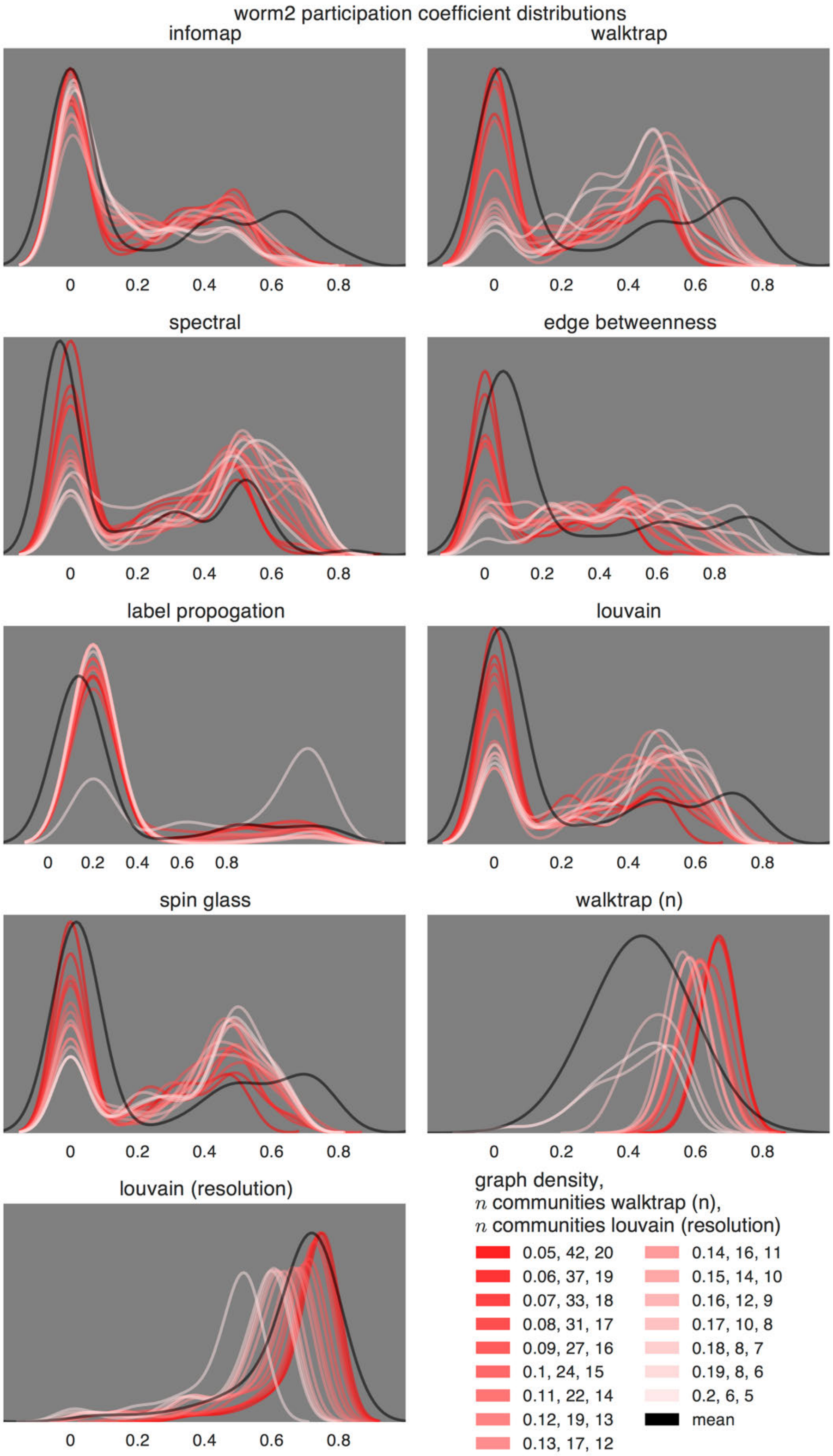

Supplementary Figure 18 I Participation coefficient distribution of the functional c elegans network. For this worm's network and each community detection method, for each density, number of communities, or resolution, a kernel density estimate was fit to the network's participation coefficient distribution and is plotted. The "mean" is the mean kernel density estimate.

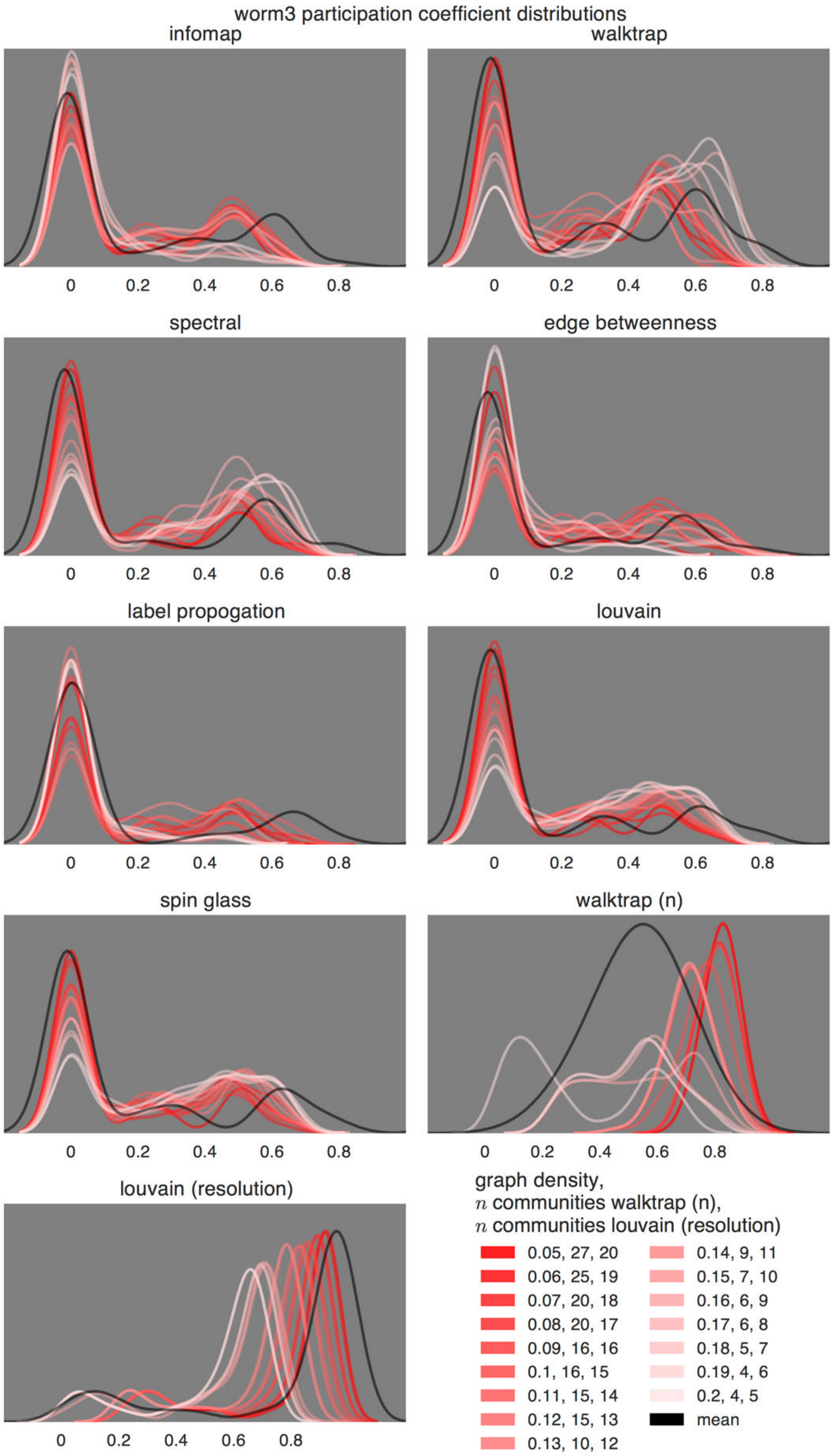

Supplementary Figure 19 I Participation coefficient distribution of the functional c elegans network. For this worm's network and each community detection method, for each density, number of communities, or resolution, a kernel density estimate was fit to the network's participation coefficient distribution and is plotted. The "mean" is the mean kernel density estimate.

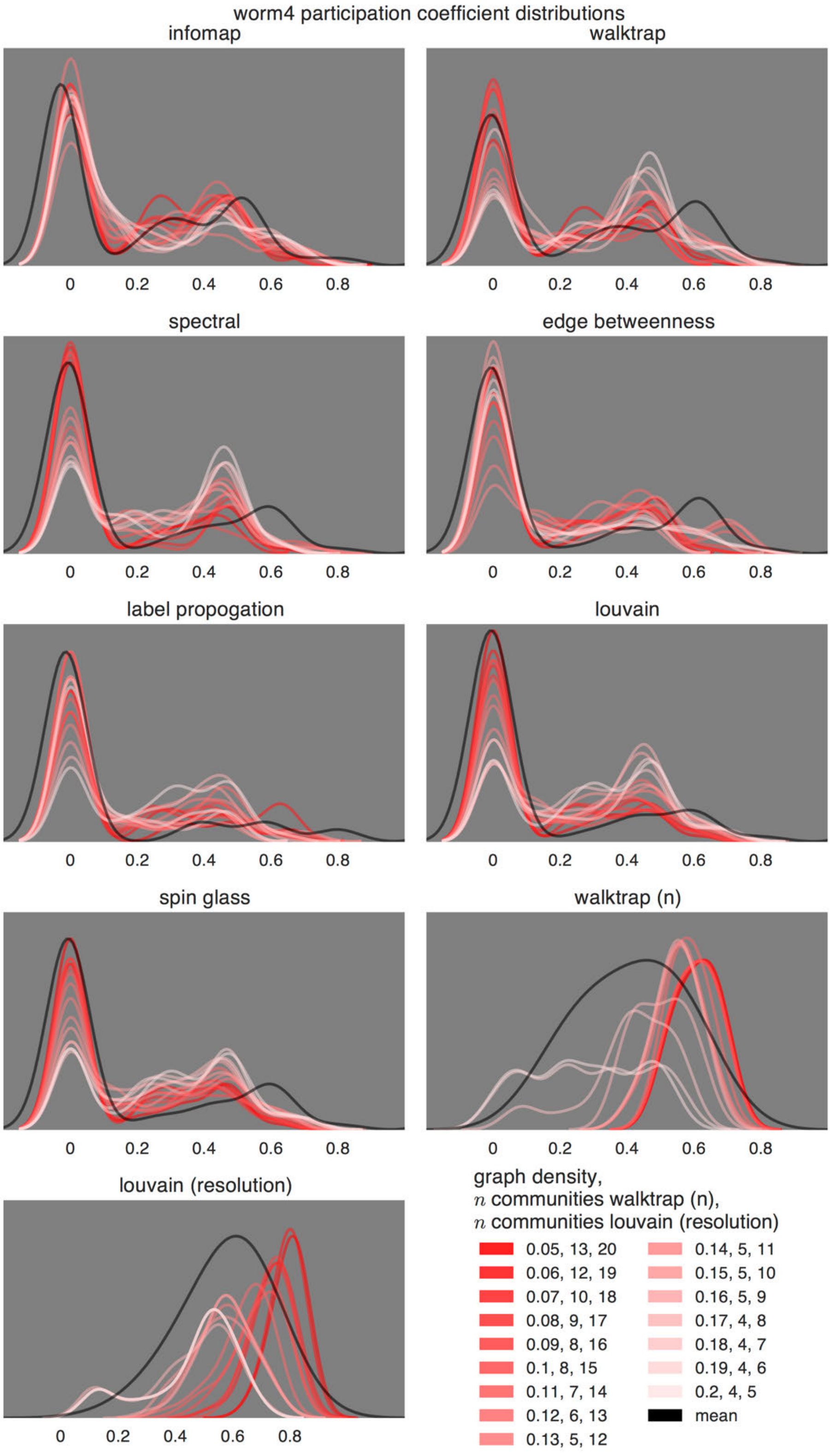

Supplementary Figure 20 I Participation coefficient distribution of the functional c elegans network. For this worm's network and each community detection method, for each density, number of communities, or resolution, a kernel density estimate was fit to the network's participation coefficient distribution and is plotted. The "mean" is the mean kernel density estimate.

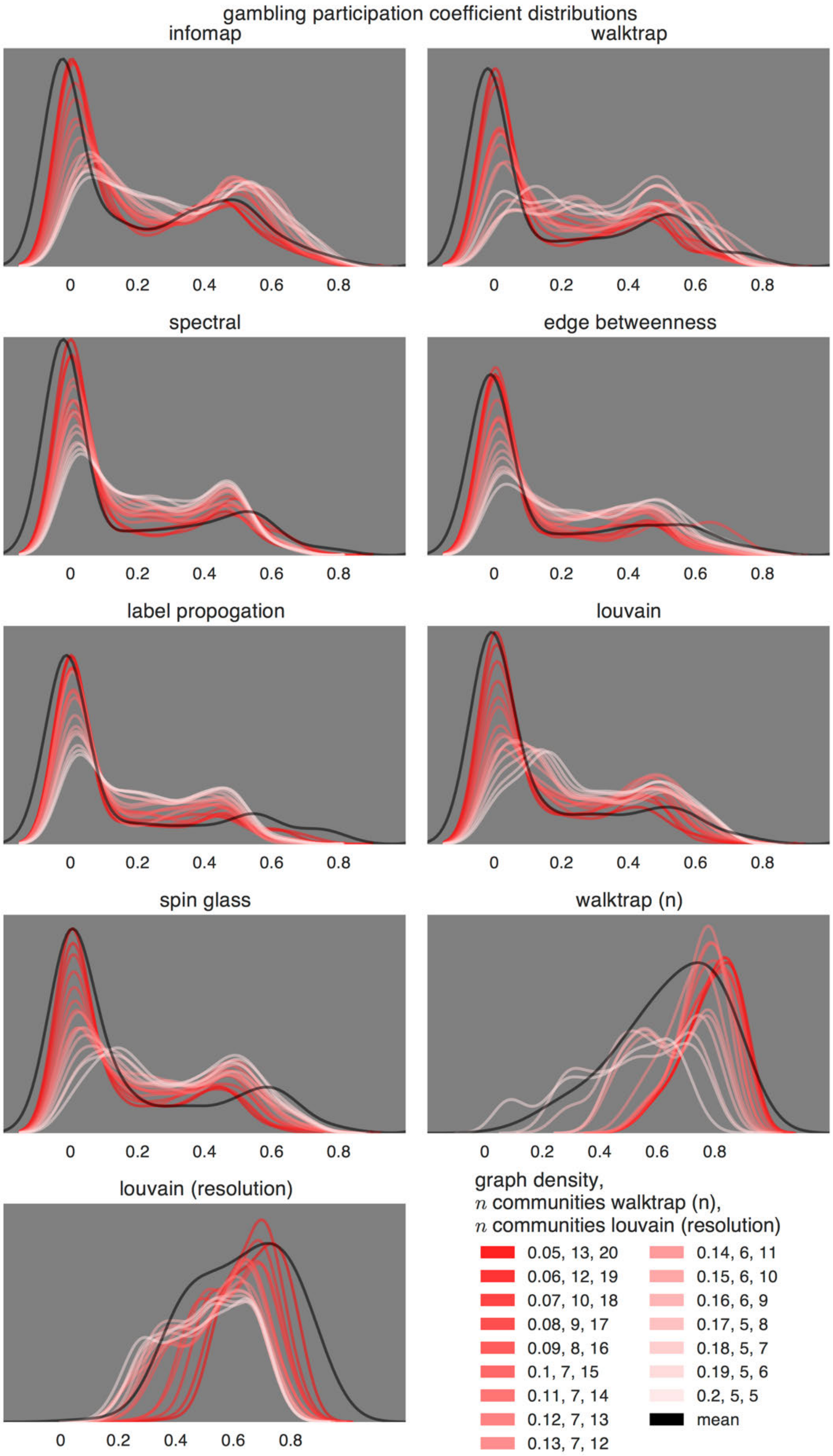

Supplementary Figure 21 I Participation coefficient distribution of the human gambling network. For this task's network and each community detection method, for each density, number of communities, or resolution, a kernel density estimate was fit to the network's participation coefficient distribution and is plotted. The "mean" is the mean kernel density estimate.

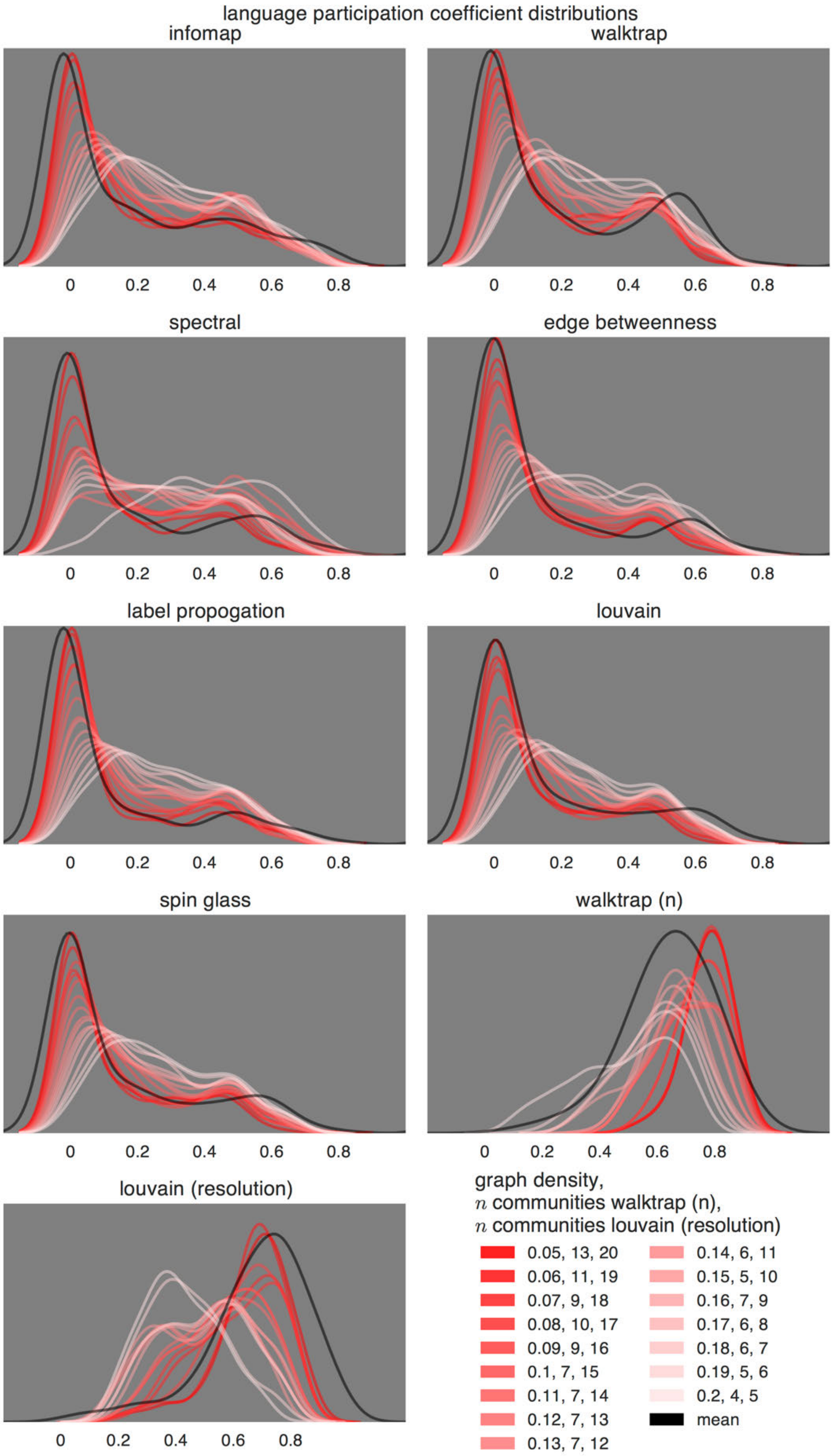

Supplementary Figure 22 I Participation coefficient distribution of the human language network. For this task's network and each community detection method, for each density, number of communities, or resolution, a kernel density estimate was fit to the network's participation coefficient distribution and is plotted. The "mean" is the mean kernel density estimate.

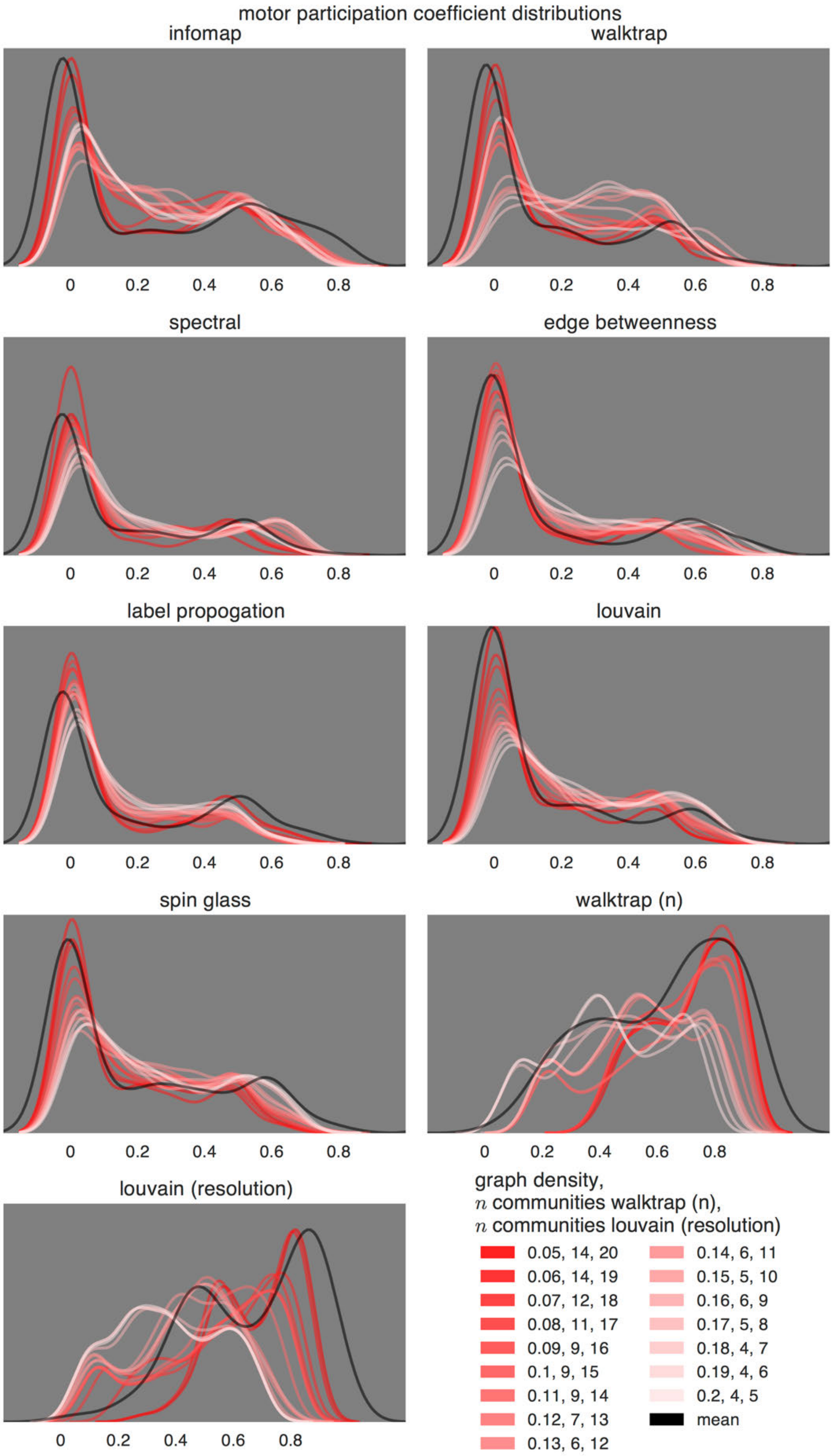

Supplementary Figure 23 I Participation coefficient distribution of the human motor network. For this task's network and each community detection method, for each density, number of communities, or resolution, a kernel density estimate was fit to the network's participation coefficient distribution and is plotted. The "mean" is the mean kernel density estimate.

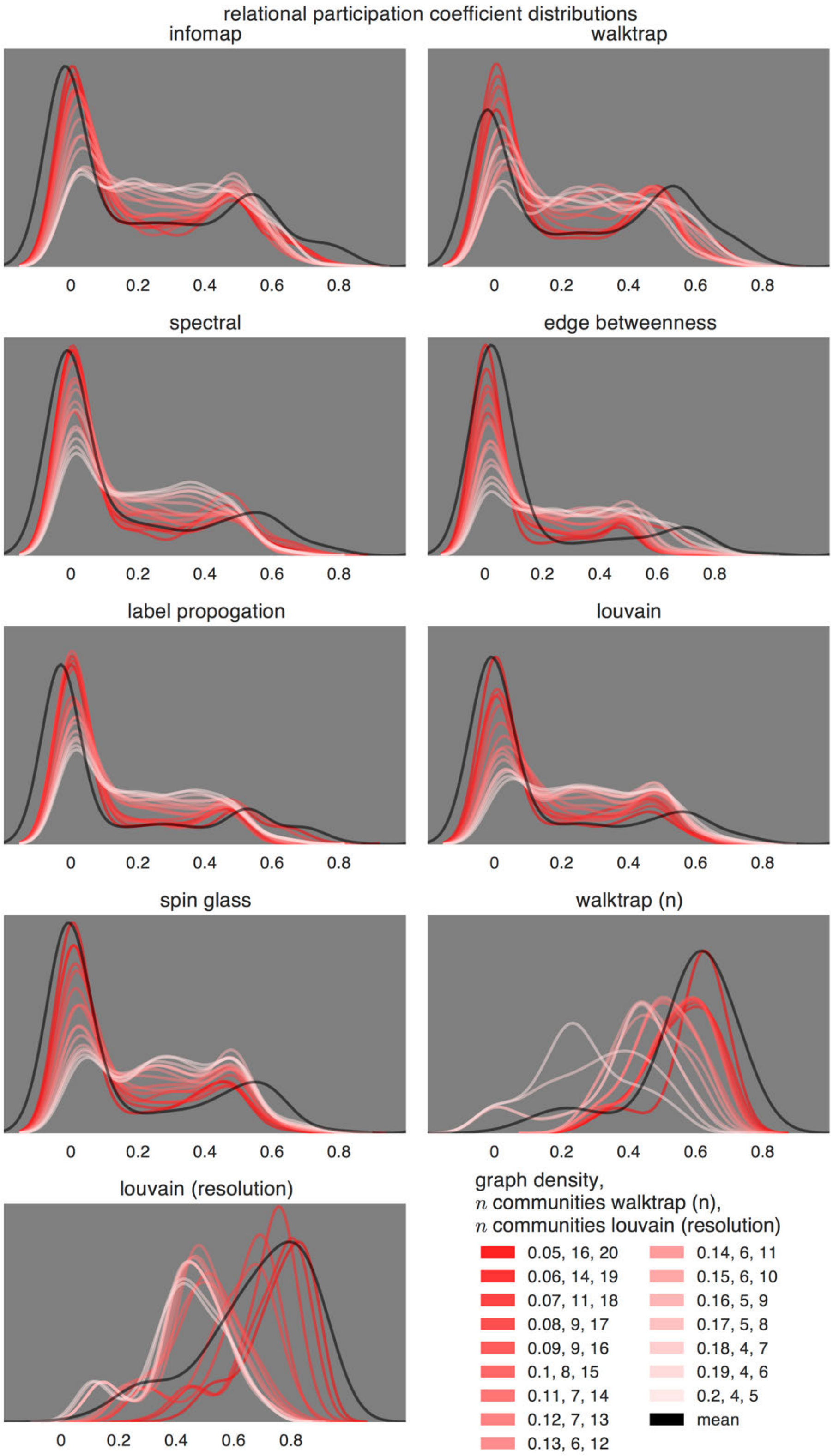

Supplementary Figure 24 I Participation coefficient distribution of the human relational network. For this task's network and each community detection method, for each density, number of communities, or resolution, a kernel density estimate was fit to the network's participation coefficient distribution and is plotted. The "mean" is the mean kernel density estimate.

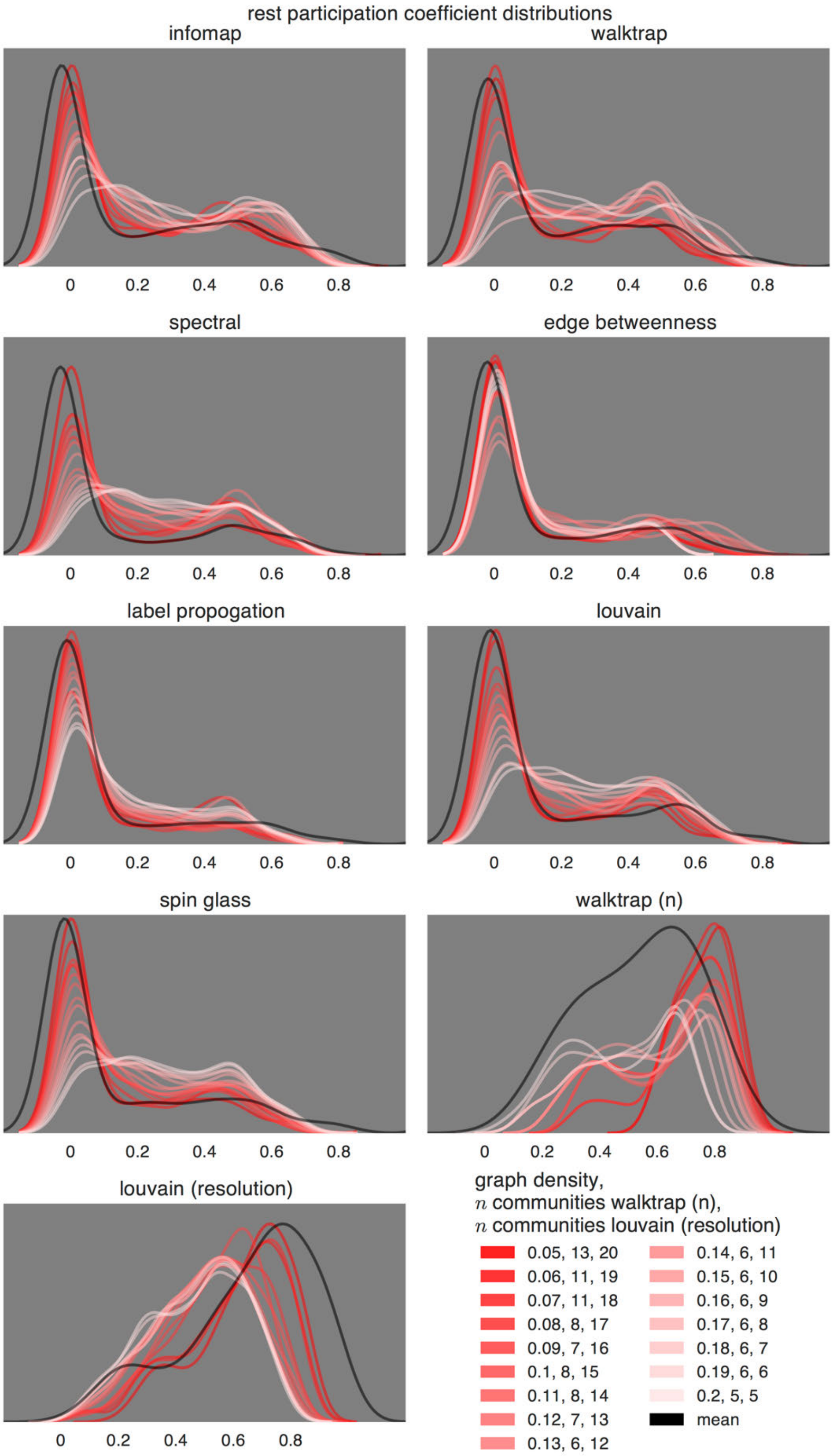

Supplementary Figure 25 I Participation coefficient distribution of the human resting state network. For this task's network and each community detection method, for each density, number of communities, or resolution, a kernel density estimate was fit to the network's participation coefficient distribution and is plotted. The "mean" is the mean kernel density estimate.

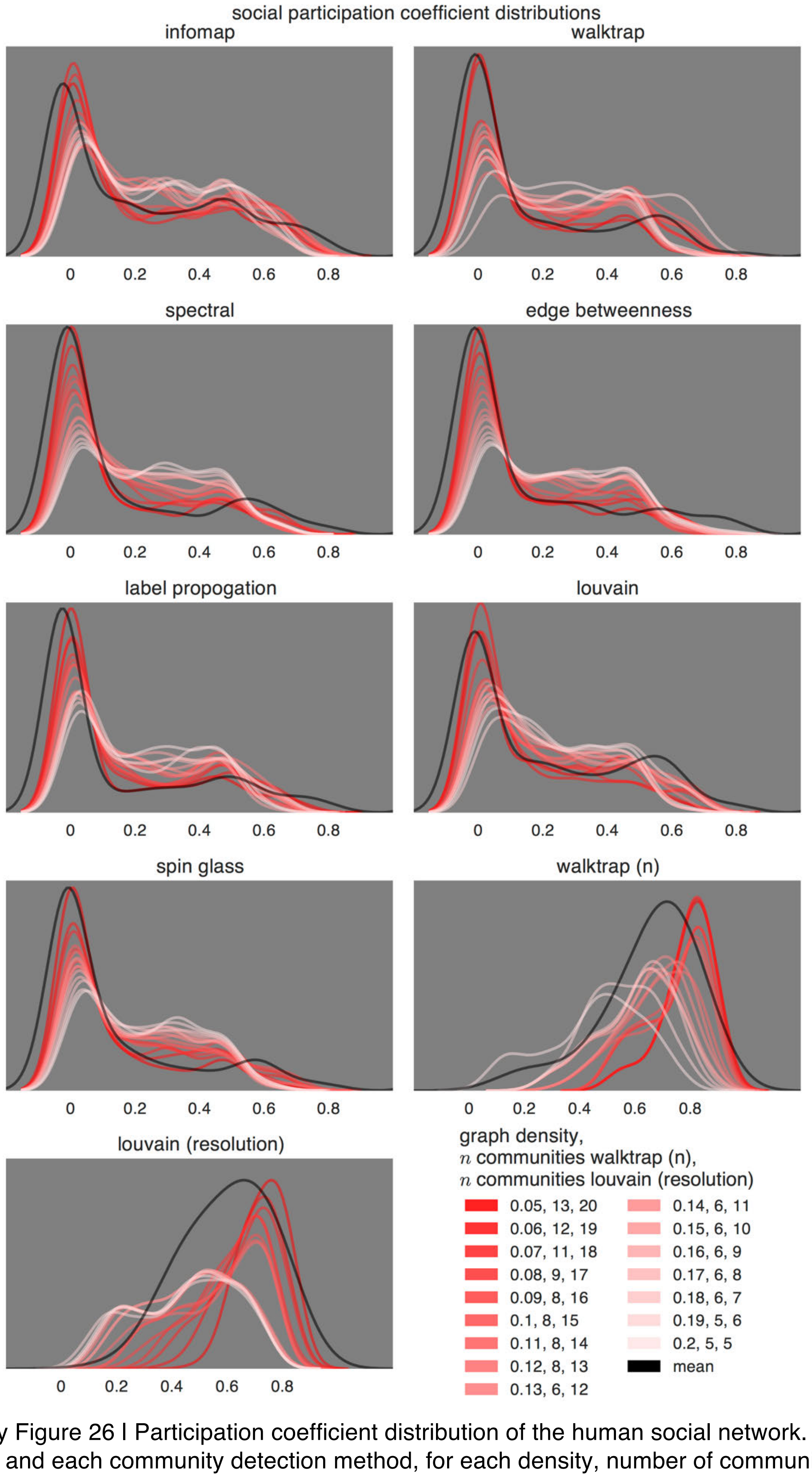

Supplementary Figure 26 I Participation coefficient distribution of the human social network. For this task's network and each community detection method, for each density, number of communities, or resolution, a kernel density estimate was fit to the network's participation coefficient distribution and is plotted. The "mean" is the mean kernel density estimate.

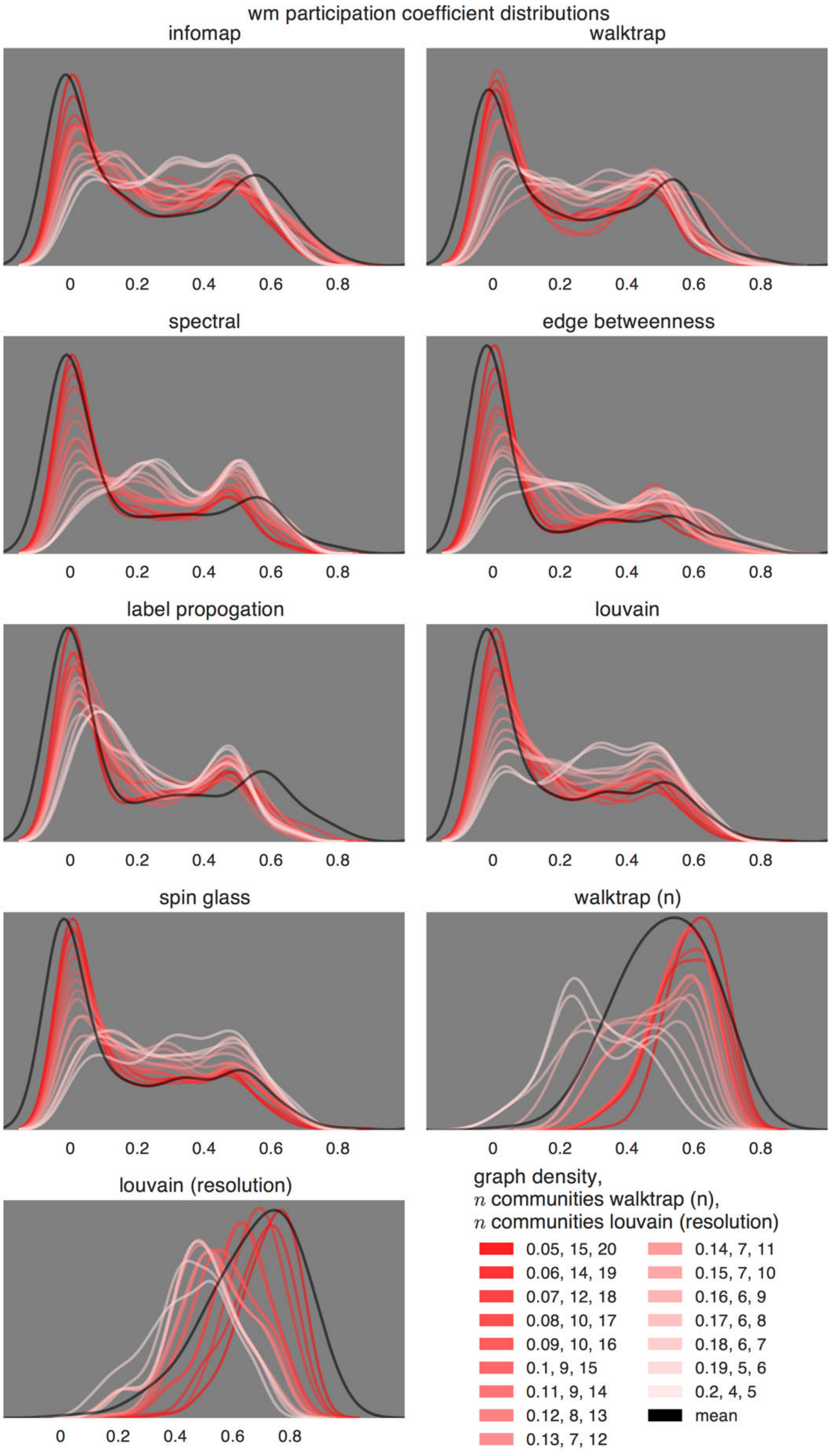

Supplementary Figure 27 I Participation coefficient distribution of the human working memory network. For this task's network and each community detection method, for each density, number of communities, or resolution, a kernel density estimate was fit to the network's participation coefficient distribution and is plotted. The "mean" is the mean kernel density estimate.

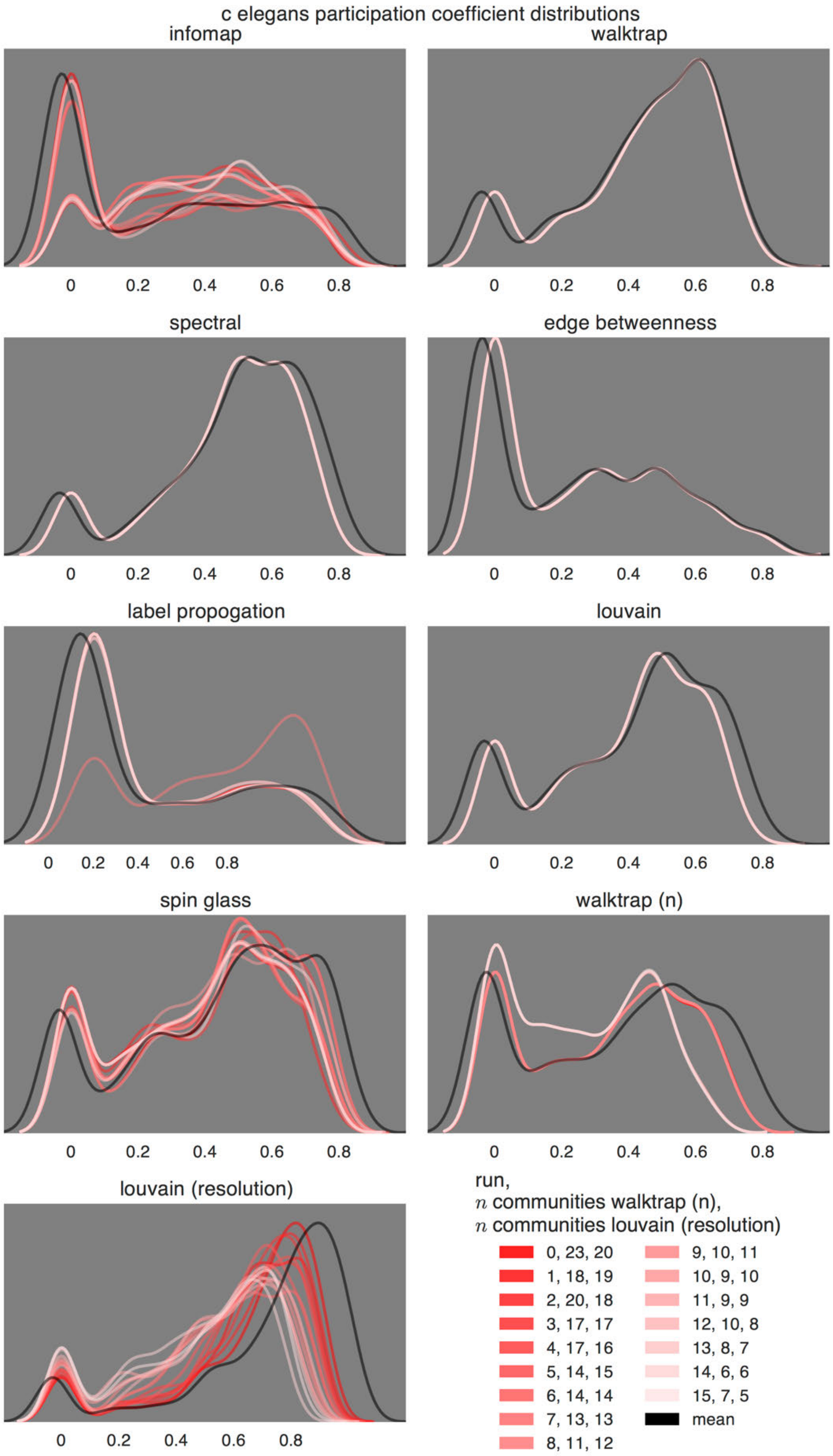

Supplementary Figure 28 I Participation coefficient distribution of the structural c elegans network. For each community detection method, for each run, number of communities, or resolution, a kernel density estimate was fit to the network's participation coefficient distribution and is plotted. The "mean" is the mean kernel density estimate.

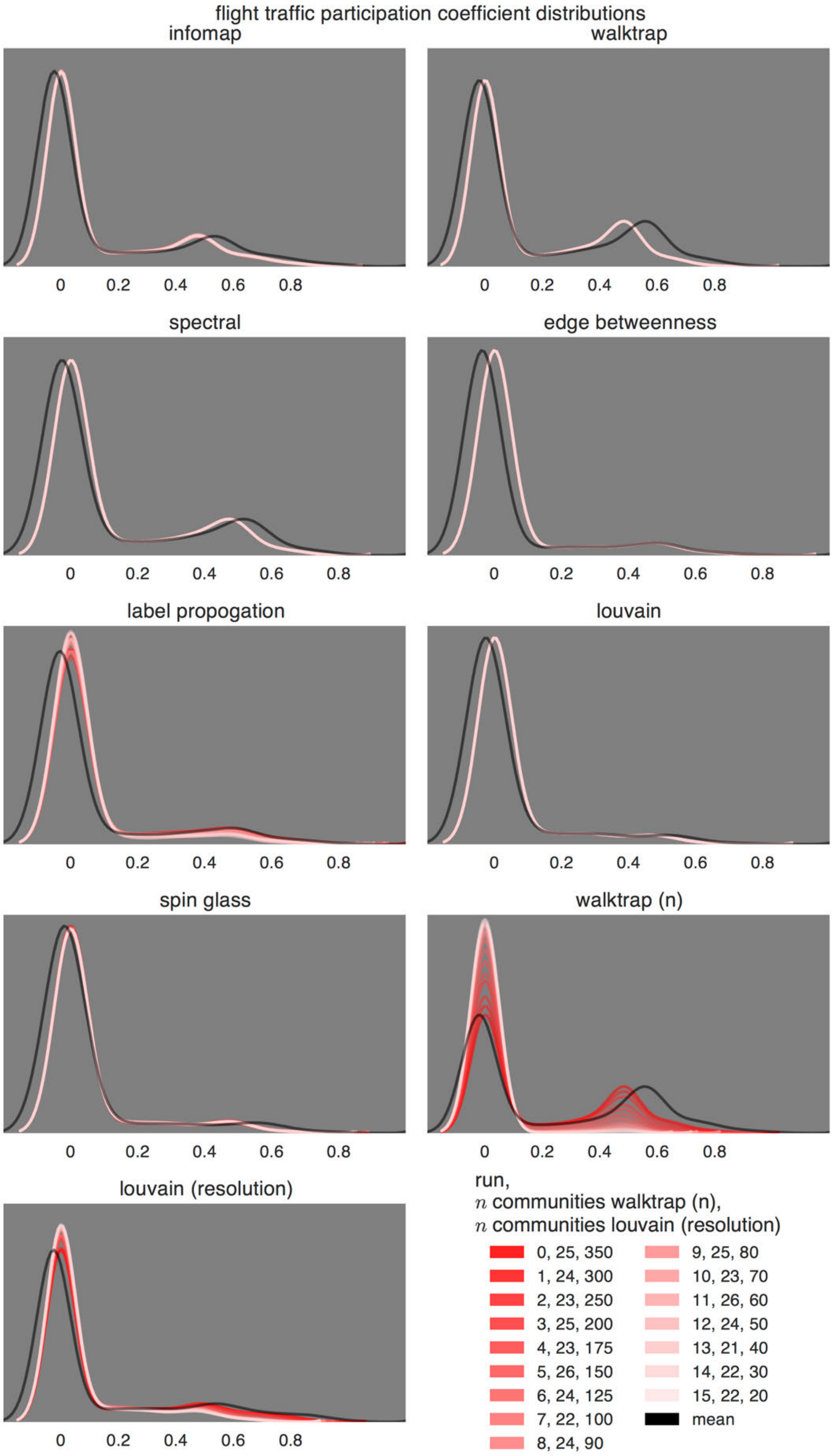

Supplementary Figure 29 I Participation coefficient distribution of the flight traffic network. For each community detection method, for each run, number of communities, or resolution, a kernel density estimate was fit to the network's participation coefficient distribution and is plotted. The "mean" is the mean kernel density estimate.

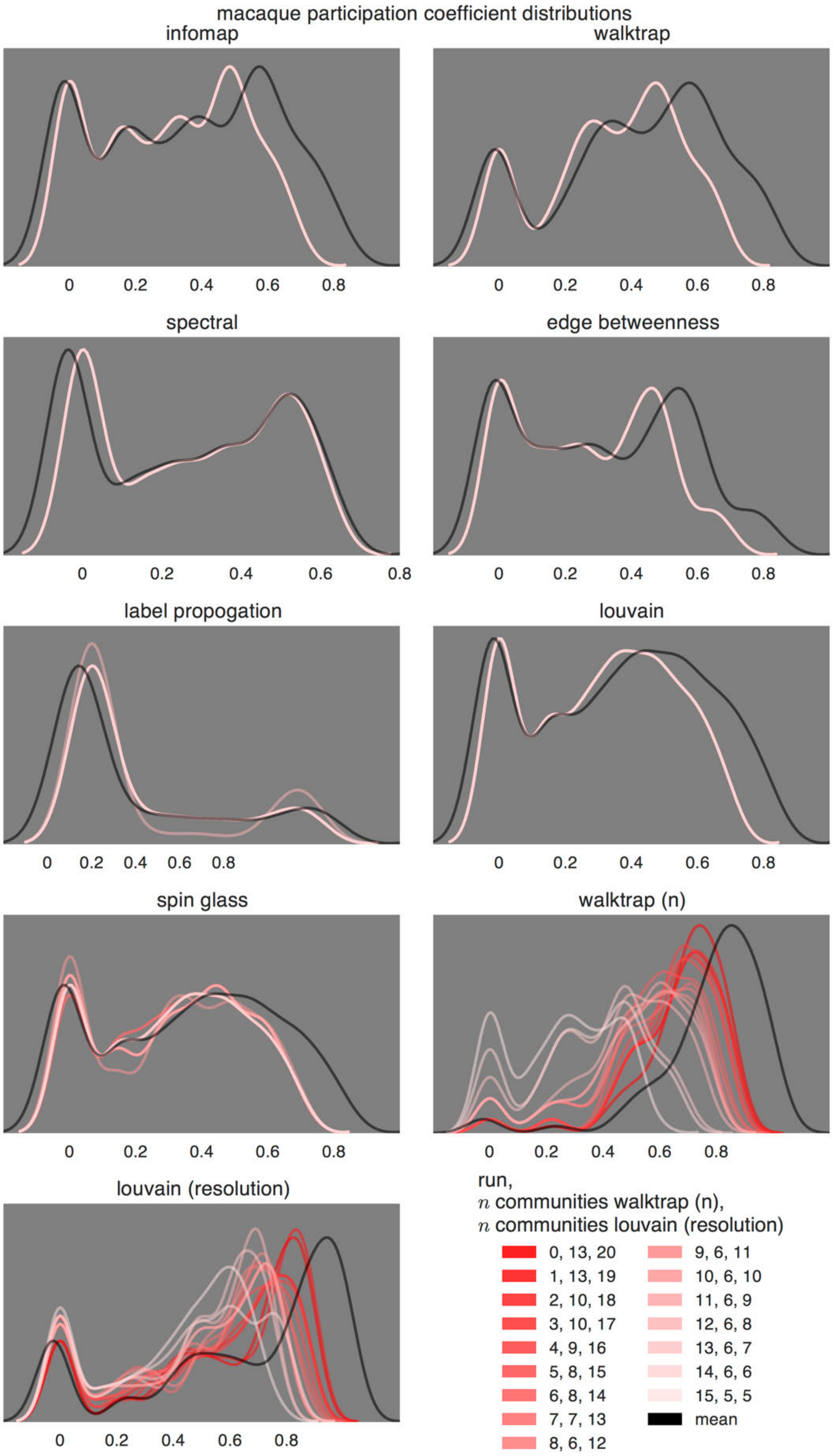

Supplementary Figure 30 I Participation coefficient distribution of the macaque network. For each community detection method, for each run, number of communities, or resolution, a kernel density estimate was fit to the network's participation coefficient distribution and is plotted. The "mean" is the mean kernel density estimate.

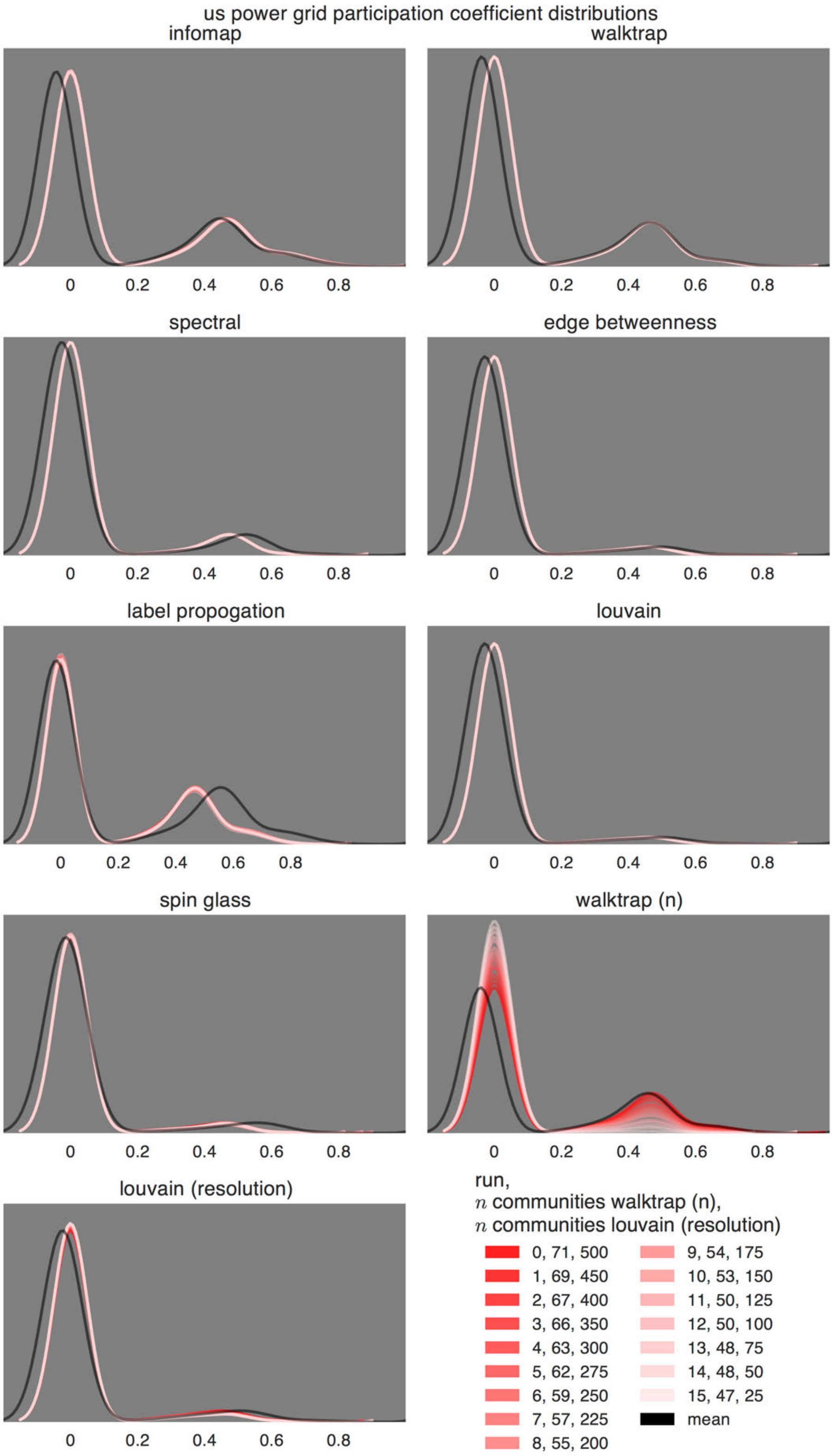

Supplemental Figure 31 I Participation coefficient distribution of the US power grid network. For each community detection method, for each run, number of communities, or resolution, a kernel density estimate was fit to the network's participation coefficient distribution and is plotted. The "mean" is the mean kernel density estimate.

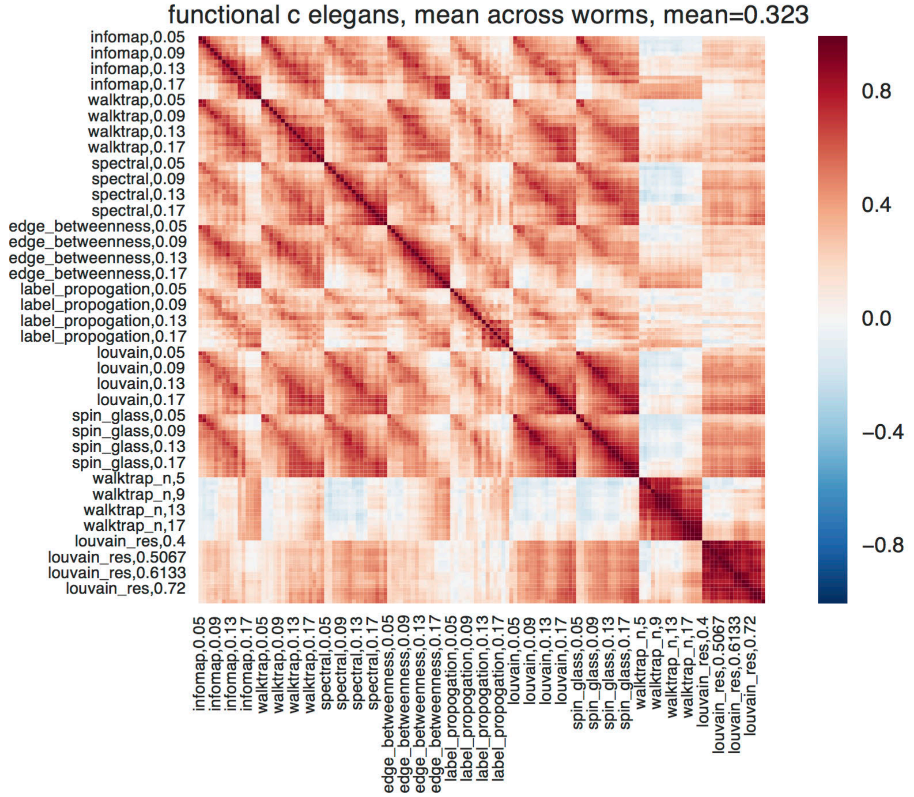

Supplemental Figure 32 | Similarity of participation coefficients in the functional c elegans. Across each community detection method, as well as across graph densities, the number of communities requested (Walktrap N), or the resolution (Louvain Resolution), the spearman correlation *r* between the participation coefficients is shown. The mean across the four worms is shown.

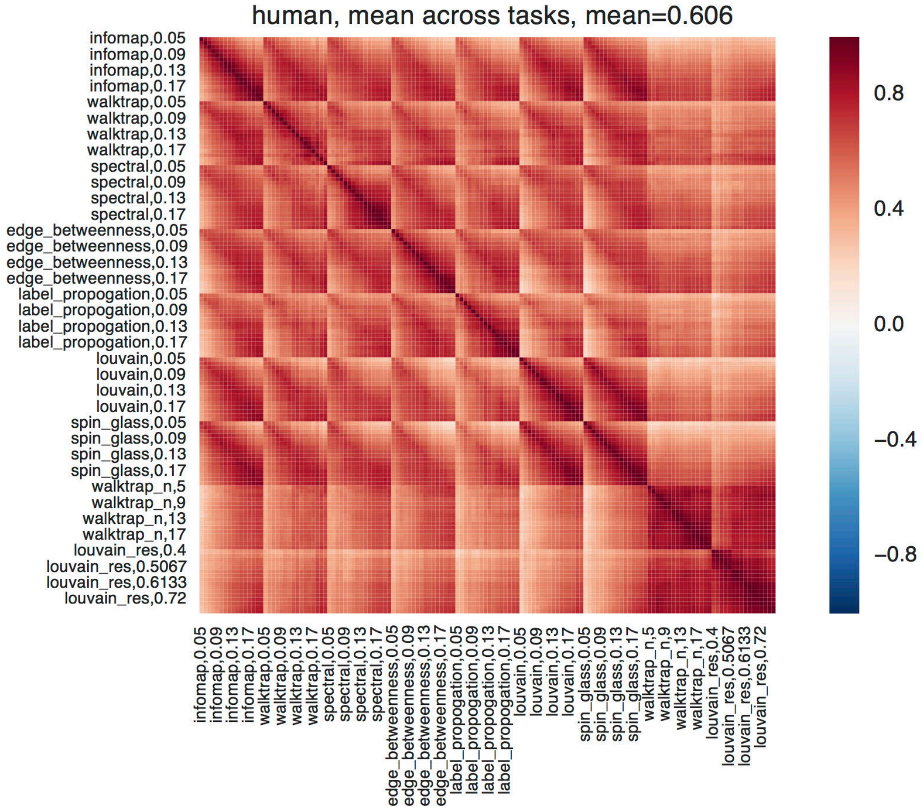

Supplemental Figure 33 l Similarity of participation coefficients in the human. Across each community detection method, as well as across graph densities, the number of communities requested (Walktrap N), or the resolution (Louvain Resolution), the spearman correlation *r* between the participation coefficients is shown. The mean across tasks shown.

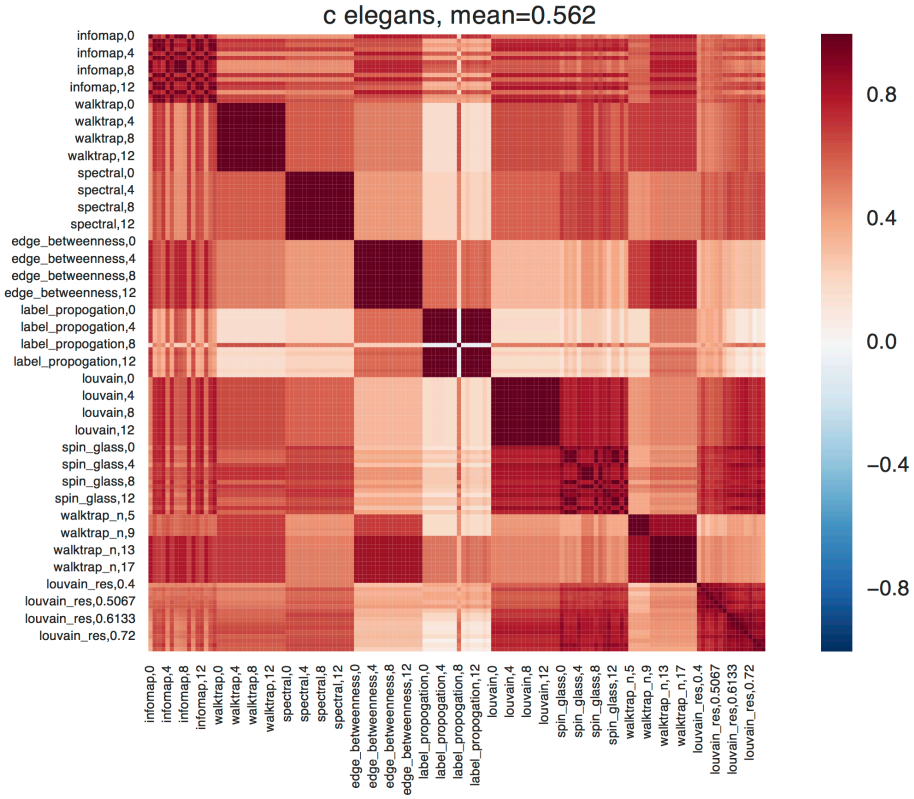

Supplemental Figure 34 l Similarity of participation coefficients in the structural c elegans. Across each community detection method, as well as across runs, the number of communities requested (Walktrap N), or the resolution (Louvain Resolution), the spearman correlation between *r* the participation coefficients is shown.

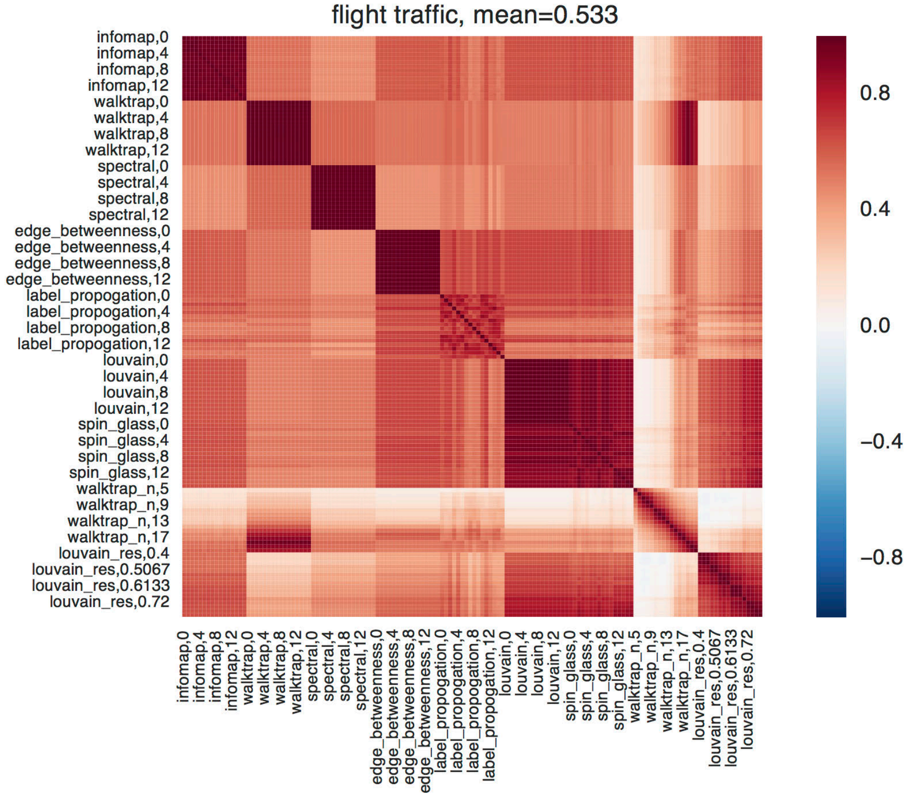

Supplemental Figure 35 I Similarity of participation coefficients in the flight traffic network. Across each community detection method, as well as across runs, the number of communities requested (Walktrap N), or the resolution (Louvain Resolution), the spearman correlation *r* between the participation coefficients is shown.

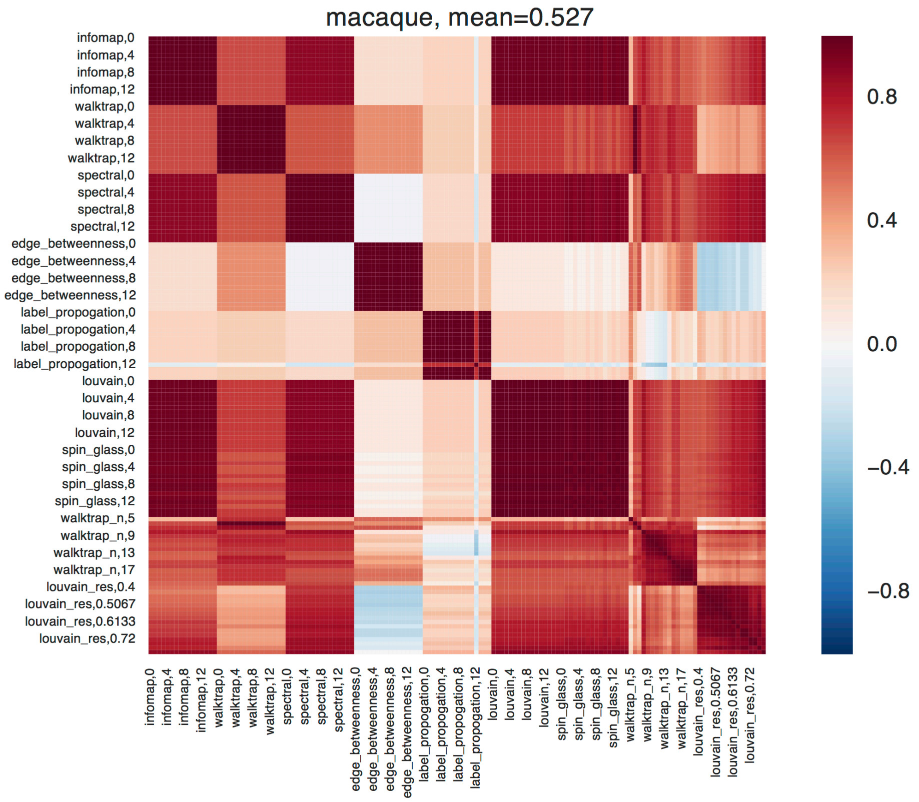

Supplemental Figure 36 l Similarity of participation coefficients in the macaque network. Across each community detection method, as well as across runs, the number of communities requested (Walktrap N), or the resolution (Louvain Resolution), the spearman correlation *r* between the participation coefficients is shown.

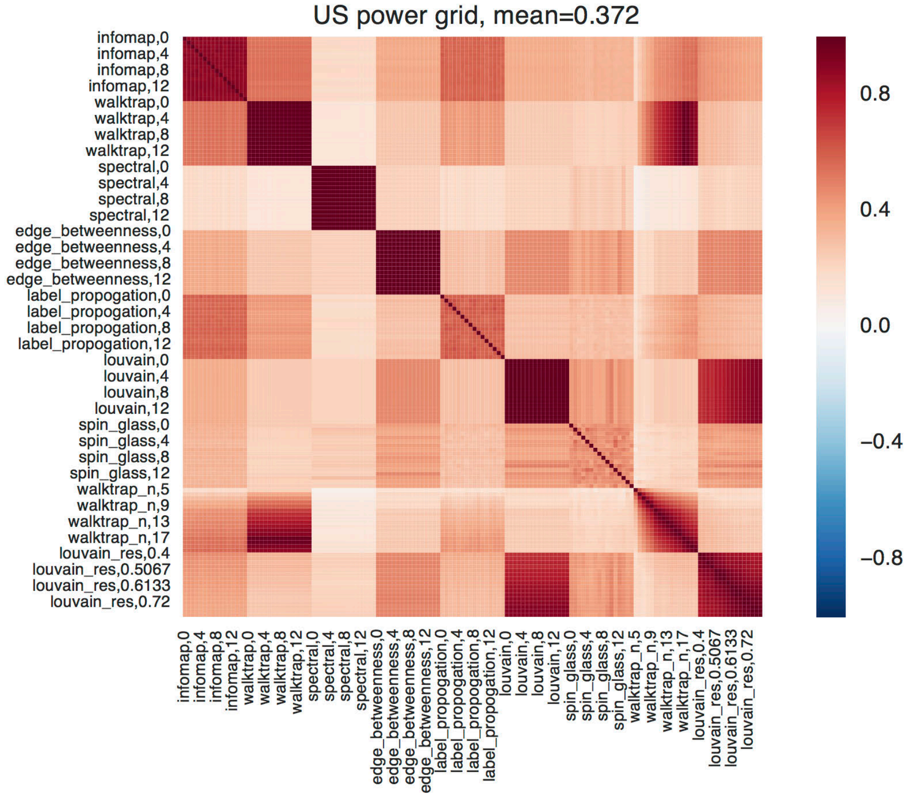

Supplemental Figure 37 I Similarity of participation coefficients in the US power grid network. Across each community detection method, as well as across runs, the number of communities requested (Walktrap N), or the resolution (Louvain Resolution), the spearman correlation *r* between the participation coefficients is shown.

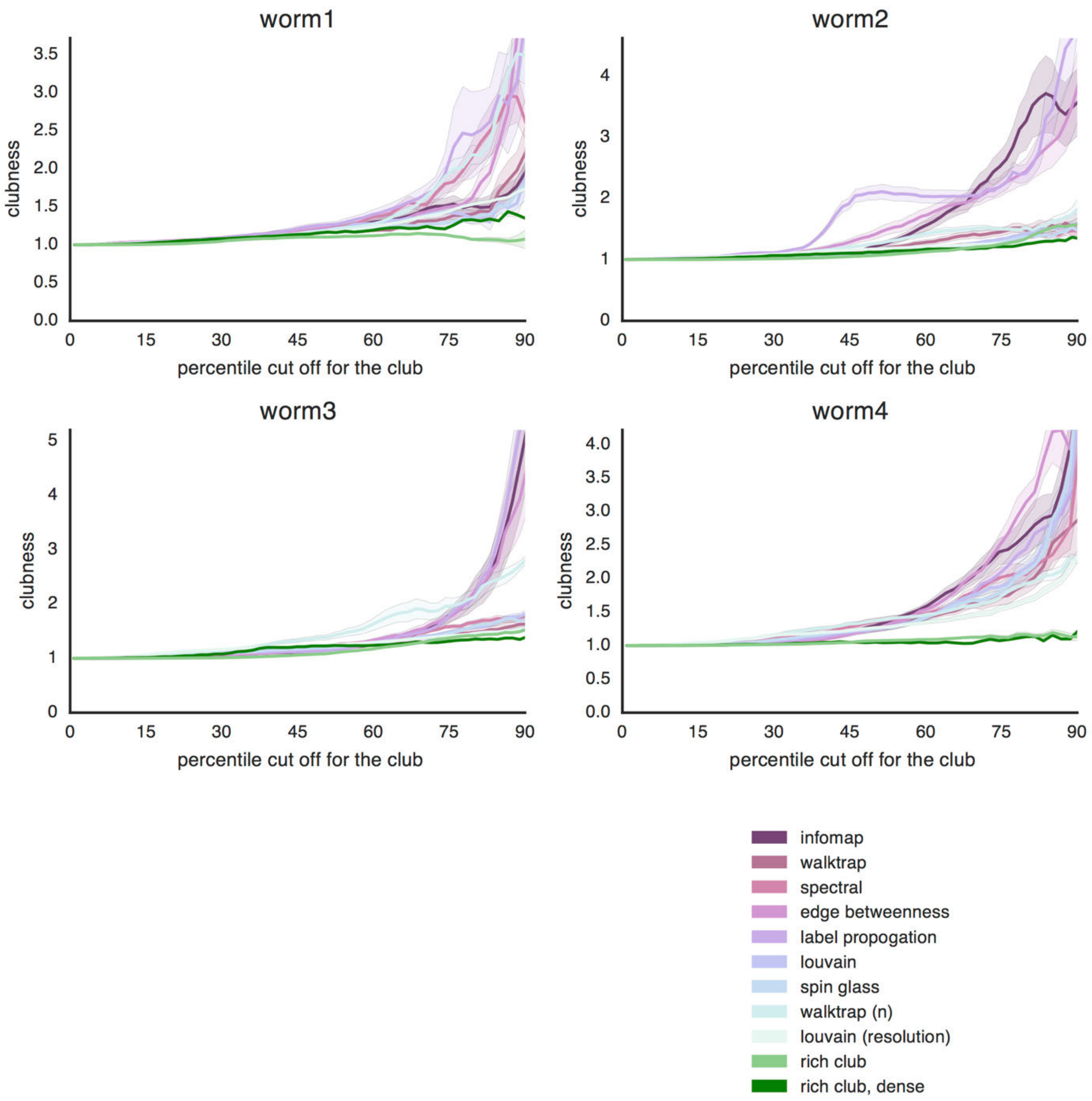

Supplementary Figure 38 I Clubness of the four c elegans functional networks. Clubness is calculated with random graphs that place the edges randomly, but retain each node's degree and sum of weights, which accounts for the contribution of edge placement, but not edge weights, to the normalized club coefficient.

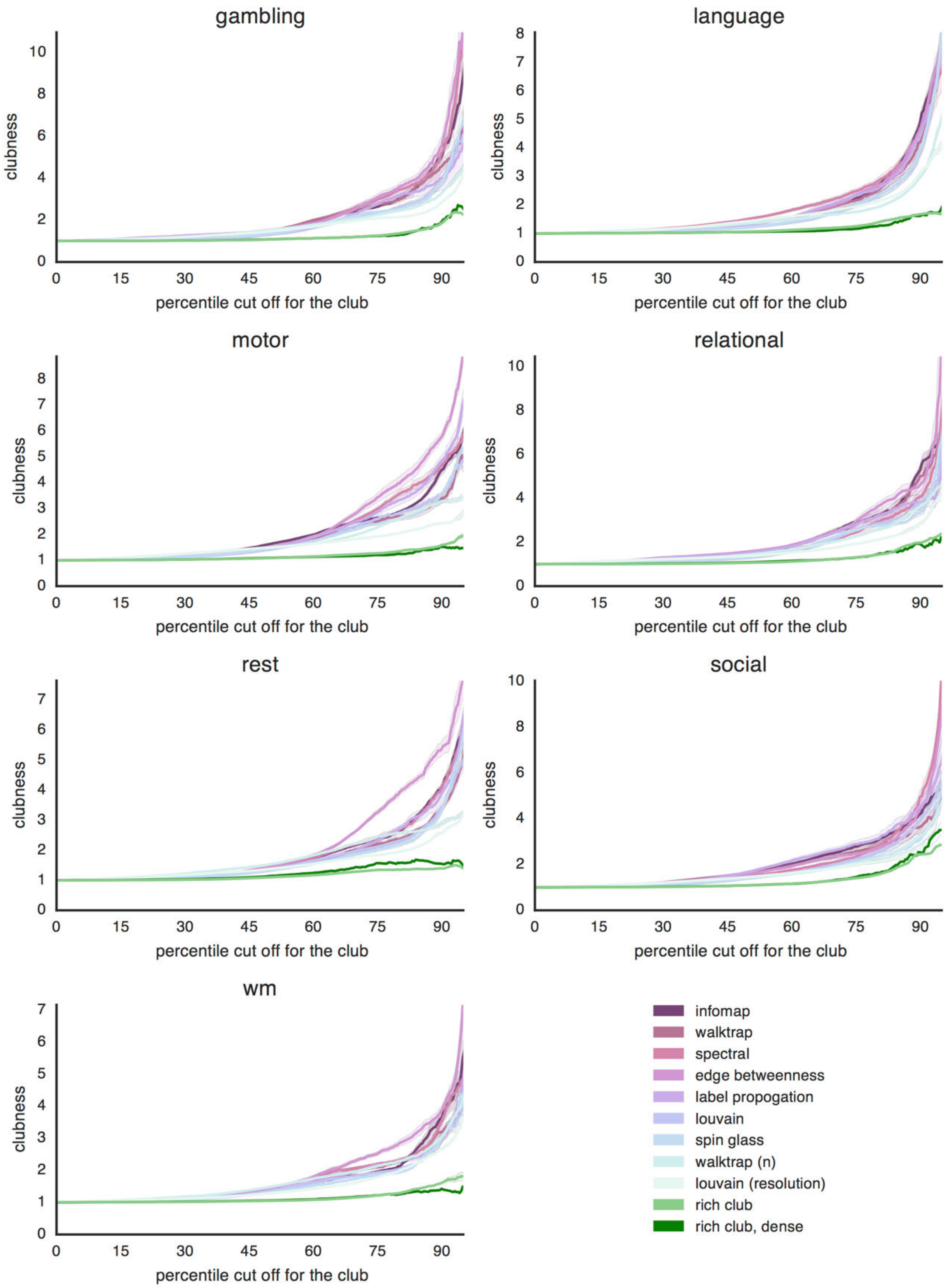

Supplementary Figure 39 I Clubness for all 7 human functional network task. Clubness is calculated with random graphs that place the edges randomly, but retain each node's degree and sum of weights, which accounts for the contribution of edge placement, but not edge weights, to the normalized club coefficient.

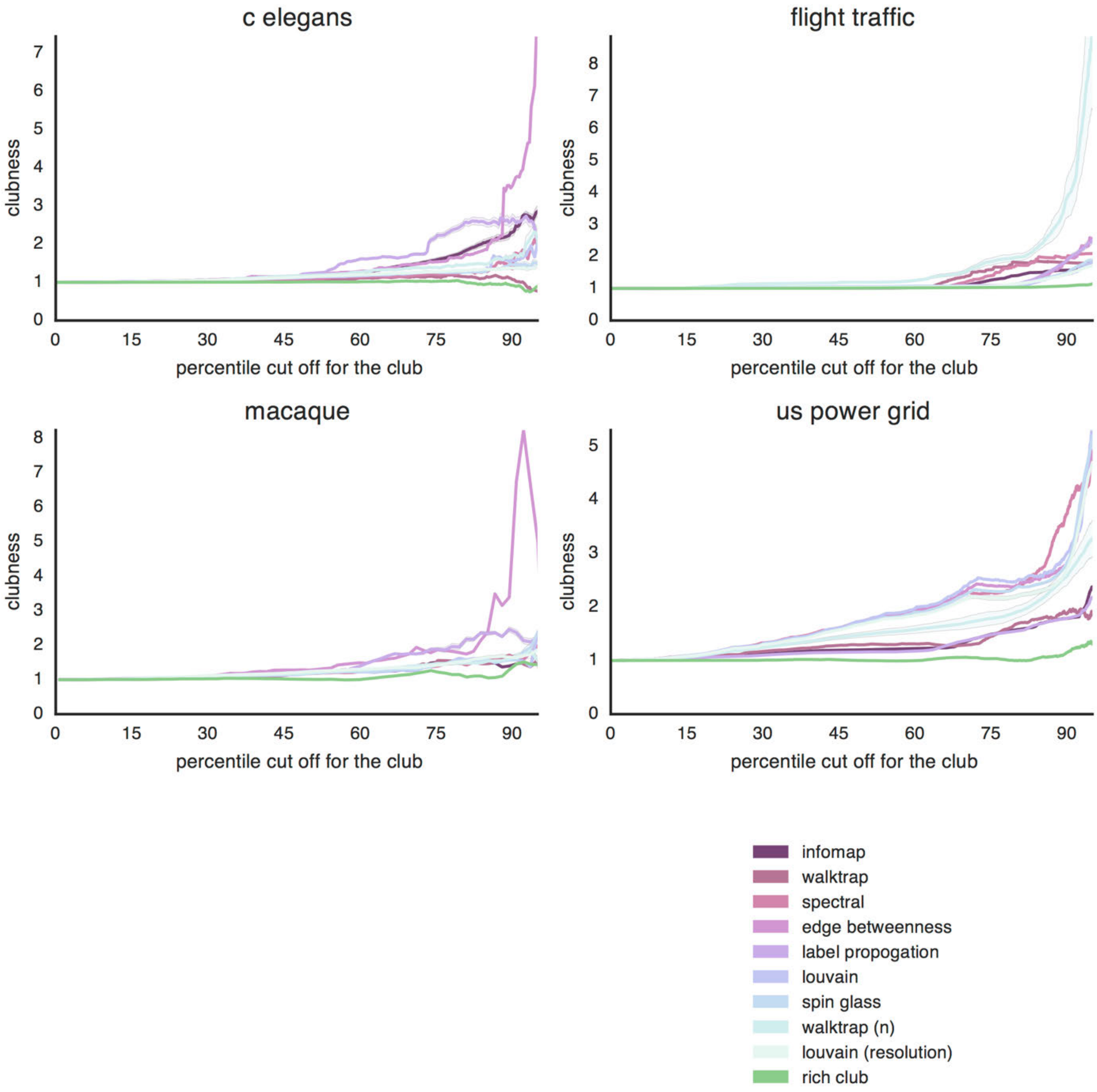

Supplementary Figure 40 l Clubness for structural networks. Clubness is calculated with random graphs that place the edges randomly, but retain each node's degree and sum of weights, which accounts for the contribution of edge placement, but not edge weights, to the normalized club coefficient.

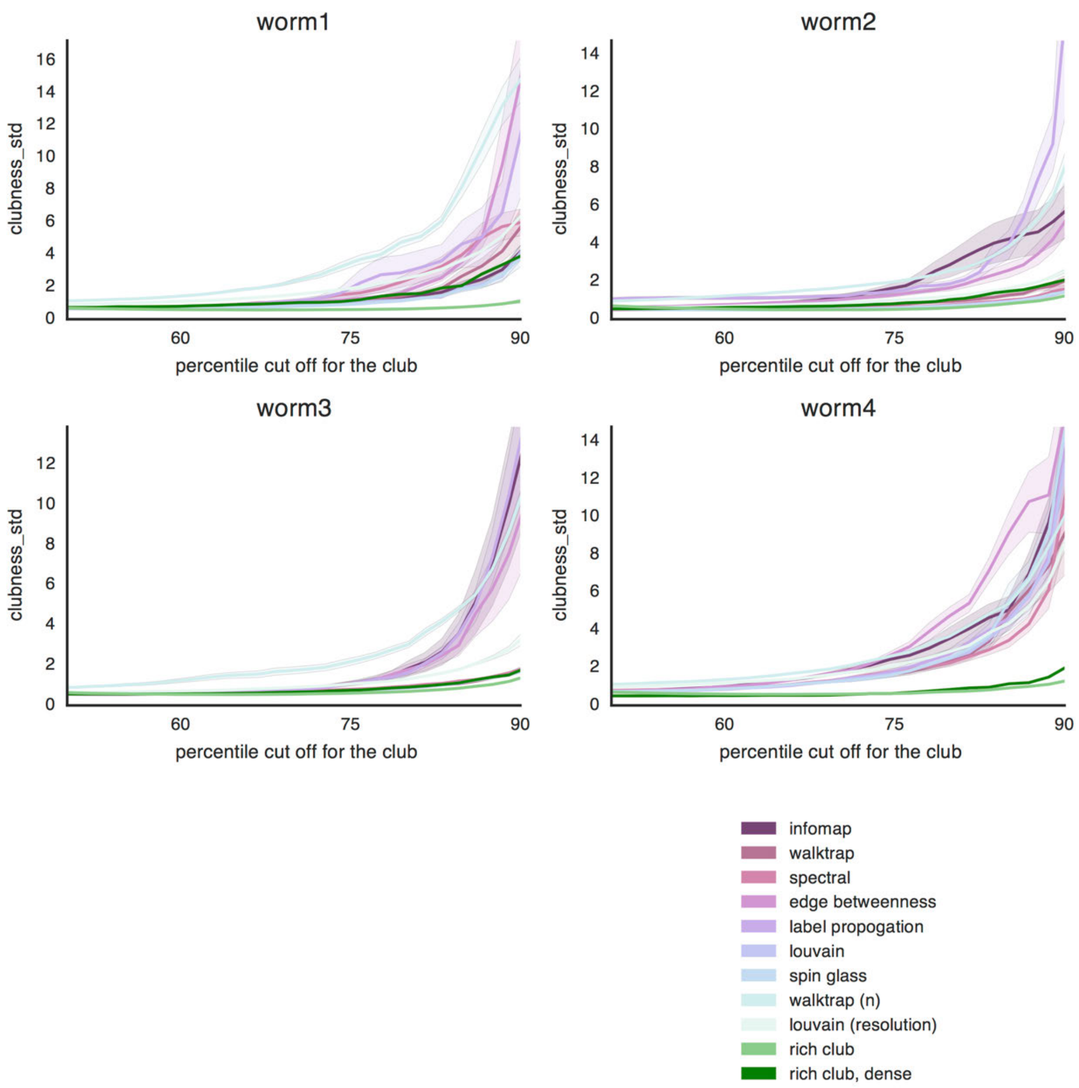

Supplementary Figure 41 I Clubness for c elegans functional networks. Here, clubness values are additionally normalized by the standard deviation of clubness values across the random graphs.

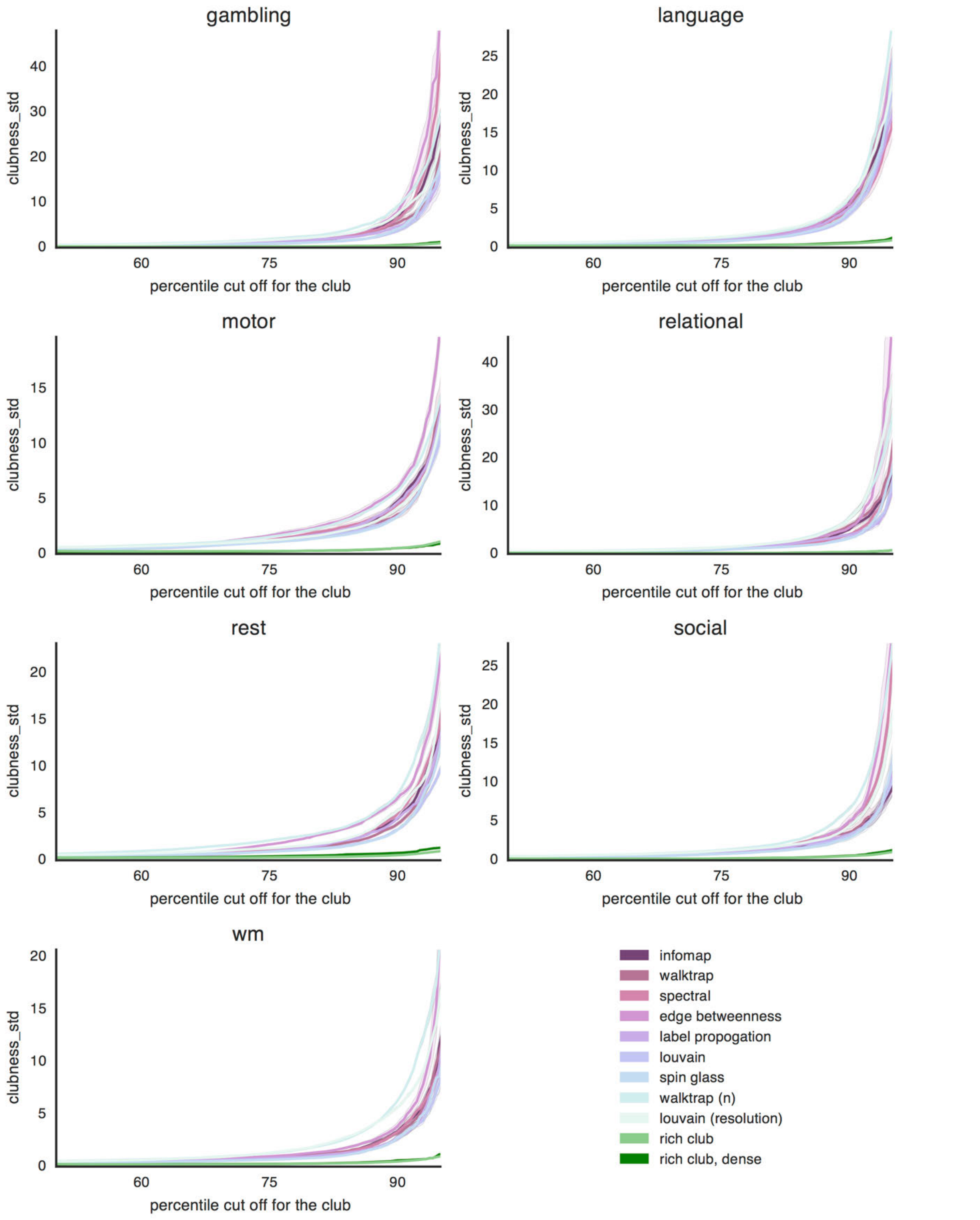

Supplementary Figure 42 | Clubness for all 7 human functional network tasks. Here, clubness values are additionally normalized by the standard deviation of clubness values across the random graphs.

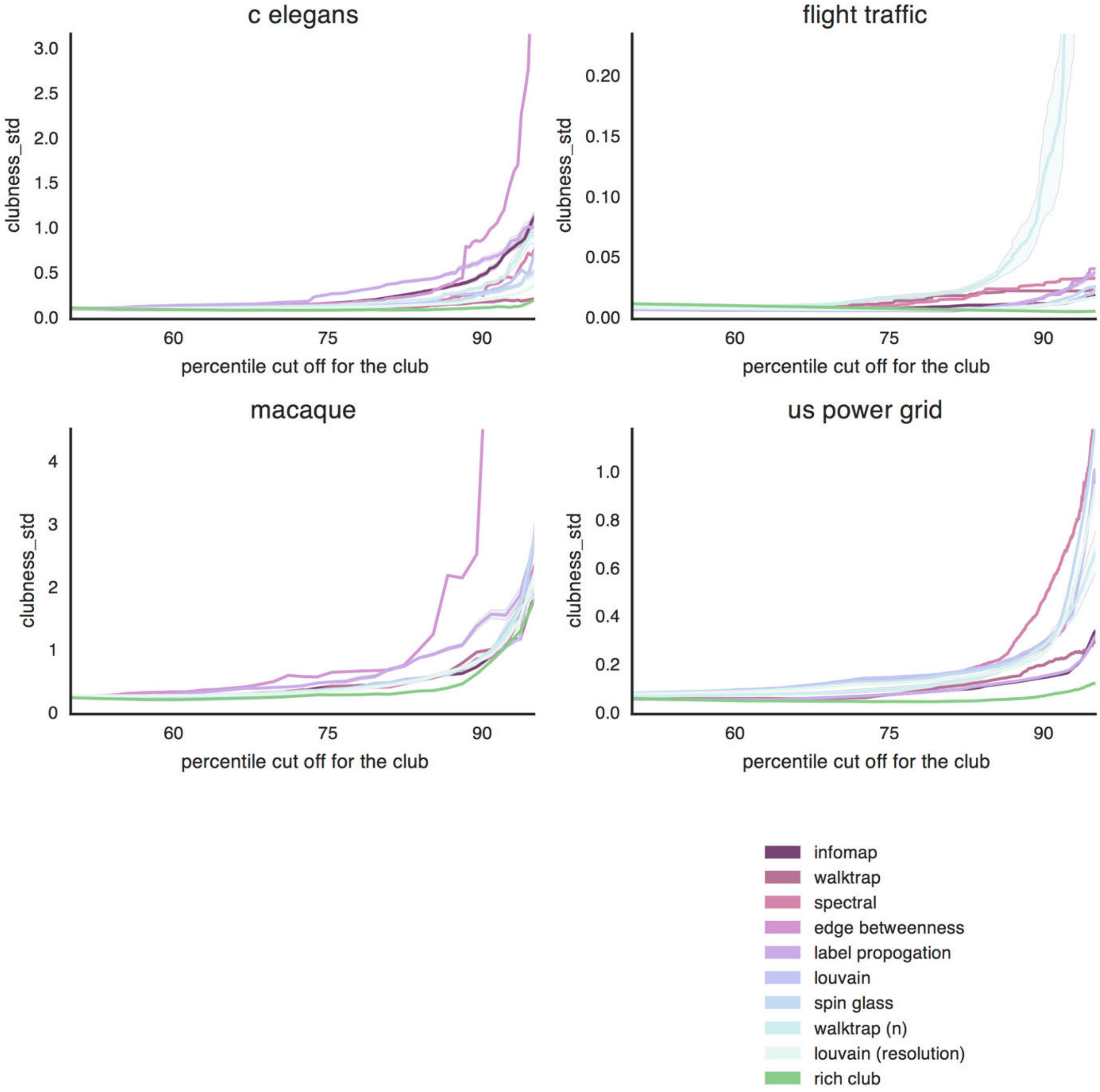

Supplementary Figure 43 I Clubness for structural networks. Here, clubness values are additionally normalized by the standard deviation of clubness values across the random graphs.

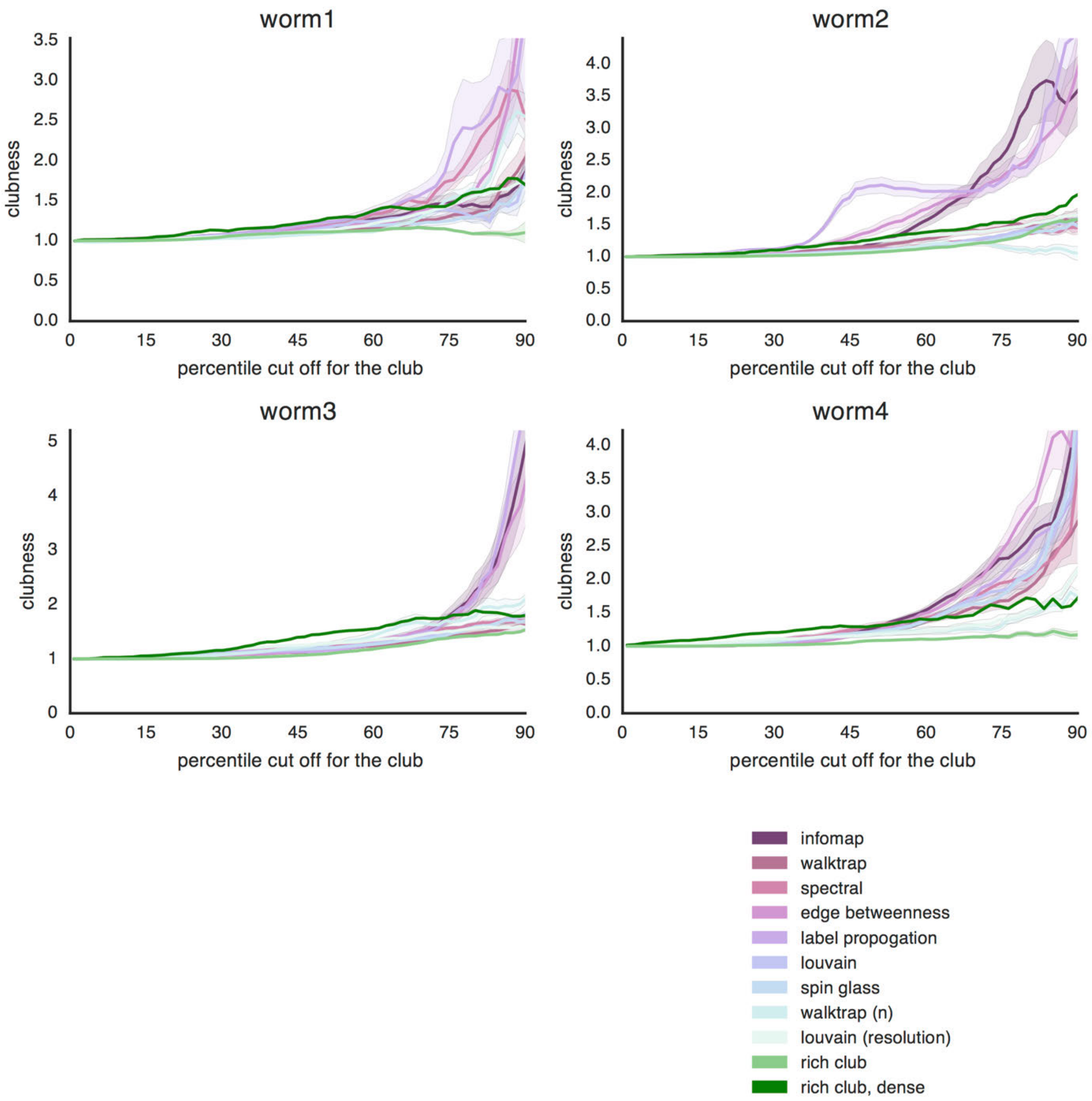

Supplementary Figure 44 I Clubness of the four c elegans functional networks. Clubness is calculated with random graphs, where all nodes maintain their degree, but the edges are randomly placed and the edge weights are shuffled between nodes with the same degree, which accounts for the contribution of both edge placement and edge weights to the normalized club coefficient.

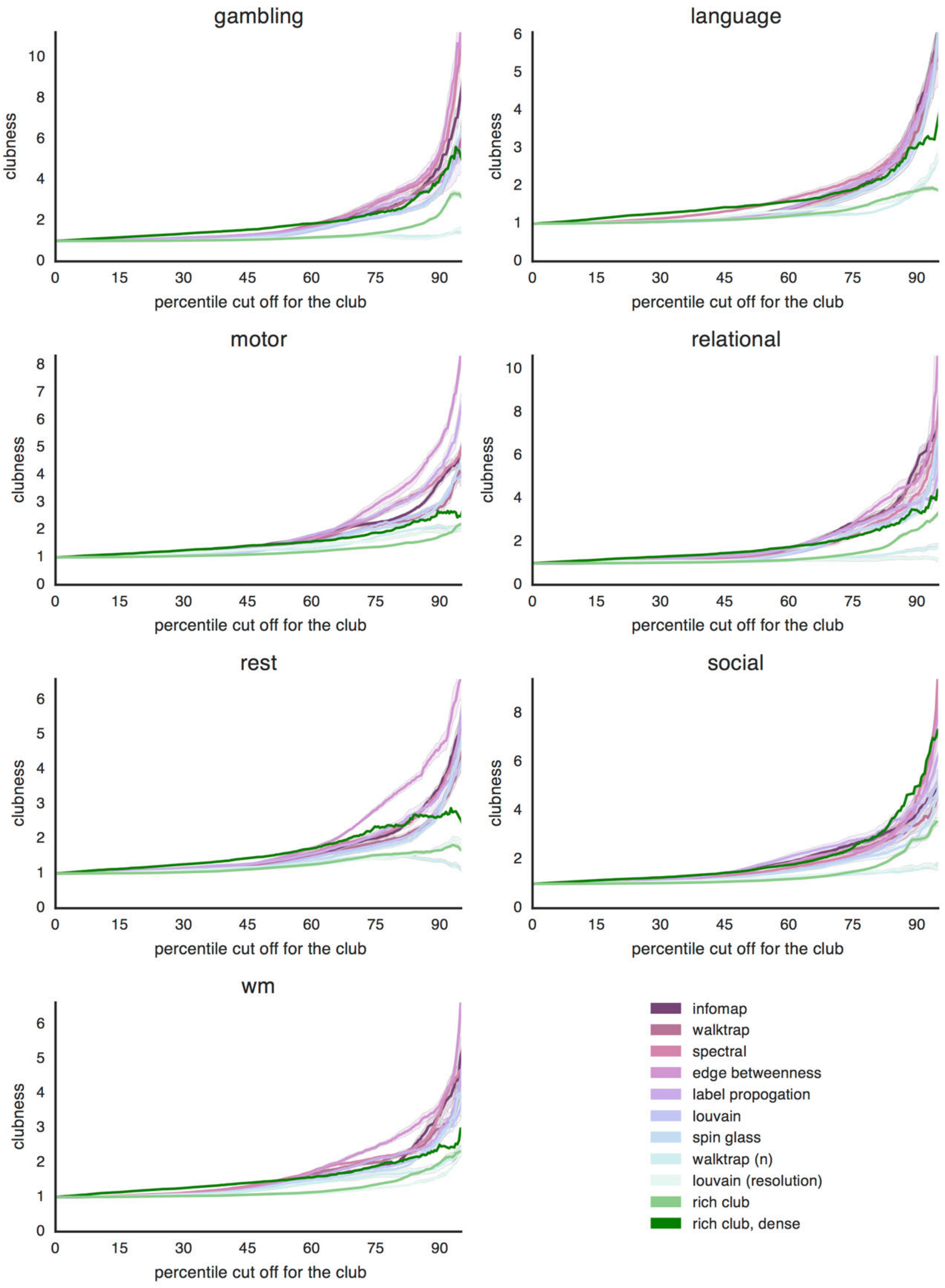

Supplementary Figure 45 | Clubness for all 7 human functional network states. Clubness is calculated with random graphs, where all nodes maintain their degree, but the edges are randomly placed and the edge weights are shuffled between nodes with the same degree, which accounts for the contribution of both edge placement and edge weights to the normalized club coefficient.

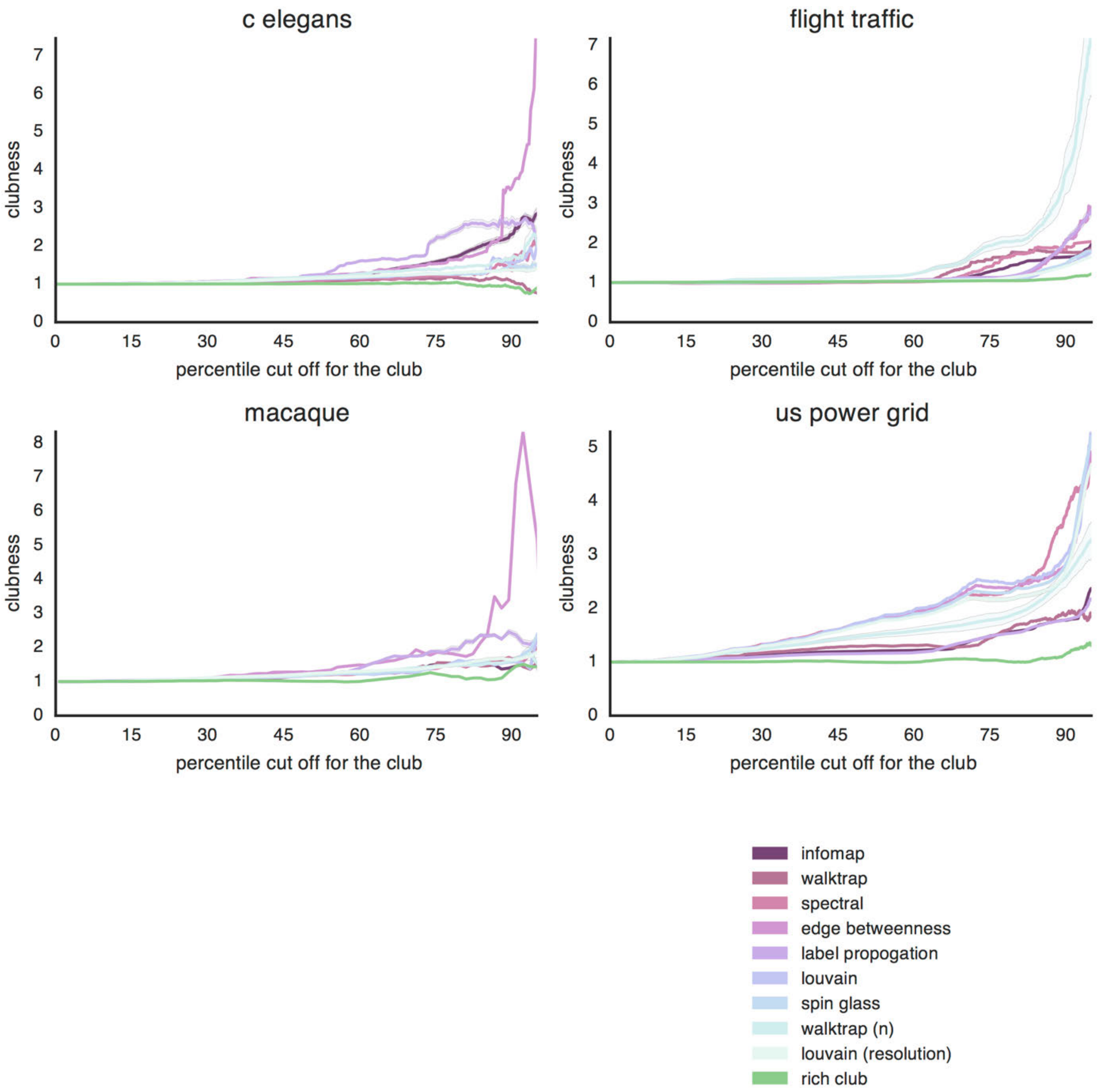

Supplementary Figure 46 I Clubness for structural networks. Clubness is calculated with random graphs, where all nodes maintain their degree, but the edges are randomly placed and the edge weights are shuffled between nodes with the same degree, which accounts for the contribution of both edge placement and edge weights to the normalized club coefficient. Here, only the flight traffic network clubness values vary from the original calculation, as this was the only structural network that was weighted).

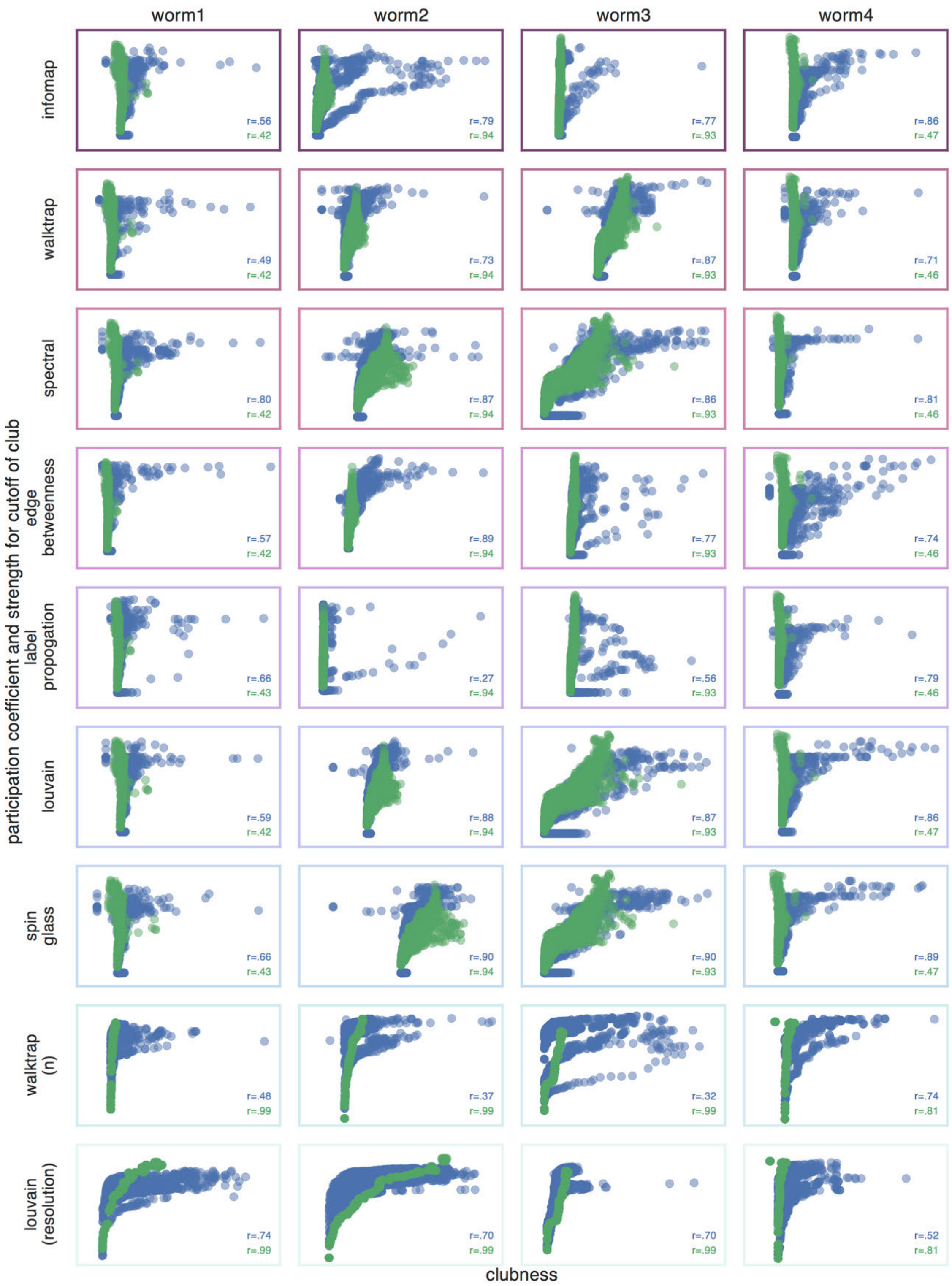

Supplementary Figure 47 I Clubness and the minimum values of club members in the functional c elegans. For each worm, for each community detection method, across densities, resolution (Louvain), or number of commuties (walktrap N), the correlation between (*y*) the minimum strength or participation coefficient value in the club at that rank and (*x*) the club's clubness at that rank.

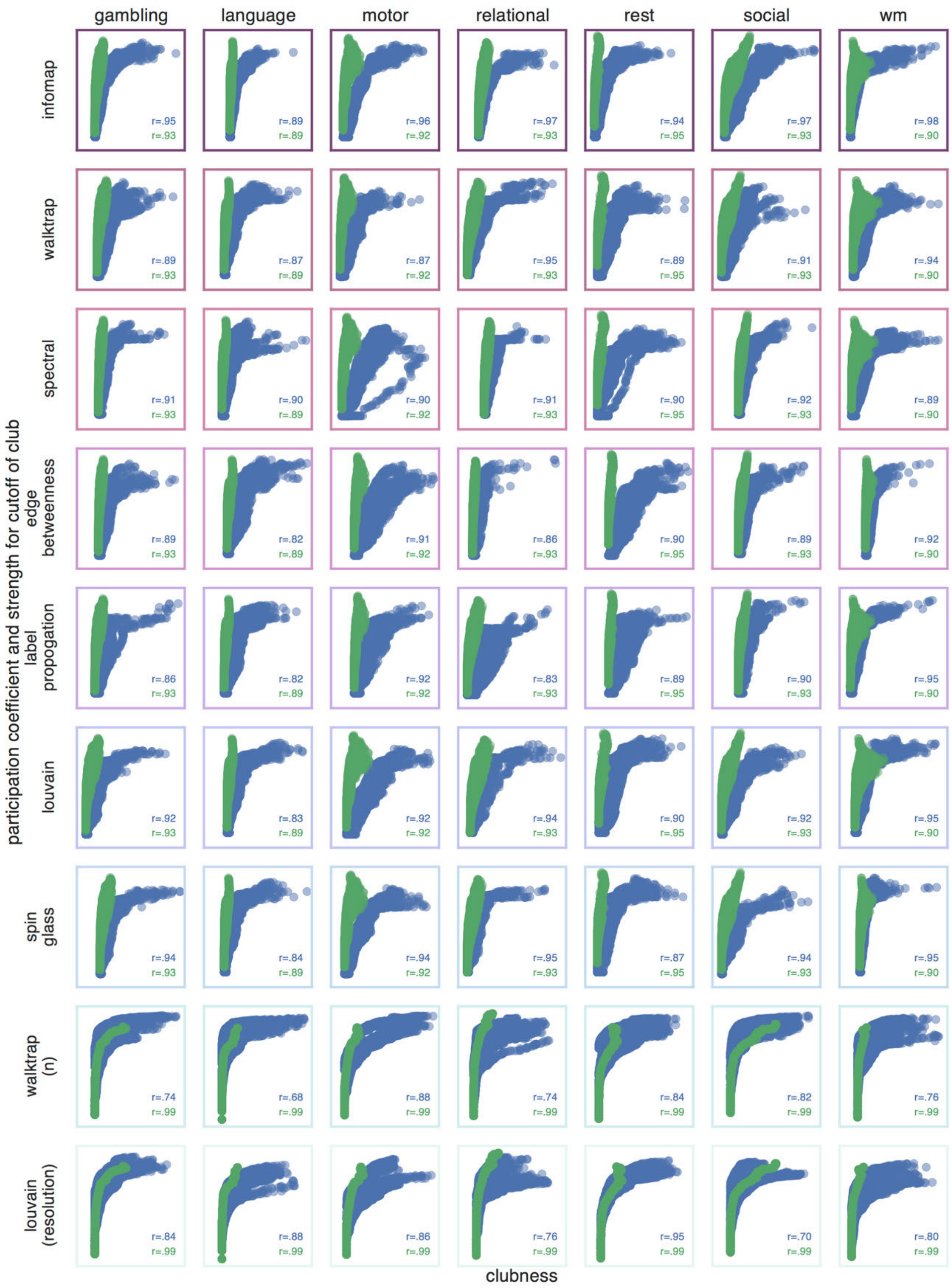

Supplementary Figure 48 I Clubness and the minimum values of club members in the human networks. For each task, for each community detection method, across densities, resolution (Louvain), or number of commuties (walktrap N), the correlation between (*y*) the minimum strength or participation coefficient value in the club at that rank and (*x*) the club's clubness at that rank.

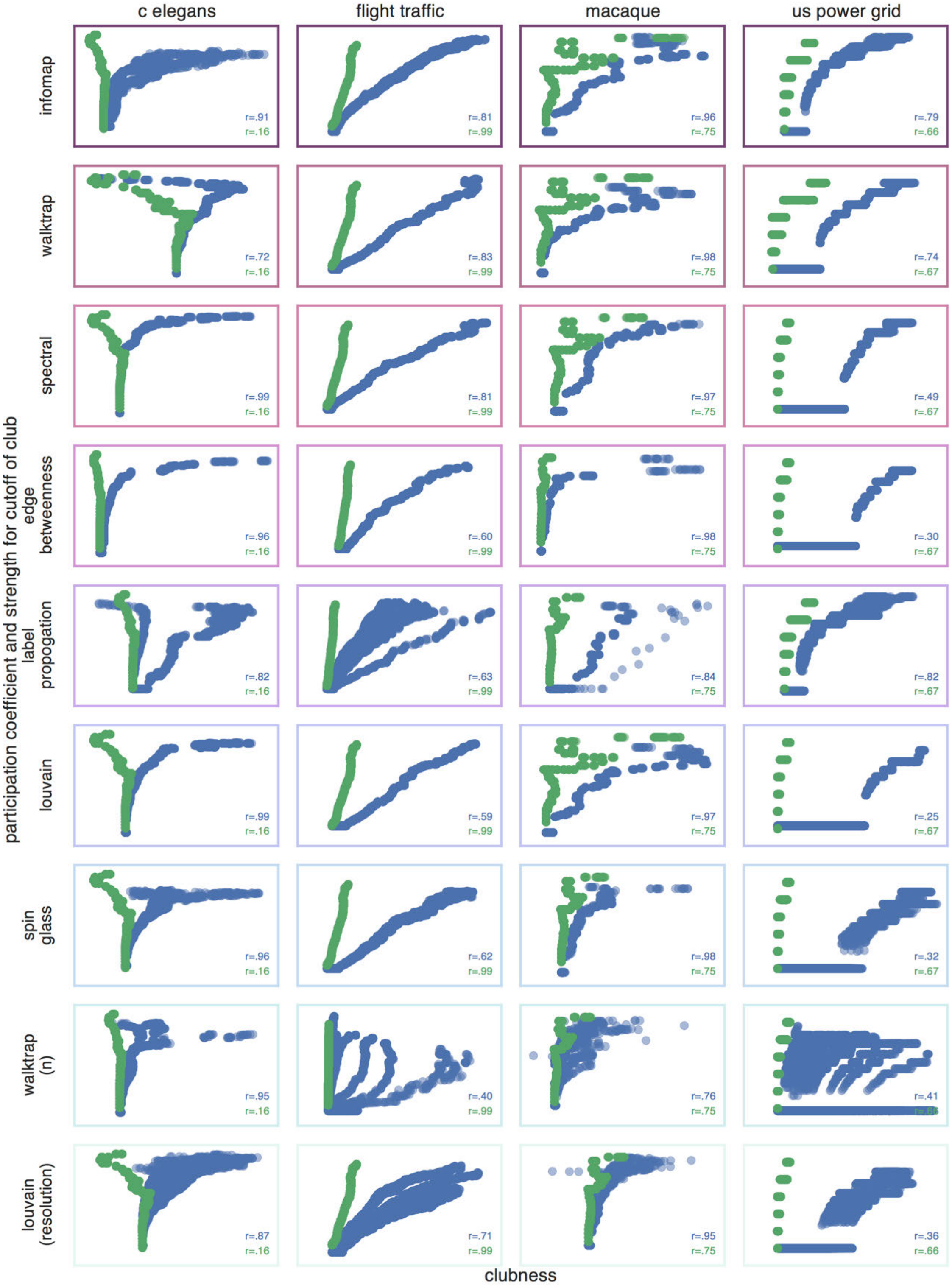

Supplementary Figure 49 I Clubness and the minimum values of club members in the structural networks. For each community detection method, across runs, resolution (Louvain), or number of commuties (walktrap N), the correlation between (*y*) the minimum strength or participation coefficient value in the club at that rank and (*x*) the club's clubness at that rank.

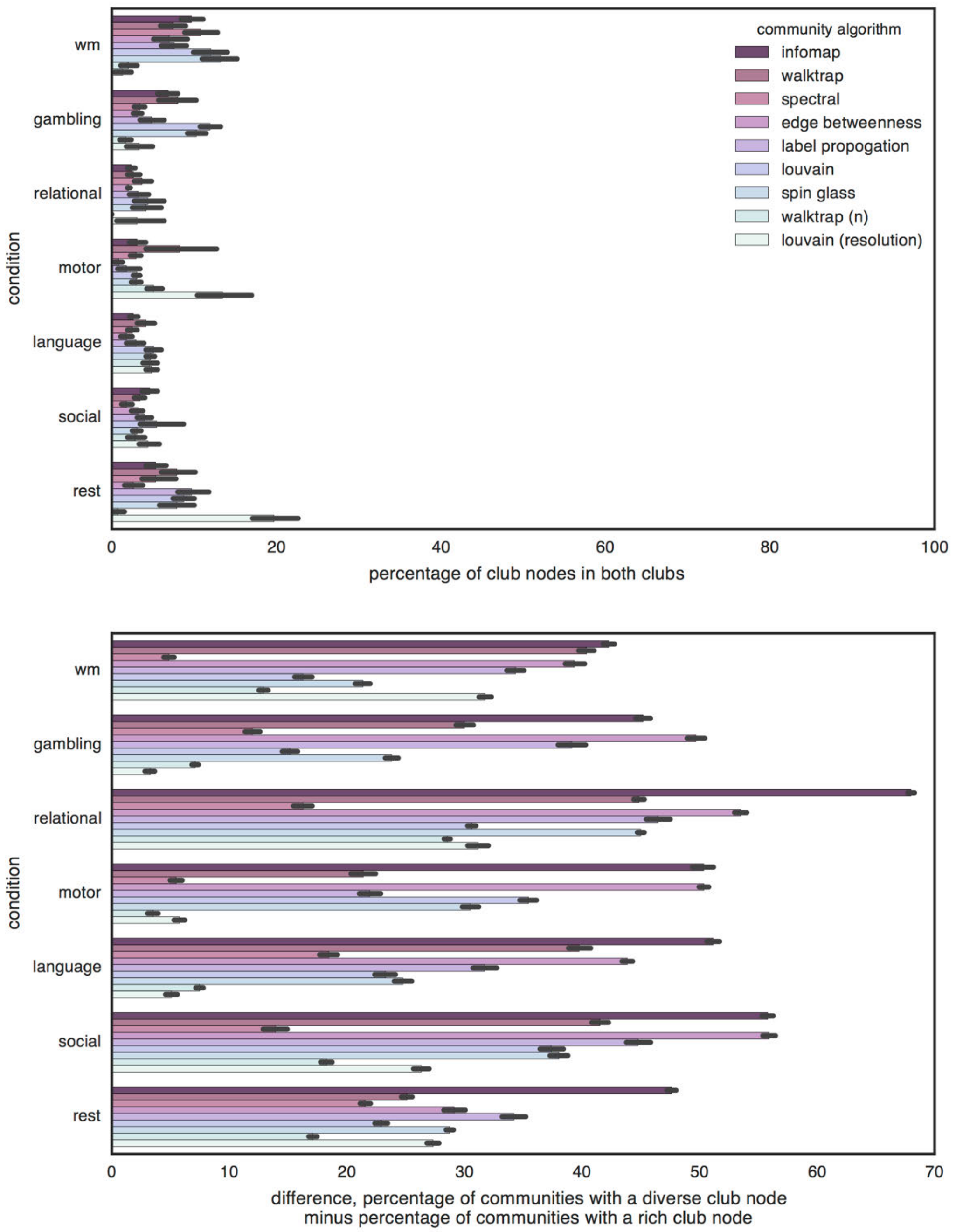

Supplementary Figure 50 I Community and club properties in human networks. Top, the percentage of nodes that are members of both clubs. 100 means the clubs are identical, and 0 means there are no nodes that are in both clubs. Bottom, the percentage of how many communities contain a diverse club node minus the percentage of how many communities contain a rich club node. Values greater than 0 mean that the diverse club spans more communities.

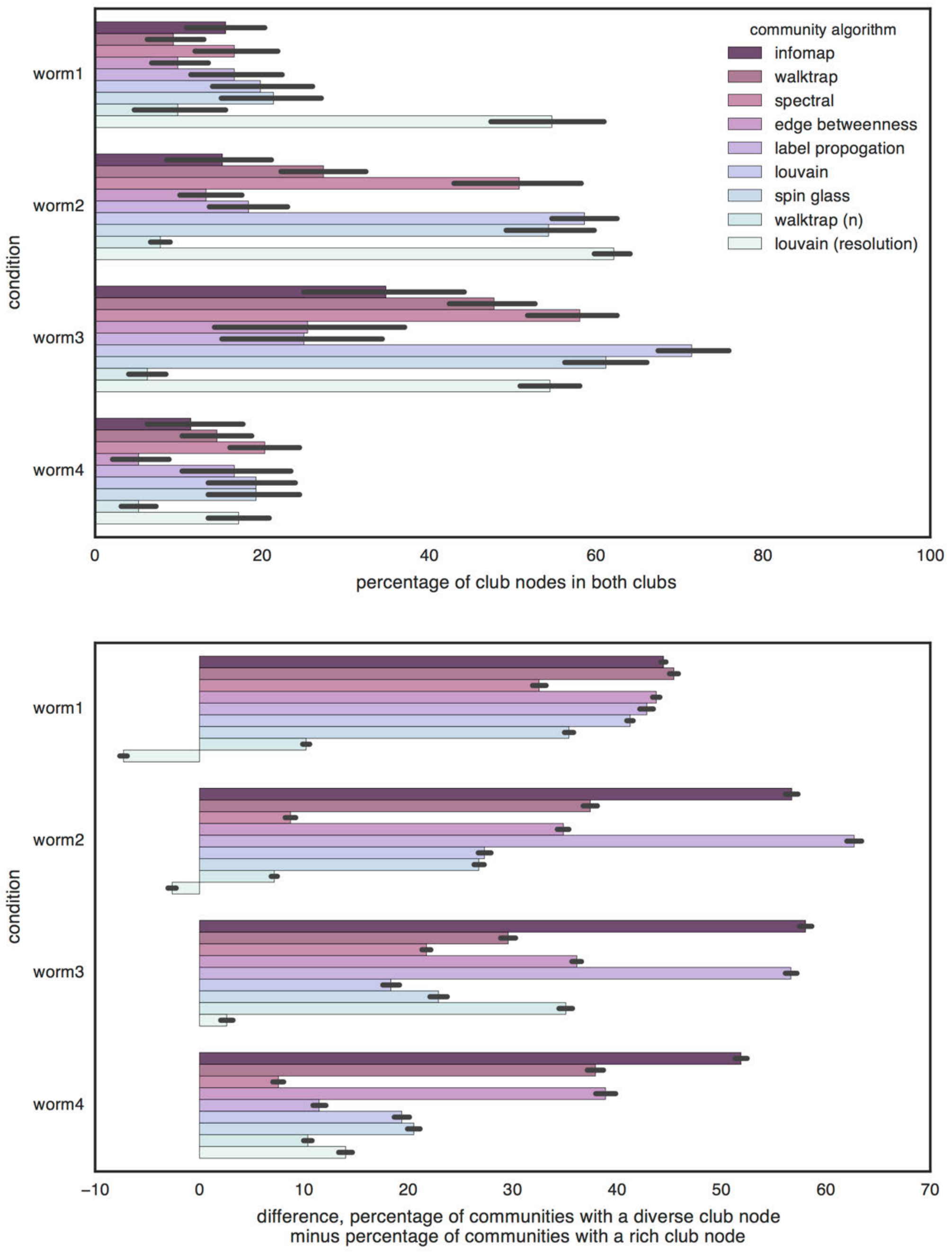

Supplementary Figure 51 I Community and club properties in functional c elegans networks. Top, the percentage of nodes that are members of both clubs. 100 means the clubs are identical, and 0 means there are no nodes that are in both clubs. Bottom, the percentage of how many communities contain a diverse club node minus the percentage of how many communities contain a rich club node. Values greater than 0 mean that the diverse club spans more communities.

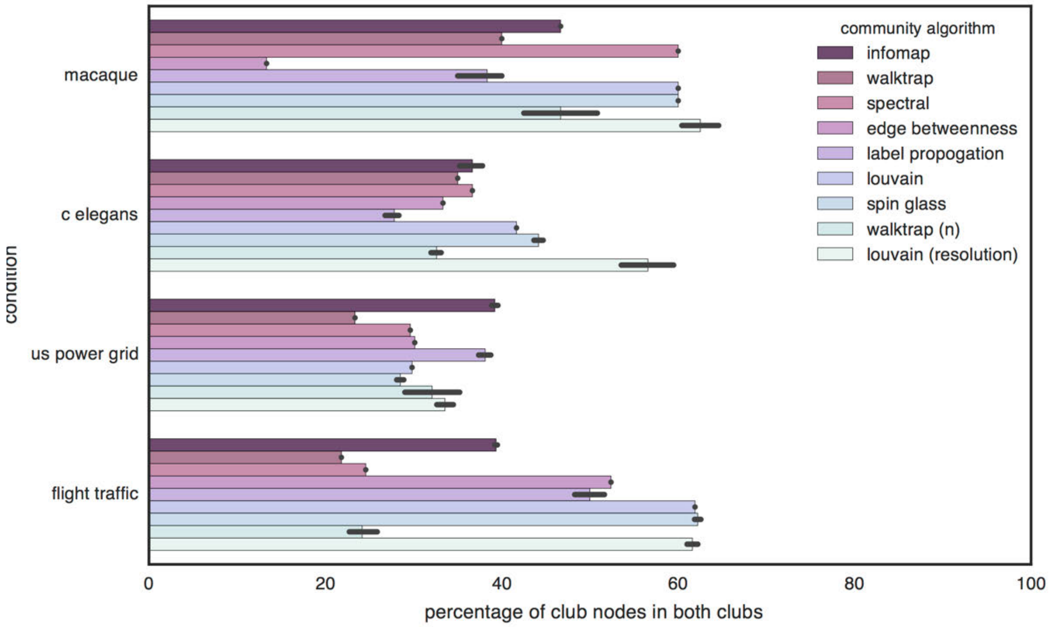

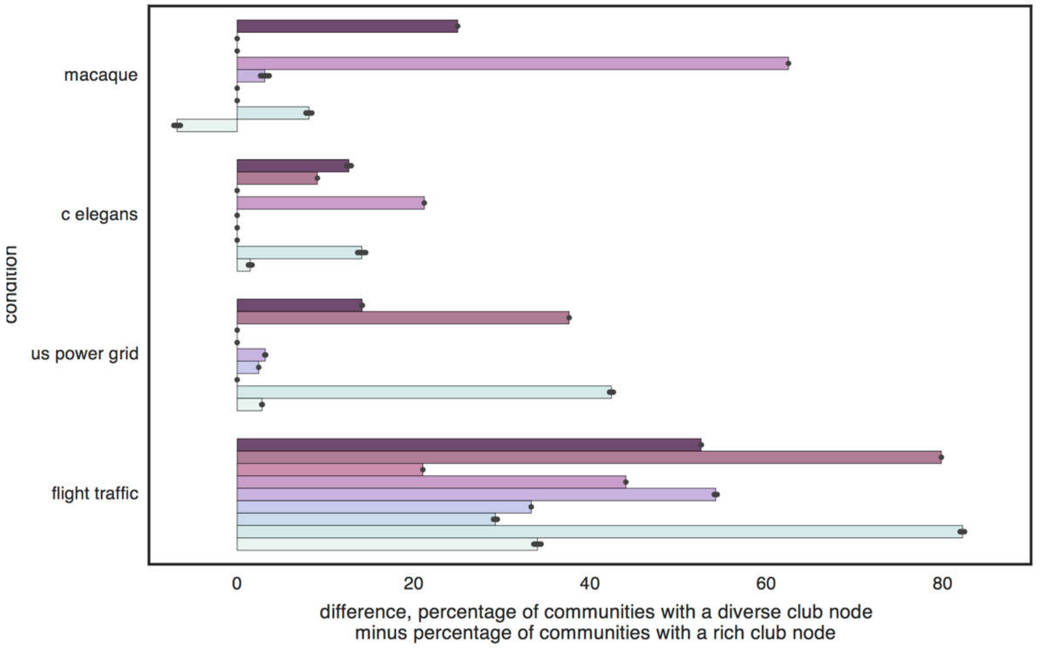

Supplementary Figure 52 I Community and club properties in structural networks. Top, the percentage of nodes that are members of both clubs. 100 means the clubs are identical, and 0 means there are no nodes that are in both clubs. Bottom, the percentage of how many communities contain a diverse club node minus the percentage of how many communities contain a rich club node. Values greater than 0 mean that the diverse club spans more communities.

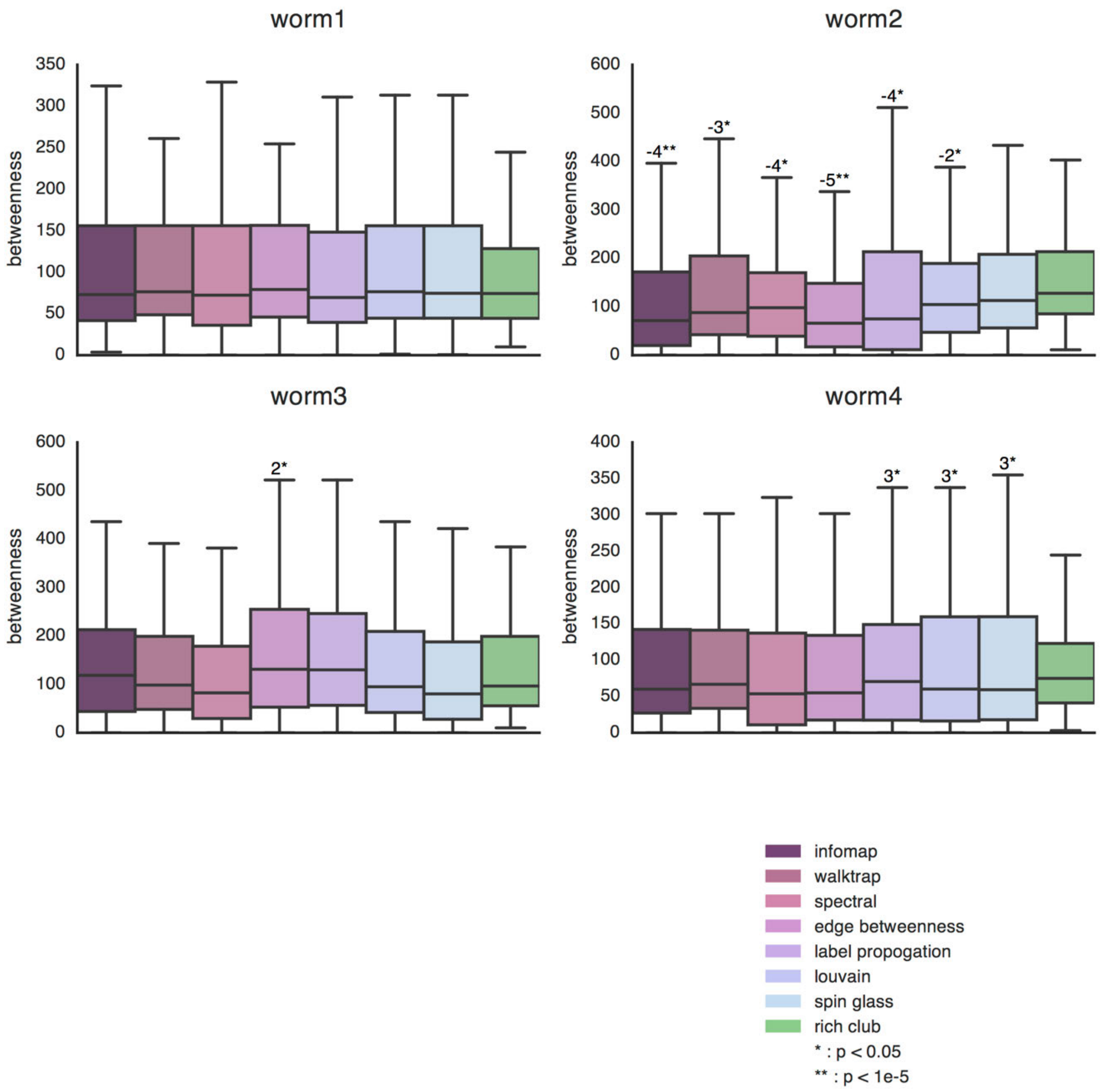

Supplementary Figure 53 l Functional c elegans betweenness centrality. Betweenness centrality measures how many shortest paths cross between all pairs of nodes through a particular node. T-tests were calculated between each diverse club and the rich club. A significant Bonferroni (number of tests=4) corrected *p* value is shown if the result was significant for that particular diverse club (i.e., for that algorithm).

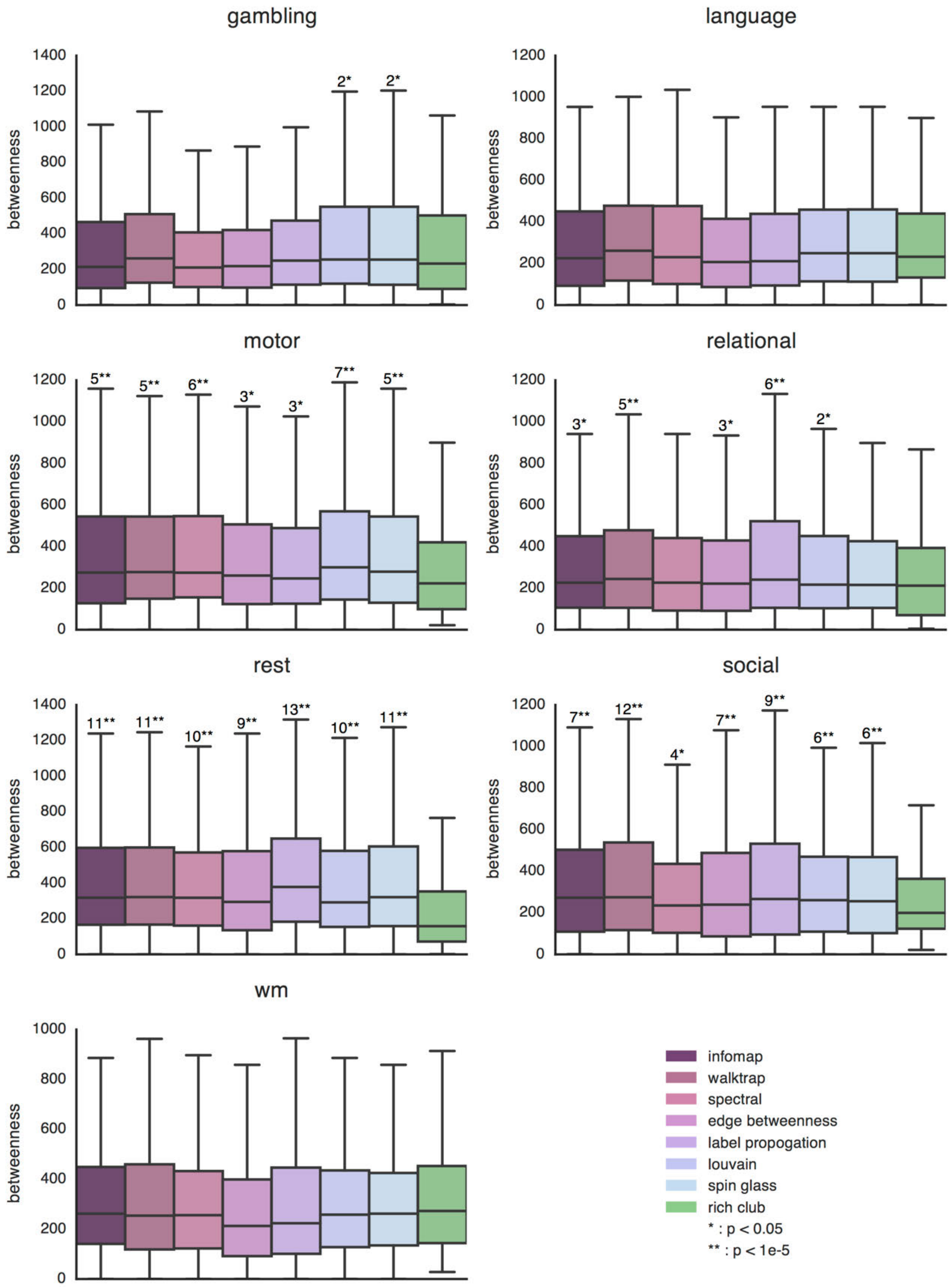

Supplementary Figure 54 I Human betweenness centrality. Betweenness centrality measures how many shortest paths between all pairs of nodes cross through a particular node. T-tests were calculated between each diverse club and the rich club. A significant Bonferroni (number of tests=7) corrected *p* value is shown if the result was significant for that particular diverse club (i.e., for that algorithm).

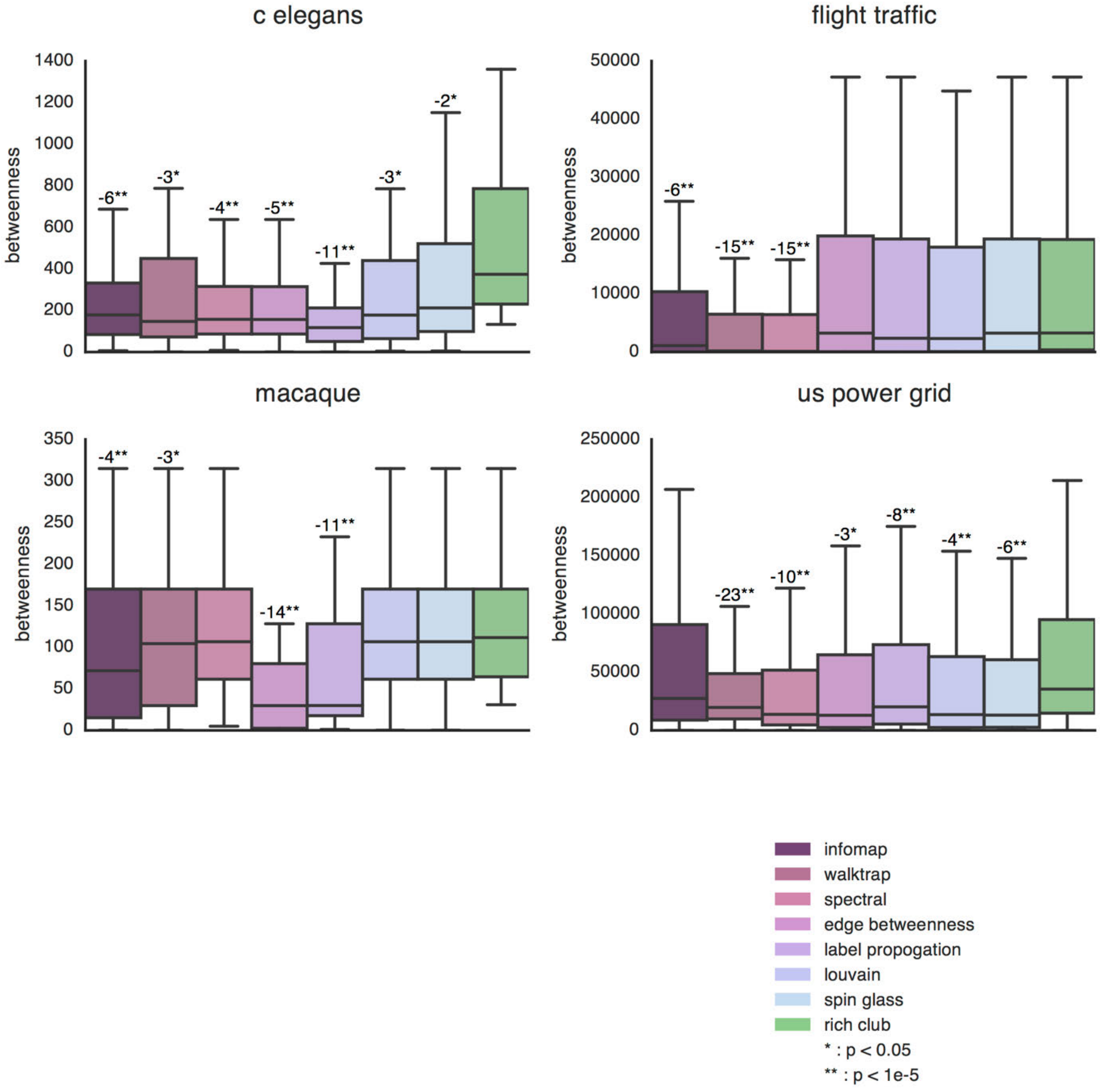

Supplementary Figure 55 I Structural networks betweenness centrality. Betweenness centrality measures how many shortest paths between all pairs of nodes cross through a particular node. T-tests were calculated between each diverse club and the rich club. A significant Bonferroni (number of tests=4) corrected *p* value is shown if the result was significant for that particular diverse club (i.e., for that algorithm).

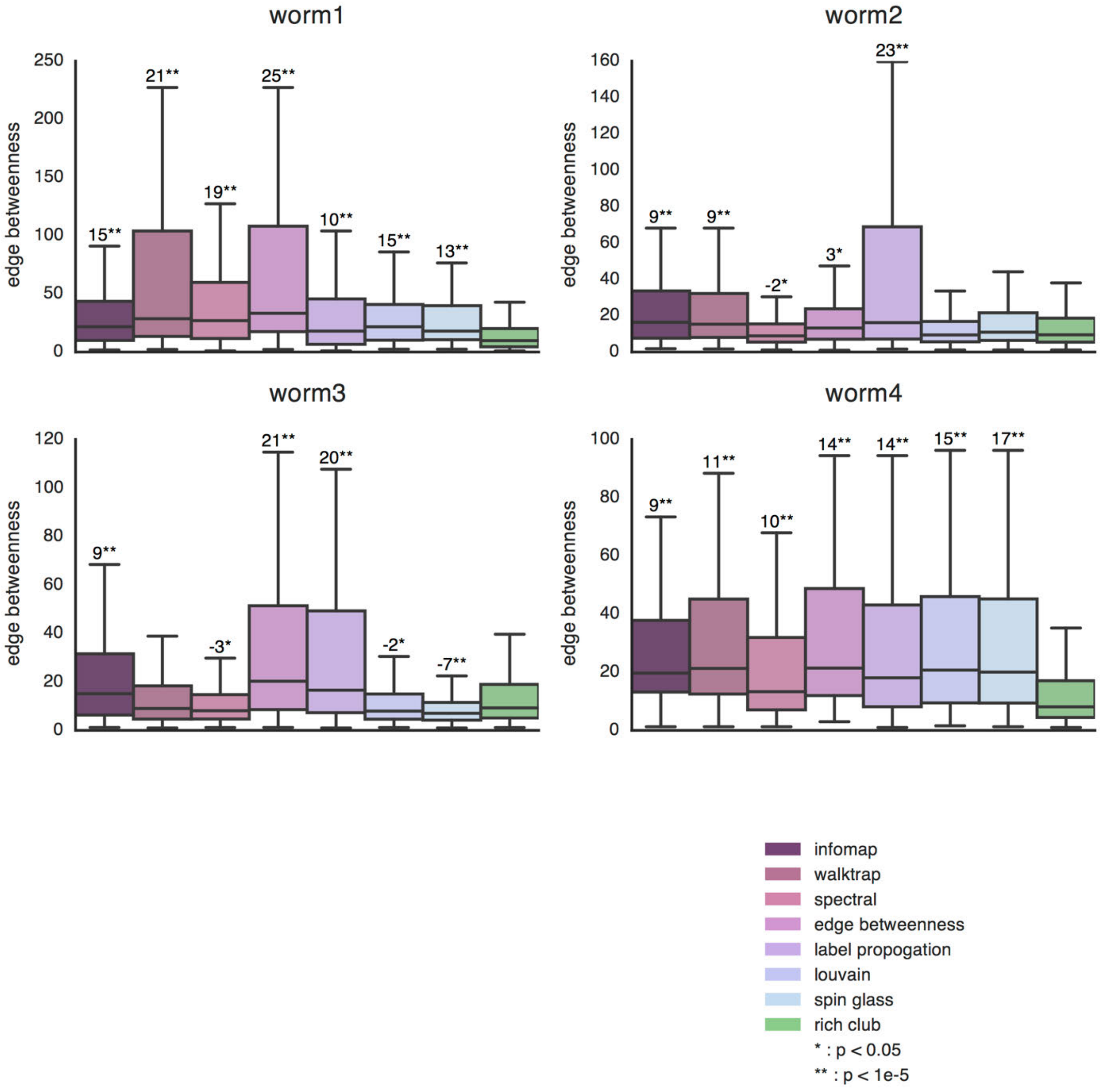

Supplementary Figure 56 I Functional c elegans edge betweenness. Edge betweenness measures how many shortest paths between all pairs of nodes cross a particular edge. Only edges between club members were included in the calculation for edge betweenness. T-tests were calculated between each diverse club and the rich club. A significant Bonferroni (number of tests=4) corrected *p* value is shown if the result was significant for that particular diverse club (i.e., for that algorithm).

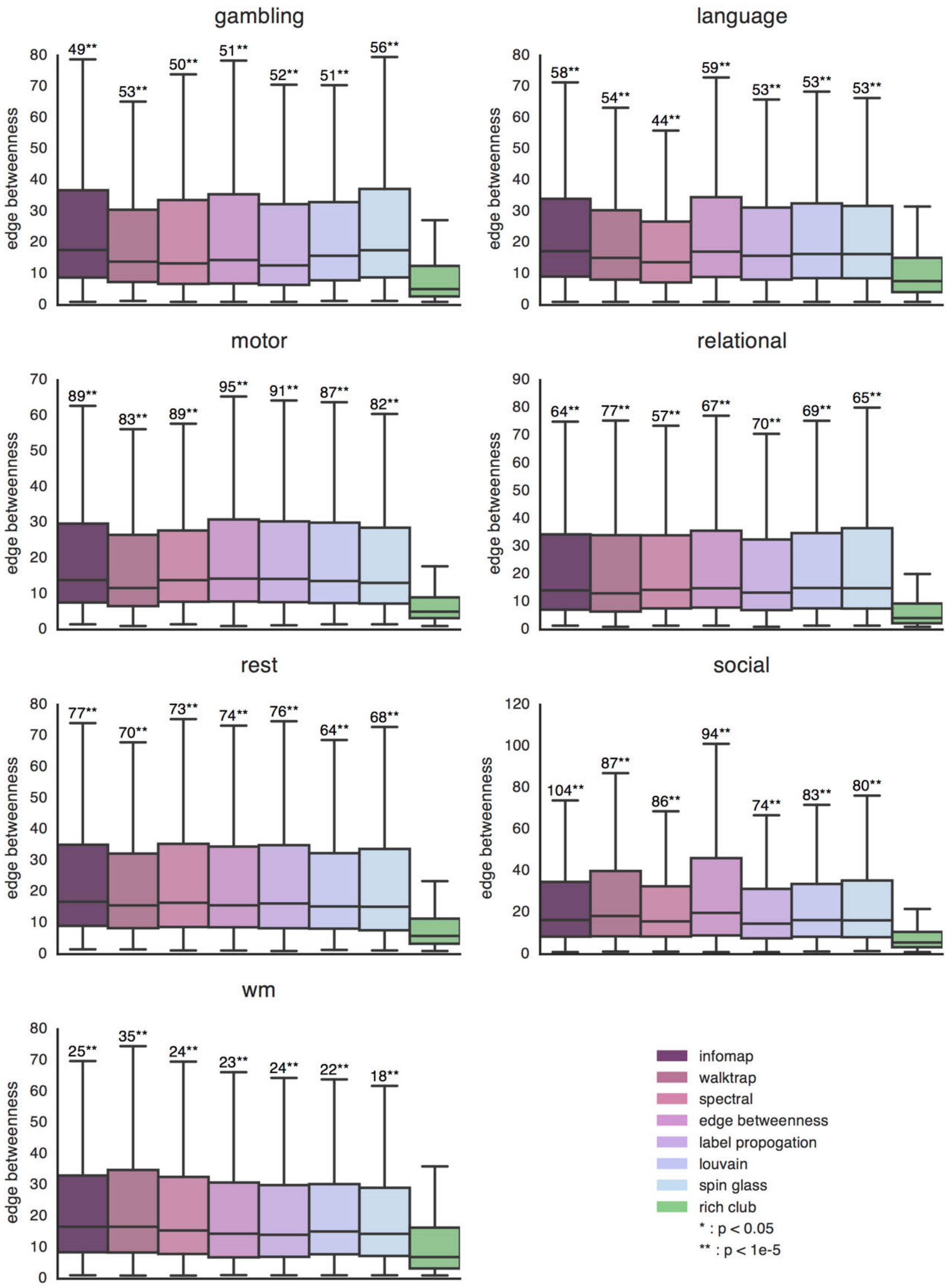

Supplementary Figure 57 I Human edge betweenness. Edge betweenness measures how many shortest paths between all pairs of nodes cross a particular edge. Only edges between club members were included in the calculation for edge betweenness. T-tests were calculated between each diverse club and the rich club. A significant Bonferroni (number of tests=7) corrected *p* value is shown if the result was significant for that particular diverse club (i.e., for that algorithm).

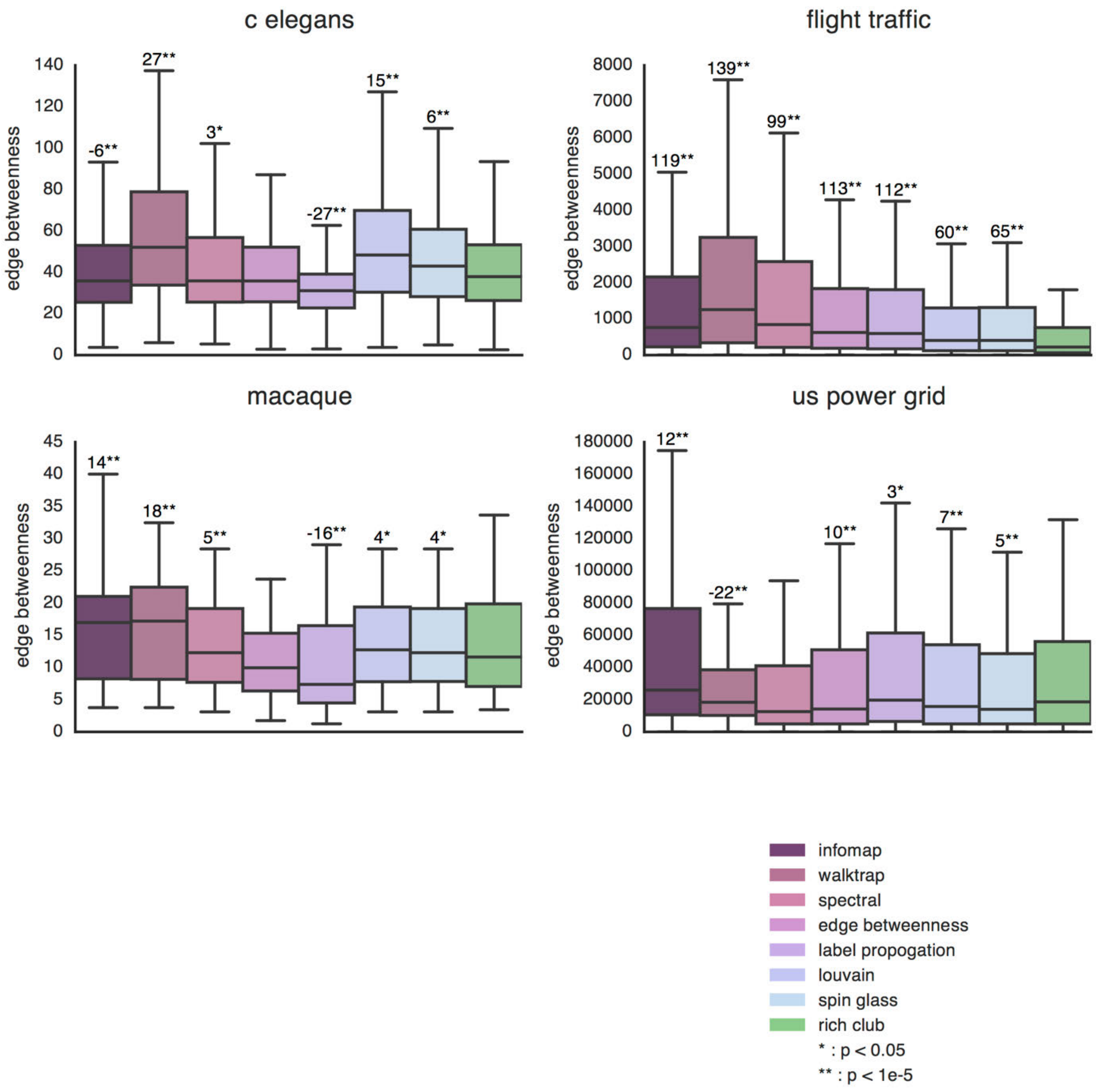

Supplementary Figure 58 I Structural networks edge betweenness. Edge betweenness measures how many shortest paths between all pairs of nodes cross a particular edge. Only edges between club members were included in the calculation for edge betweenness. T-tests were calculated between each diverse club and the rich club. A significant Bonferroni (number of tests=4) corrected *p* value is shown if the result was significant for that particular diverse club (i.e., for that algorithm).

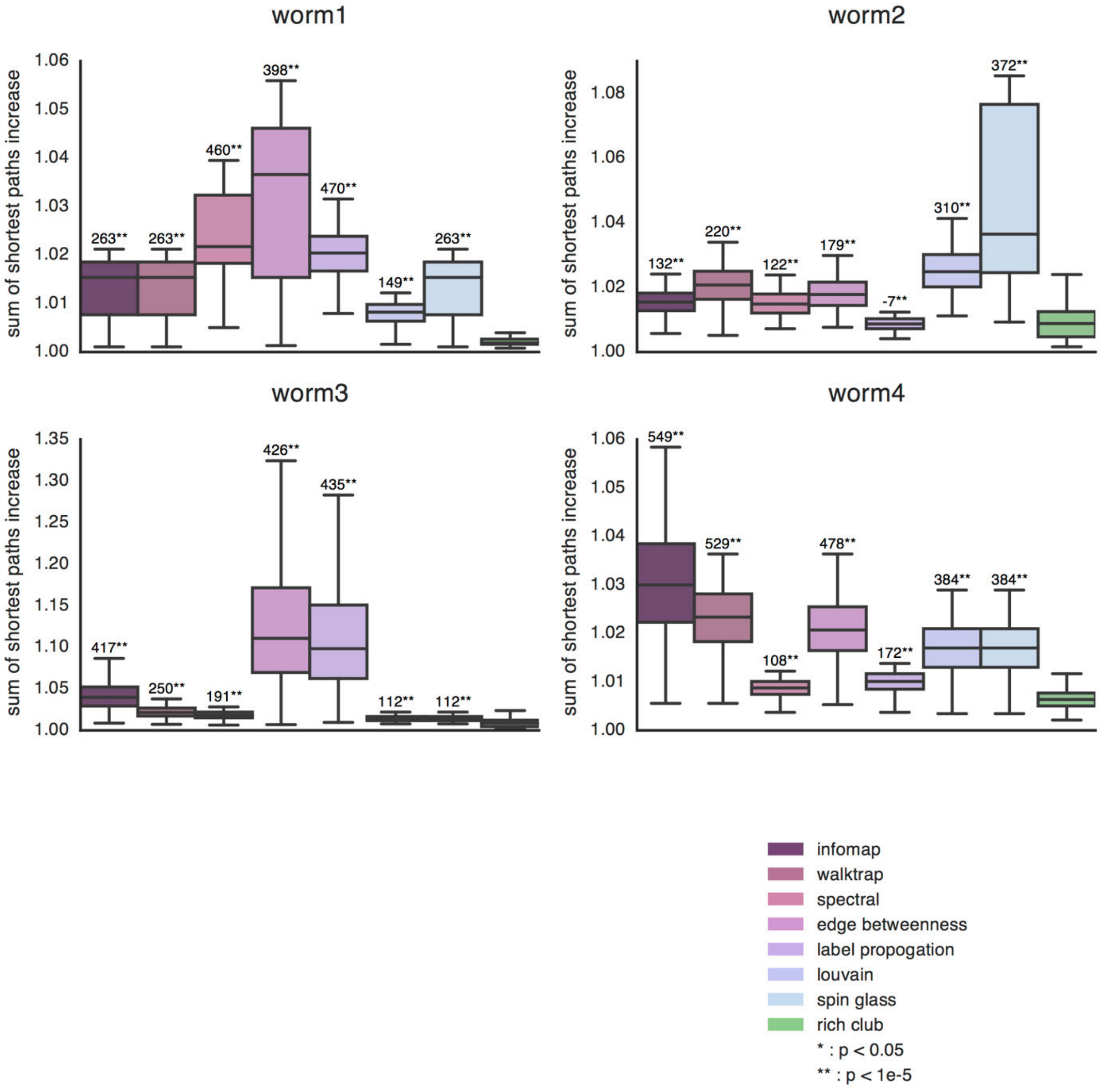

Supplementary Figure 59 I Targeted functional c elegans attacks. Sum of shortest paths after attacks on the rich club or the diverse club for every network. For each network, over 10,000 iterations, we removed anywhere (randomly) between 50 and 90 percent of edges (skipping edges that disconnected the graph into two sub-graphs) from the rich club or the diverse club. We then calculated the increase in the sum of shortest paths. An increase in the sum of shortest paths indicates decreased global efficiency. *p* values are Bonferroni corrected (number of tests=4).

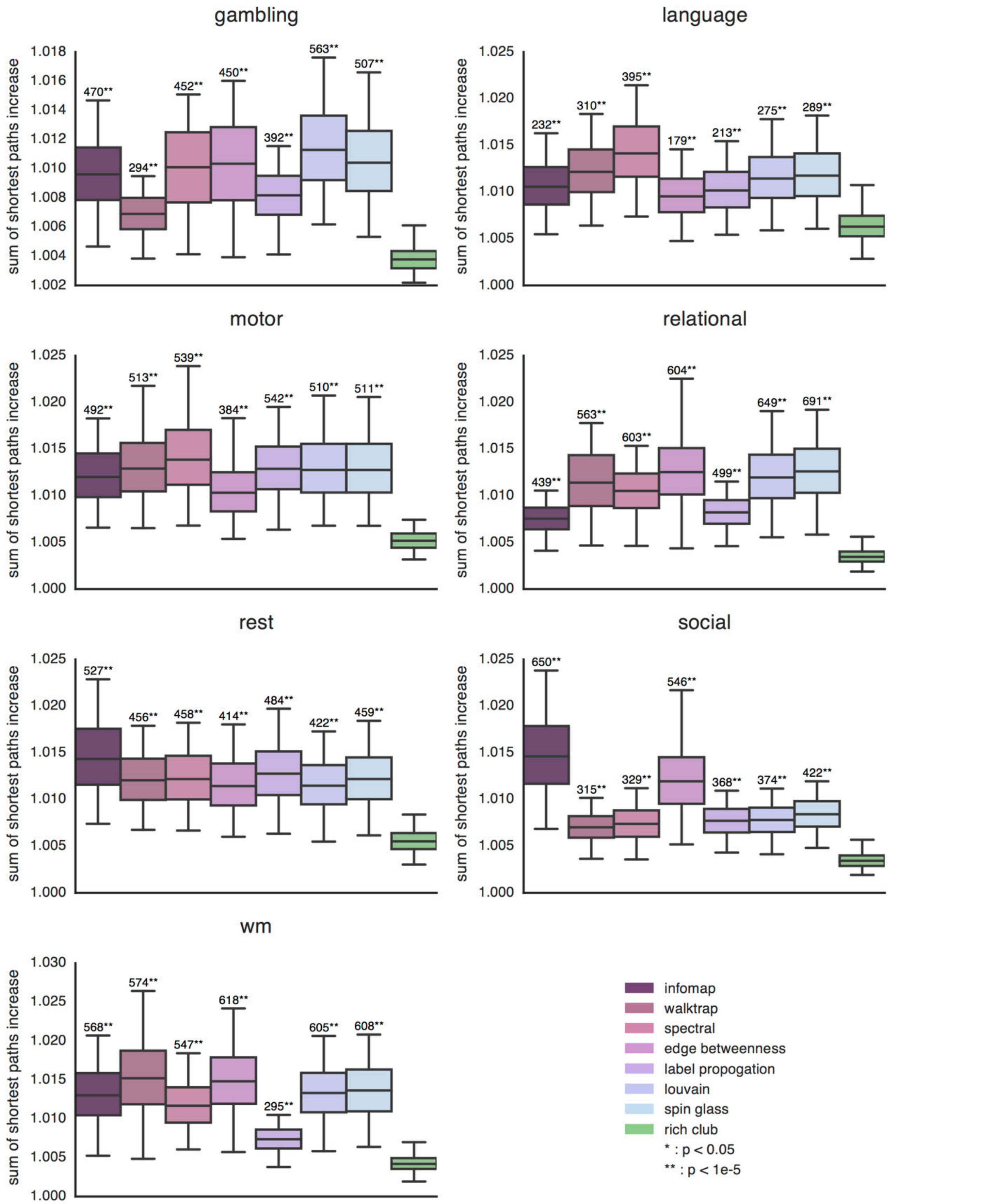

Supplementary Figure 60 I Targeted human network attacks. Sum of shortest paths after attacks on the rich club or the diverse club for every network. For each network, over 10,000 iterations, we removed anywhere (randomly) between 50 and 90 percent of edges (skipping edges that disconnected the graph into two sub-graphs) from the rich club or the diverse club. We then calculated the increase in the sum of

shortest paths. An increase in the sum of shortest paths indicates decreased global efficiency. *p* values are Bonferroni corrected (number of tests=7).

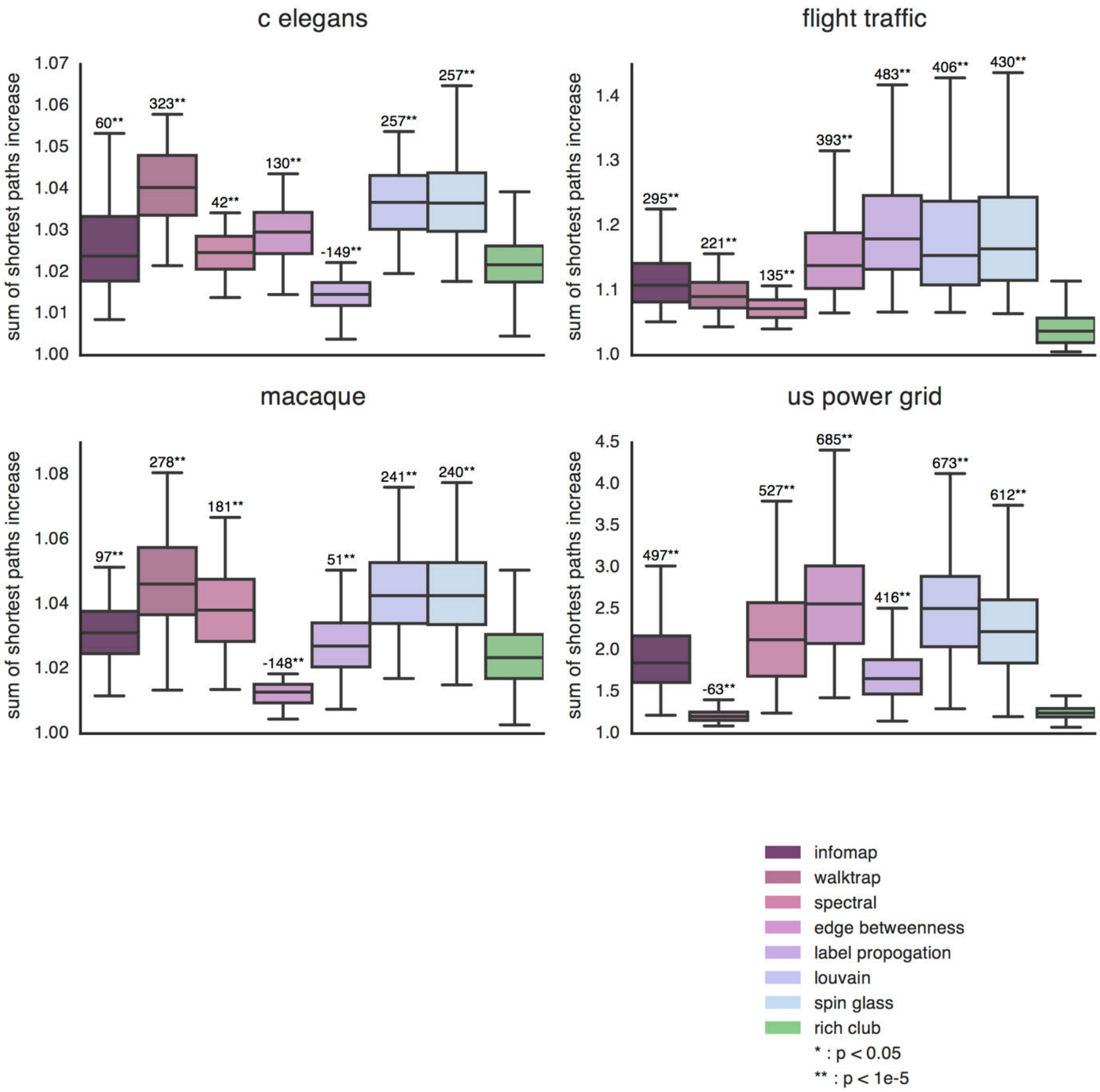

Supplementary Figure 61 I Targeted structural network attacks. Sum of shortest paths after attacks on the rich club or the diverse club for every network. For each network, over 10,000 iterations, we removed anywhere (randomly) between 50 and 90 percent of edges (skipping edges that disconnected the graph into two sub-graphs) from the rich club or the diverse club. We then calculated the increase in the sum of shortest paths. An increase in the sum of shortest paths indicates decreased global efficiency. *p* values are Bonferroni corrected (number of tests=4).

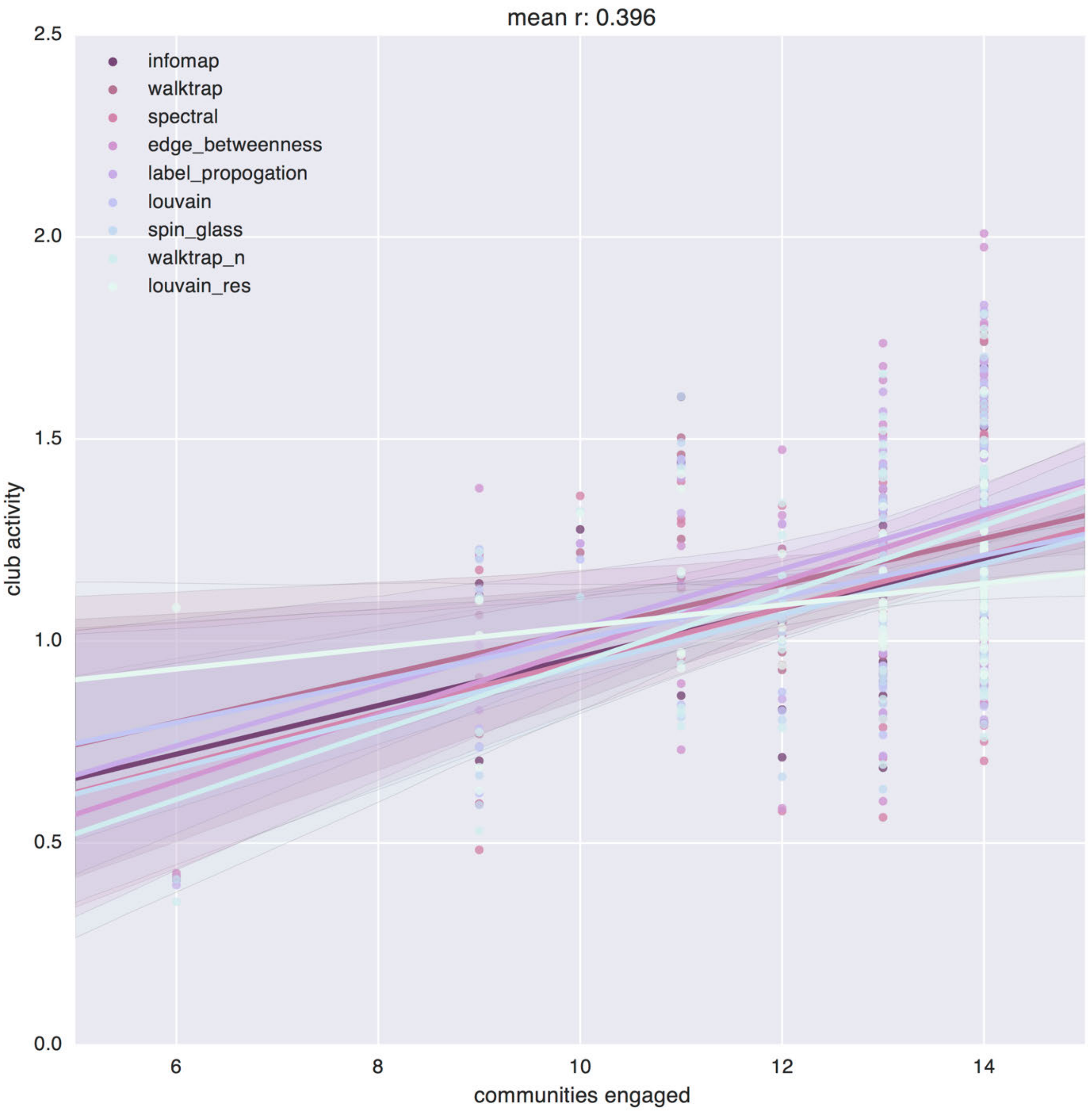

Supplementary Figure 62 I Analysis of diverse club activity across BrainMap tasks. The correlation between the number of communities engaged in a cognitive task and activity at the diverse club is shown.

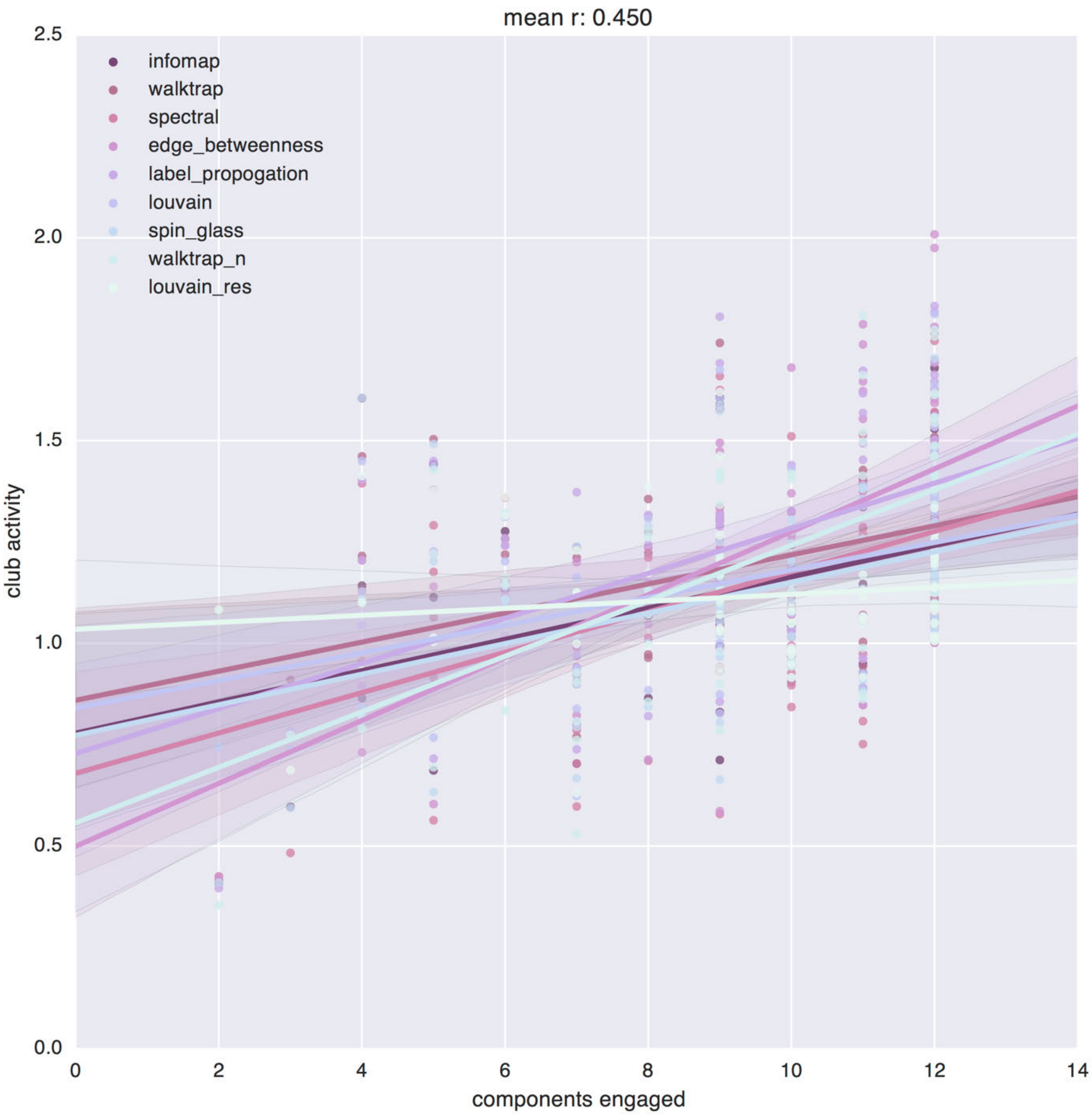

Supplementary Figure 63 I Analysis of diverse club activity across BrainMap tasks. The correlation between the number of components engaged in a cognitive task and activity at the diverse club is shown.

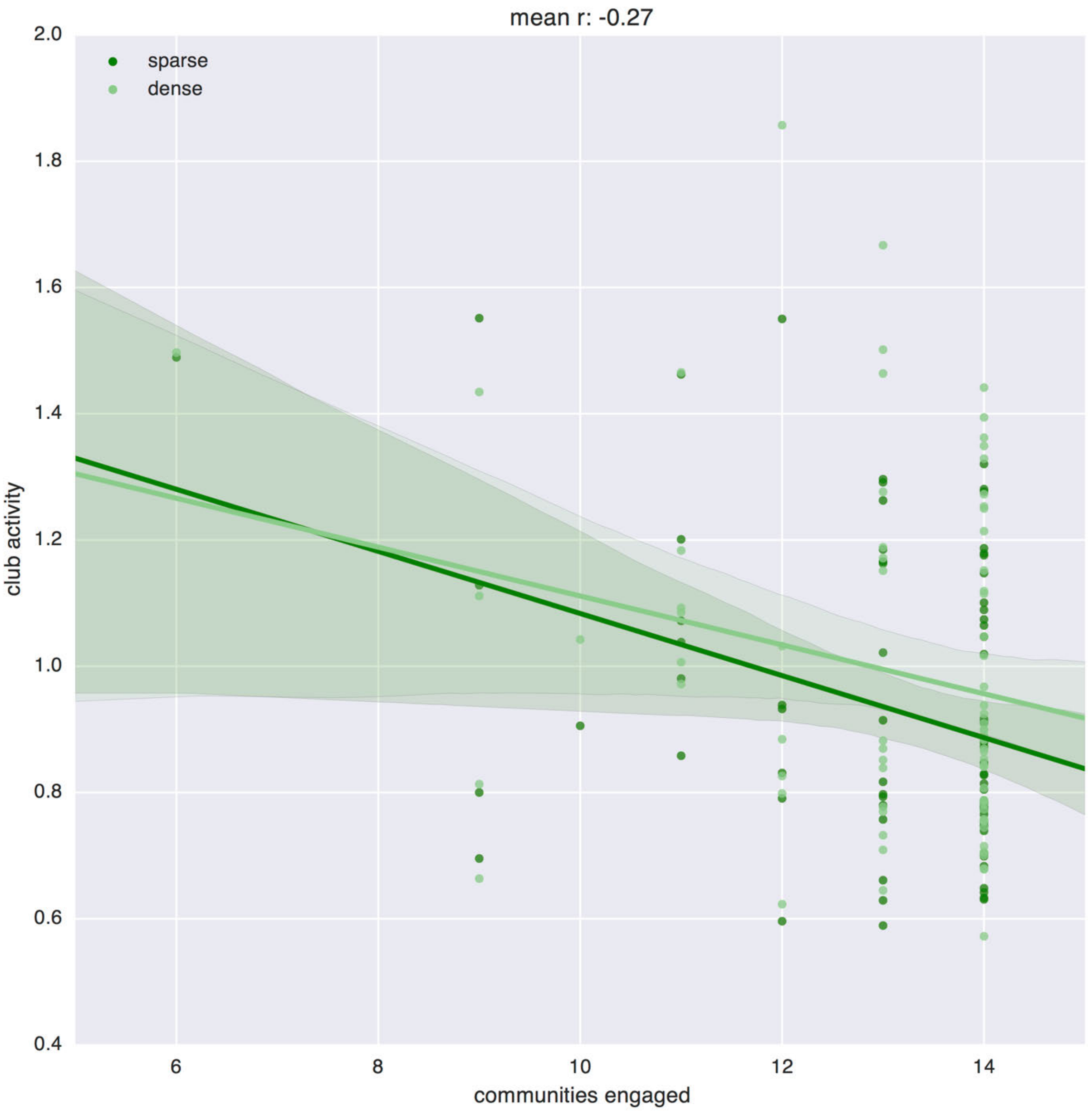

Supplementary Figure 64 I Analysis of rich club activity across BrainMap tasks. The correlation between the number of communities engaged in a cognitive task and activity at the rich club is shown.

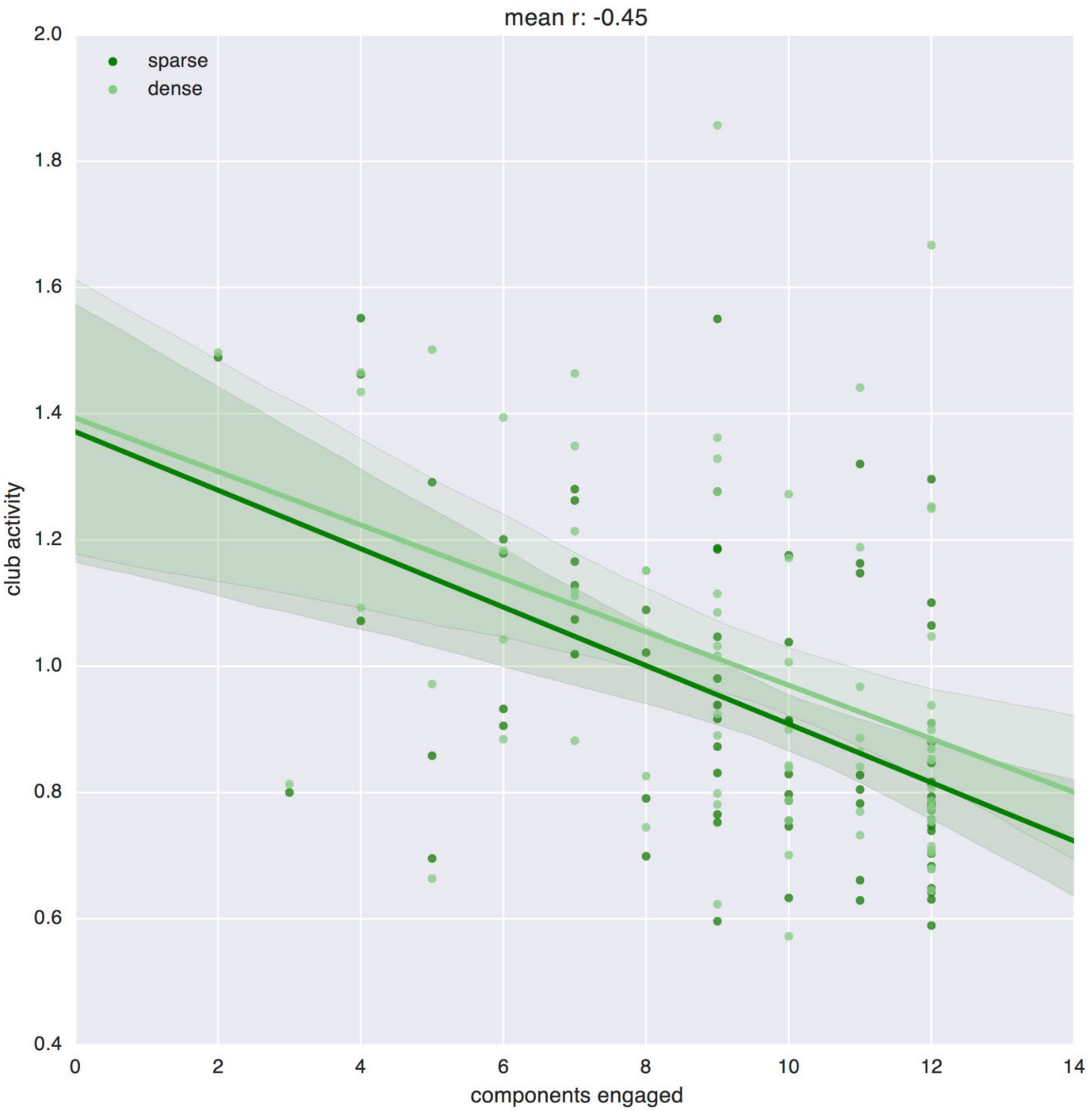

Supplementary Figure 65 I Analysis of rich club activity across BrainMap tasks. The correlation between the number of components engaged in a cognitive task and activity at the rich club is shown.

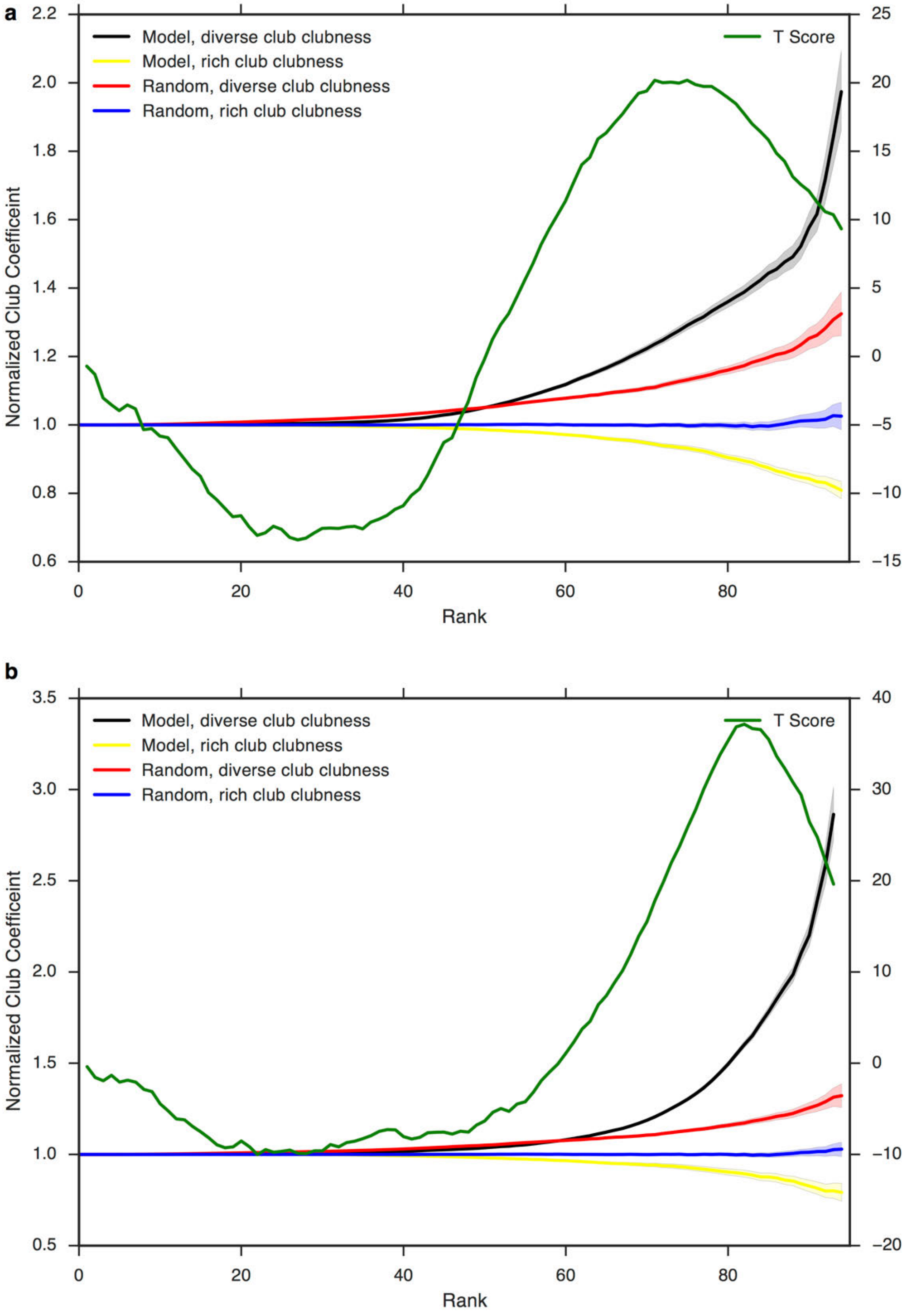

Supplementary Figure 66 | Generative models at different *Q* to *E* ratios. Alternative ratios of *Q* to *E* for the generative models (0.70(a), and 0.80(b))

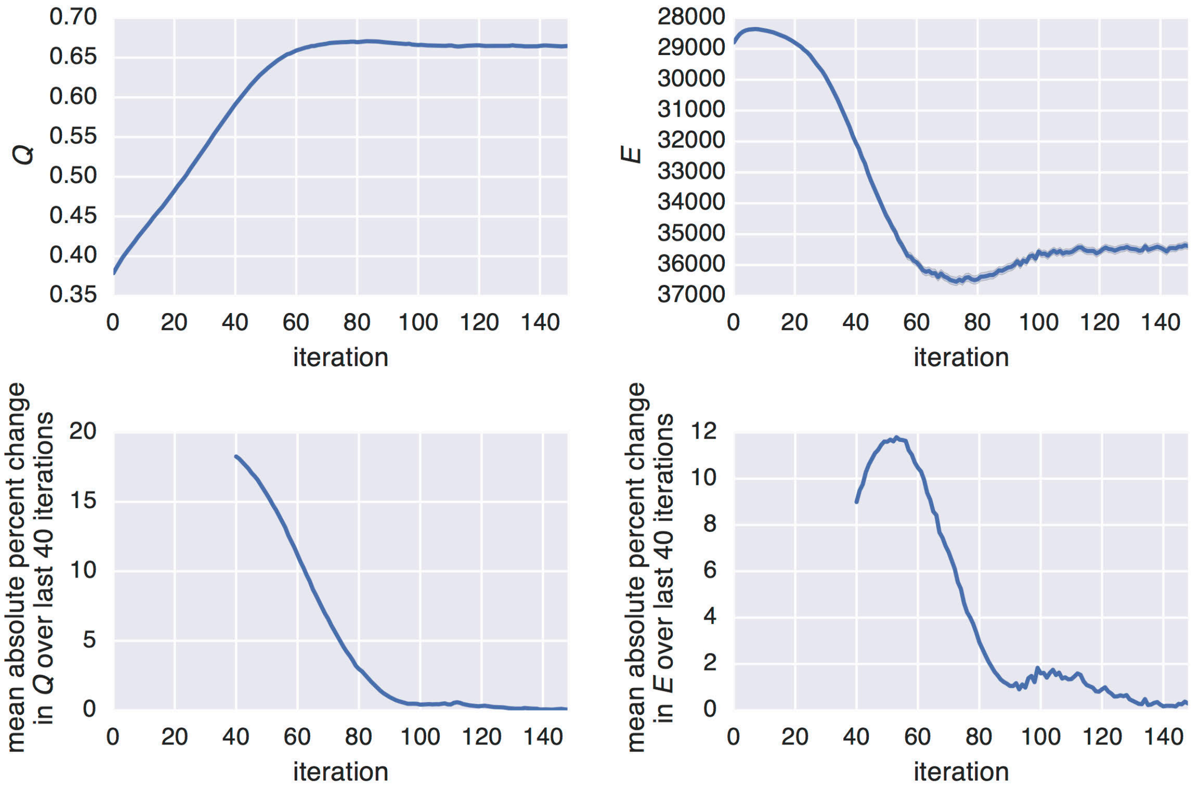

Supplementary Figure 67 I Stability analysis of the generative model. Top, *Q* and *E* are plotted across the 150 iterations of the model. Bottom, for each iteration, we calculated the absolute percentage difference between *Q* or *E* at that iteration with *Q* or the *E* in each of the previous 40 iterations. We then take the mean absolute percentage change over those 40 iterations. These values are plotted for each iteration.
